# Supplementary material for: Optimized photochemistry enables efficient analysis of dynamic RNA structuromes and interactomes in genetic and infectious diseases
Source: Nat Commun. 2021 Apr 20;12:2344. doi: 10.1038/s41467-021-22552-y (PMC8058046; doi:10.1038/s41467-021-22552-y)
Supplement: Supplementary file 1 — Supplementary Information [file 41467_2021_22552_MOESM1_ESM.pdf]

Supplementary Information

Optimized photochemistry enables efficient analysis of dynamic RNA structuromes and interactomes in genetic and infectious diseases

Minjie Zhang[1,4], Kongpan Li[1,4], Jianhui Bai[1,4], Willem A. Velema[2], Chengqing Yu[1], Ryan Van Damme[1], Wilson H. Lee[1], Maia L. Corpuz[1], Jian-fu Chen[3] and Zhipeng Lu[1,*]

[1]Department of Pharmacology and Pharmaceutical Sciences, School of Pharmacy, University of Southern California, Los Angeles, CA 90033.
[2]Institute for Molecules and Materials, Radboud University Nijmegen, The Netherlands
[3]Center for Craniofacial Molecular Biology, University of Southern California, Los Angeles, CA 90033, USA
[4]These authors contributed equally.
*Correspondence to: Z.L. at zhipengl@usc.edu

Supplementary information includes:

- Supplementary Figures (1-33)
- Supplementary Tables (1-5)
- Supplementary Notes (1-5)
- Supplementary Methods (step-by-step protocol of PARIS2)
- Supplementary References

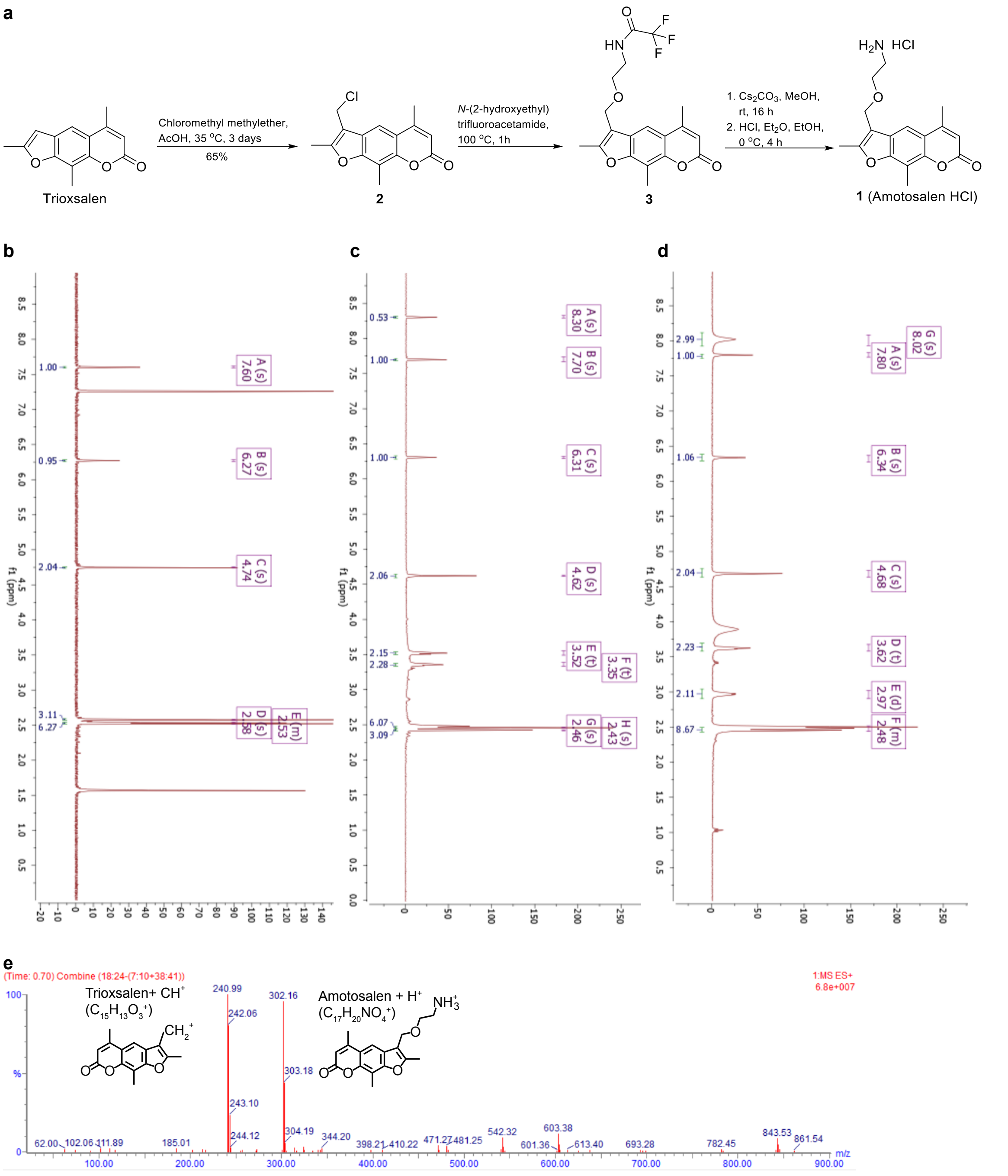

Supplementary Figure 1. Synthesis and characterization of amotosalen HCl salt. a, Synthesis route and conditions. **b-d,** ^{1}H NMR of the three compounds **2**, **3**, and **1**. **e**, Mass spectrometry analysis of compound **1**, Amotosalen HCl. Intact molecular ion and a fragment are identified. See Supplementary Methods for details on synthesis and characterization.

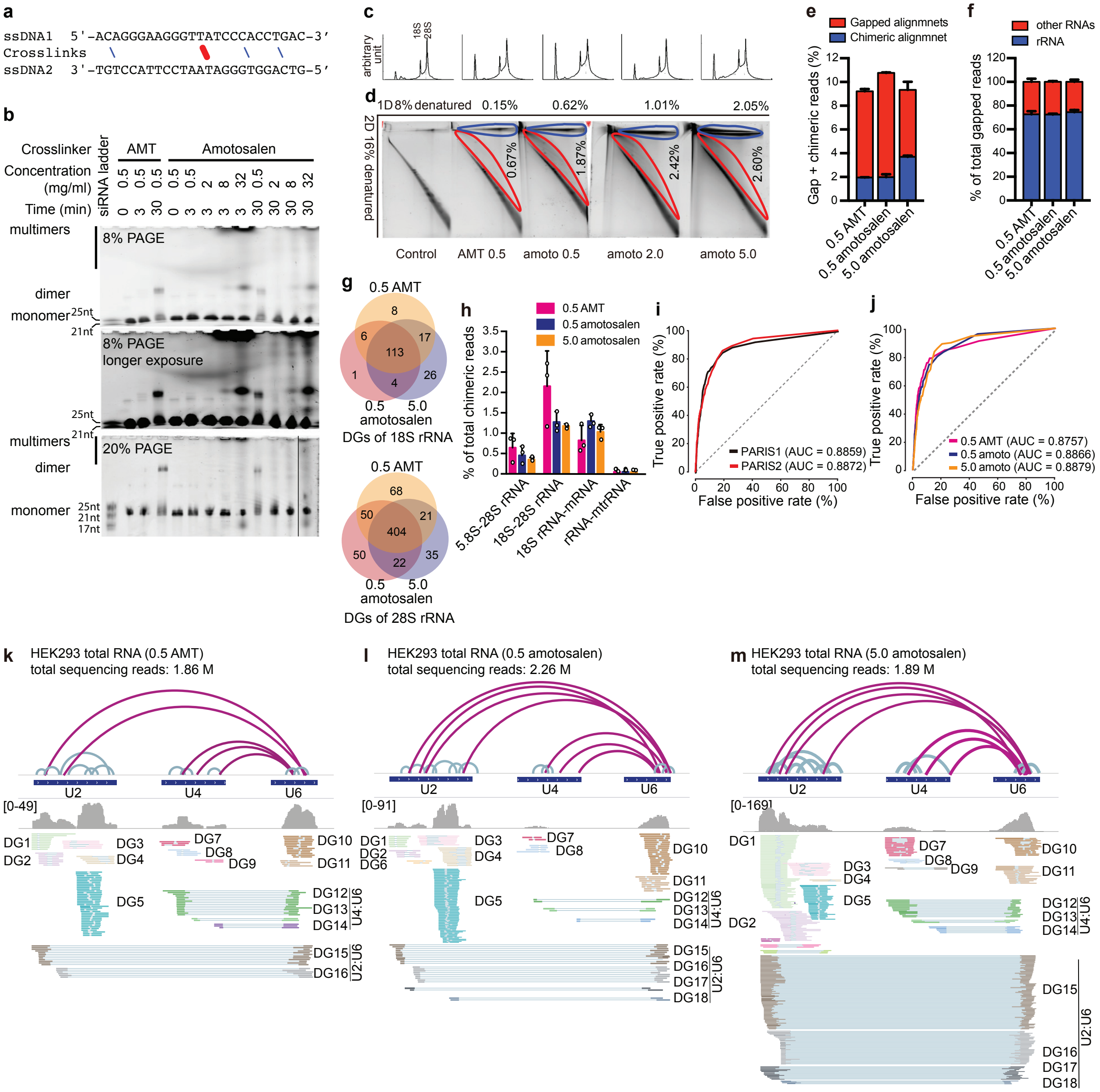

Supplementary Figure 2. In vitro and in vivo tests of amotosalen crosslinking. a, Secondary structure of the DNA oligo duplex for testing psoralen crosslinking. Blue lines: staggered T-C or C-T pairs as potential psoralen crosslinking sites. Red line: staggered T-T pair, the preferred crosslinking site. Note: there is one G-A and one G-T mismatch. **b**, DNA duplex crosslinked with AMT and amotosalen at various concentrations and different times. Crosslinked products were run in 8% or 20% urea-TBE PAGE and subjected to different exposure times. Higher amotosalen concentrations resulted in DNA oligos stuck in the well, likely due to their large size and complex structure. **c-d**, Higher psoralen concentrations increased crosslinking efficiency in vivo. Total RNA extracted from HEK293 cells using the TNA method are analyzed using TapeStation (**c**) and DD2D gel (**d**). Percentages of crosslinked RNA extracted from the upper diagonal (red outlines) and the 1D-2D interface (blue outlines) are indicated. **e**, The ratio of gapped and chimeric alignments using different crosslinkers (0.5mg/ml AMT, 0.5mg/ml amotosalen and 5.0mg/ml amotosalen). Data are mean ± s.d.; n=3 biological replicates. **f**, Human RNA types that captured by different crosslinkers. rRNA, cytoplasmic ribosomal RNA. Data are mean ± s.d.; 0.5AMT/0.5amotosalen: n=2; 5.0amotosalen: n=3. **g**, Venn diagrams showing overlapped duplex groups captured on 18S rRNA and 28S rRNA by 0.5mg/ml AMT, 0.5mg/ml and 5.0mg/ml amotosalen. Gapped/chimeric alignments were assembled to different Duplex groups (DGs) using the following criteria: arms in each DG are 50% overlapped and more than 5 reads were needed to support each DG. **h**, Percentage of different types of RNA interactions captured by 0.5mg/ml AMT, 0.5mg/ml amotosalen and 5.0mg/ml amotosalen. rRNA, cytoplasmic ribosomal RNA; mtrRNA, mitochondrial ribosomal RNA. rRNA-mtrRNA interactions served as negative control. Data are shown as the mean ± s.d. of 3 independent experiments. **i-j**, ROC analysis of PARIS2 accuracy and specificity using the 28S rRNA structure as a gold standard (PDB: 4V6X). **k-m**, Comparison of U2, U4 and U6 snRNA structures and interactions in 0.5mg/ml AMT (**k**), 0.5mg/ml amotosalen (**l**) and 5.0mg/ml amotosalen (**m**) crosslinked sample. DG1-6: U2 alternative structures; DG7-9: U4 alternative structures; DG10-11: U6 alternative structures; DG12-14: U4-U6 interactions; DG15-18: U2-U6 interactions. The structure and interaction model showed high concordance between 0.5mg/ml AMT, 0.5mg/ml amotosalen and 5.0mg/ml amotosalen crosslinking. The experiments in **b, d** were independently repeated twice with similar results.

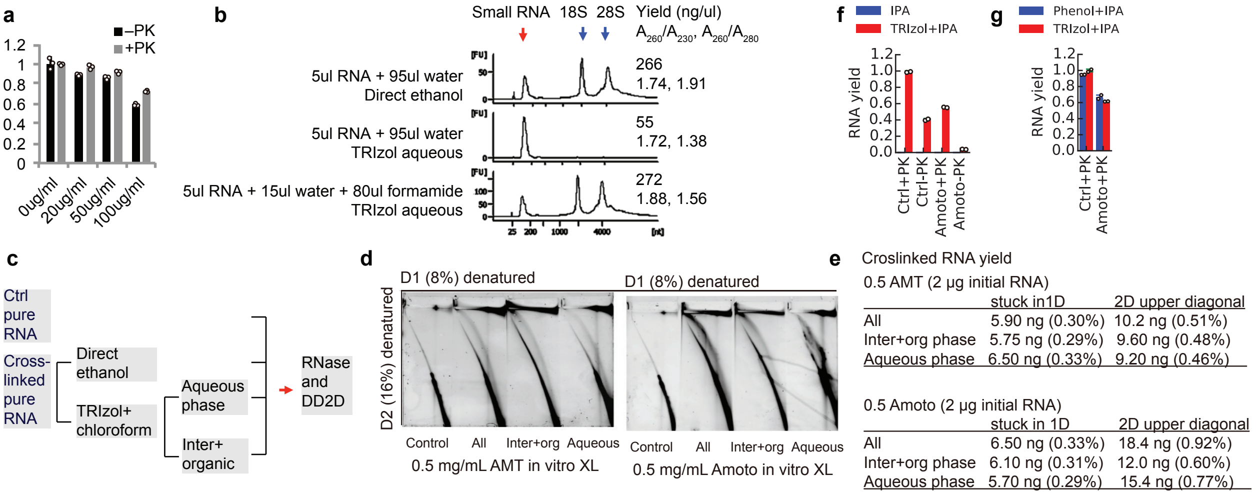

Supplementary Figure 3. TNA: A new method to recover crosslinked RNA from cells. a, Psoralen crosslinking reduces yield of TRIzol extracted RNA. AMT crosslinking was performed in triplicate for four conditions: without AMT, with AMT at 20, 50 and 100µg/ml. PK, Proteinase K treatment. Data are mean ± s.d.; n=3, biological replicates. **b**, Phase partition of in vitro crosslinked RNA in TRIzol+chloroform with or without formamide. Five µg total HEK293 RNA was in vitro crosslinked with 0.5mg/ml AMT. After crosslinking, RNA was either directly precipitated with ethanol, or supplemented with water or formamide and then purified with TRIzol. **c**, Experimental design to test how much crosslinked RNA was in the aqueous vs. inter+organic phase. Briefly, crosslinked pure RNA was either directly precipitated with ethanol or purified using TRIzol+chloroform. All RNA samples were digested with RNase III and subjected to DD2D gel purification. **d**, Crosslinked RNA partitioned to inter+organic phase. 2D gel purification of crosslinked RNA from non-crosslinked, all crosslinked, crosslinked in the inter+organic phase, crosslinked in the aqueous phase by standard Trizol purification. 2µg RNA samples were crosslinked with 0.5mg/ml AMT (left panel) or 0.5mg/ml amotosalen (right panel). The experiments were independently repeated twice with similar results. **e**, Recovery of crosslinked RNA from the 1D-stuck and 2D-upper diagonal were quantified. Total yield was similar for the three crosslinked samples with identical input amount. **f**, Total nucleic acids can be purified after PK treatment and alcohol precipitation in presence of TRIzol. IPA, isopropanol. **g**, Total nucleic acids can be purified using isopropanol and phenol, after PK treatment. Yields of both methods were similar, suggesting that phenol was the primary component necessary in keeping residual proteins in solution, while alcohol precipitated nucleic acids. Data in **f** and **g** are mean ± s.d.; n=2, biological replicates.

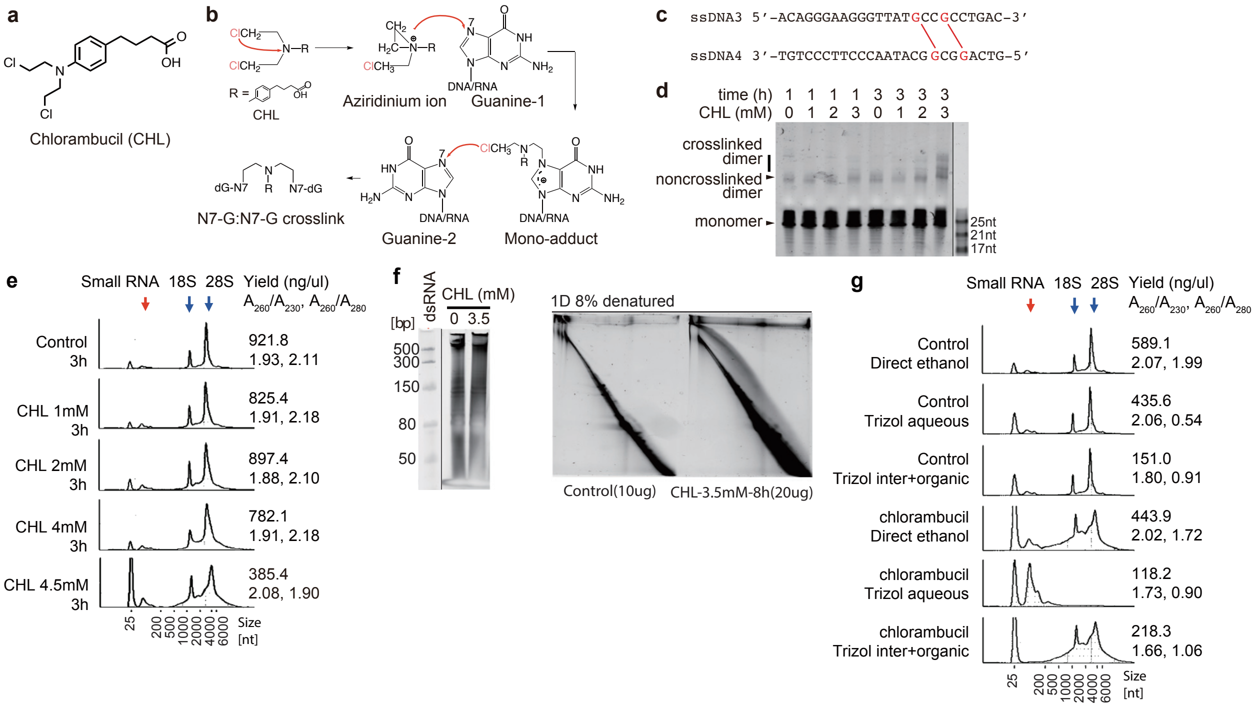

Supplementary Figure 4. Chlorambucil crosslinked RNA was hydrophobic. a, Chemical structure of chlorambucil (CHL). **b**, Nucleic acids crosslinking mechanism of chlorambucil. Chlorambucil, like many other nitrogen mustards, forms aziridinium ions by intramolecular displacement of the chloride by the amine nitrogen. This aziridinium group then alkylates DNA once it is attacked by the N-7 nucleophilic center on the guanine base to become a mono-adduct. A second attack after the displacement of the second chlorine results in the formation of inter-strand cross-links. **c**, Synthetic 25-mer DNA duplex used to test CHL crosslinking. The red lines indicate predicted guanine-guanine DNA inter-strand crosslinking (an 1,3 cross-link, G:G3). **d**,15% gel electrophoresis of 25-mer DNA oligos crosslinked with different concentrations of CHL at various times. **e**, TapeStation electropherograms of crosslinked total RNA by different concentrations of CHL. **f**, DD2D gel system showing CHL in vivo crosslinked total RNA. **g**, CHL crosslinked total RNA partitioned into the interphase during TRIzol extraction. The experiments in **d, f** were independently repeated twice with similar results.

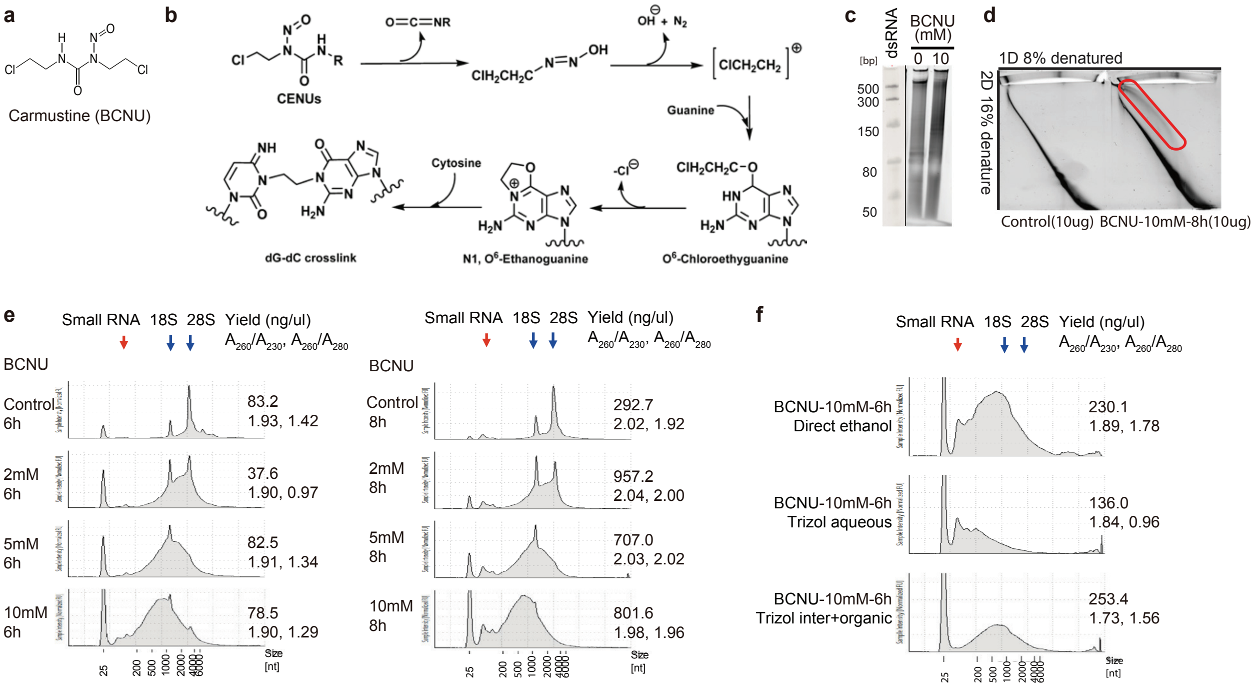

Supplementary Figure 5. BCNU crosslinked RNA was hydrophobic. **a**, Chemical structure of carmustine (BCNU). **b**, Molecular mechanisms of chloroethylnitrosoureas (CENUs) crosslinking G-C base pairs. BCNU is one of the CENUs, where R=CH$_2$CH$_2$Cl. The chloroethyl diazonium ion produced by the decomposition of CENUsalkylates guanine on the O6 site formed O6-chloroethylguanine (O6-ClEt-Gua), followed by further alkylation of the complementary cytidine on the N3 site via a cationic intermediate, N1,O6-ethanoguanine. **c-d**, DD2D gel showing BCNU crosslinked total RNA. 1st dimension gel (**c**); 2nd dimension gel (**d**). The experiments in **c, d** were independently repeated twice with similar results. **e**, TapeStation profiles of BCNU crosslinked total RNA under different conditions. Pure RNA from HEK293T cells was crosslinked with 2, 5 or 10 mM BCNU at 37°C in the dark for either 6 or 8 hours. Long-term crosslinking induced partial RNA degradation. **f**, TapeStation profiles of different phases from TRIzol extracted BCNU crosslinked total RNA. Pure RNA was crosslinked with 10mM BCNU for 6 hours. After crosslinking, sample was divided equally into 2 tubes, one for the direct ethanol precipitation, another for TRIzol extraction, which were further divided into the aqueous phase and the inter+organic phase. RNA in two phases were precipitated by ethanol. Larger RNA partitioned to inter+organic phase from aqueous phase.

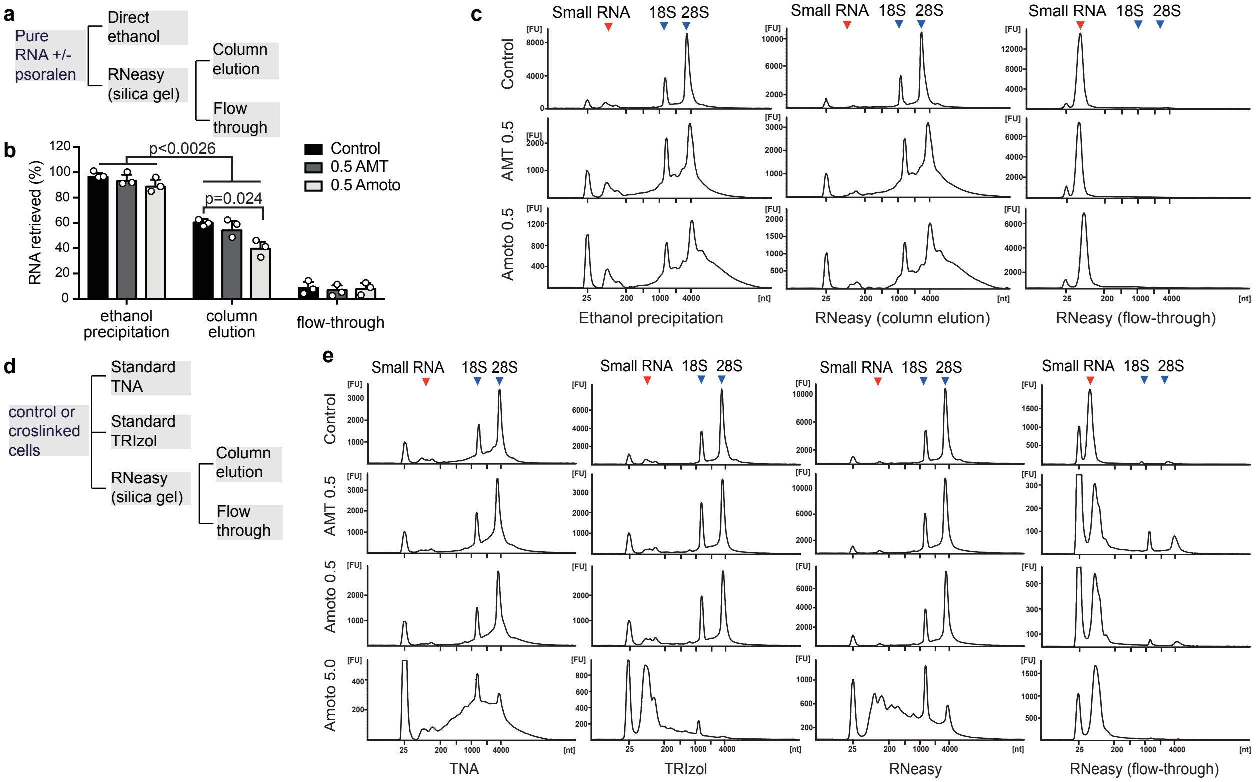

Supplementary Figure 6. Comparison among TRIzol, TNA and RNeasy in recovering crosslinked RNA from in vitro or in vivo sources. a, Diagram for the experimental design. Purified total RNA was used directly or crosslinked with 0.5mg/ml AMT or amotosalen. Then RNA is directly precipitated out of solution or purified using the RNeasy kit. Flow-through fraction was also precipitated using ethanol. **b**, Quantity of RNA recovered ratio from 5µg of control and **i**n vitro crosslinked total RNA. Data are mean ± s.d.; n=3, biological replicates. two-tailed, unpaired t-test. **c**, Size distribution of RNA after purification using direct ethanol precipitation or the RNeasy kit. Small RNA, RNAs in the range of 50-300nt, such as tRNAs, snRNAs and snoRNAs. The smear, especially the tail after the 28S peak was indicative of successful crosslinking. **d**, Diagram for the experimental design testing various methods in extracting crosslinked RNA from cells. **e**, Size distribution of RNA after purification using different methods. TNA consistently outperforms other methods in the extraction of crosslinked RNA. For the TNA method, DNA was removed before quantification.

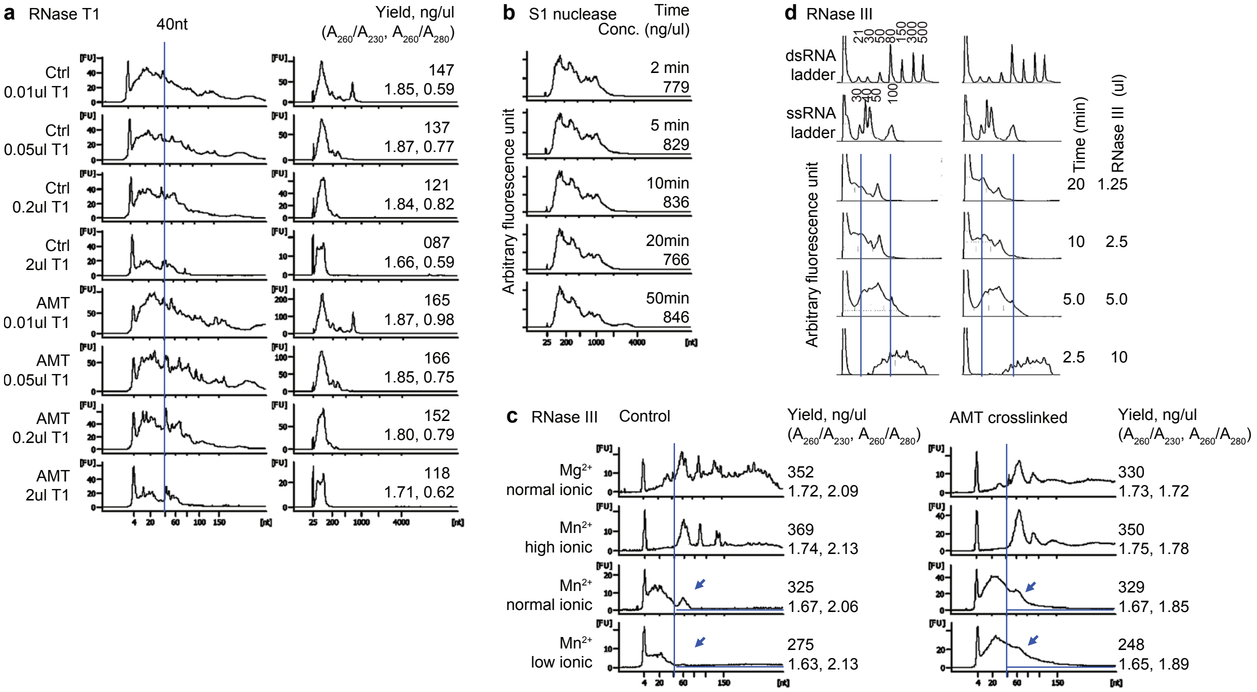

Supplementary Figure 7. Optimization of nuclease fragmentation of crosslinked RNA. **a**, RNase T1 treatment reduces total RNA amount and size, but the distribution is still broad. **b**, Extensive S1/PK digestion does not reduce size or yield significantly. **c**, AMT crosslinking affects RNase III digestion. Buffer conditions were as follows: Mg^{2+} normal ionic strength: 50mM Tris-HCl, 1mM DTT, 50mM NaCl, pH 7.5 at 25°C + 5 mM $MgCl_2$. Mn^{2+} low ionic: 5mM Tris-HCl, 1mM DTT, pH 8.0 at 25°C + 5mM $MnCl_2$. Mn^{2+} normal ionic: 50mM Tris-HCl, 1mM DTT, 50mM NaCl, pH 7.5 at 25°C + 5 mM $MnCl_2$. Mn^{2+} high ionic: 50mM Tris-HCl, 1mM DTT, 1M NaCl, pH 7.5 at 25°C + 5mM $MnCl_2$. The blue arrows point to the differences induced by crosslinking, which is more obvious after RNase III digestion under lower ionic strength. **d**, RNase III reaction kinetics affect RNA fragment size. Reaction conditions: 5μg RNA sample from the TNA method was treated with variable amount of RNase III in low ionic strength buffer for various times. The ssRNA ladder and samples were denatured before loading into TapeStation, whereas the dsRNA ladder was not.

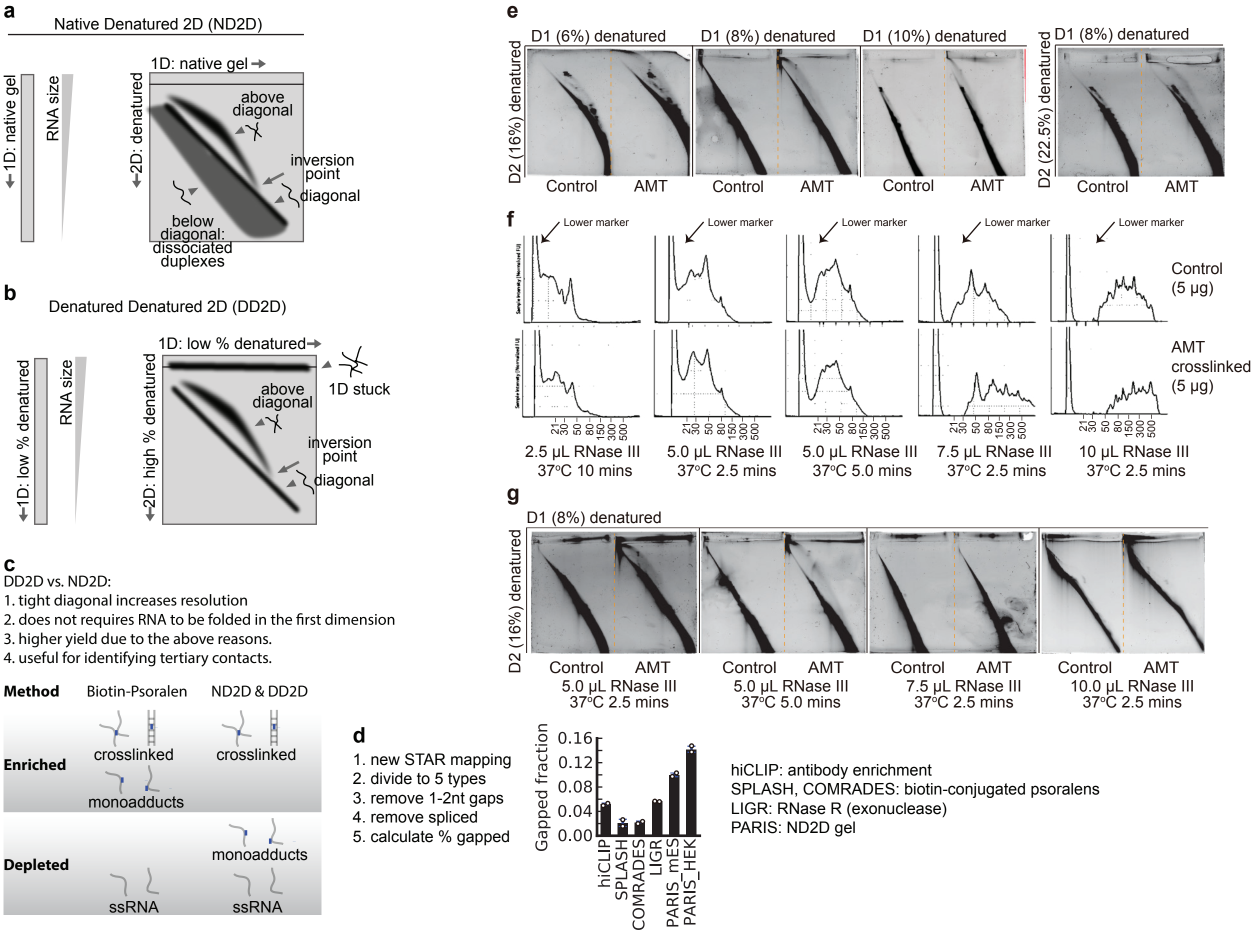

Supplementary Figure 8. Optimization of the DD2D gel method. a, Diagram for the ND2D gel system. In the first dimension, RNA separates roughly based on size. In the second dimension, RNA further separates based on shape where crosslinked fragments migrate much slower due to much larger hydrated radius. Inversion point is at an RNA size where the increase in hydration radius is no longer contributing to the migration. Noncrosslinked RNA duplexes that run together in the native first dimension would dissociate in the second dimension, causing downward smear in the second dimension. **b**, Diagram for the DD2D gel system. Like the ND2D system, RNA separates based on size in the first dimension. Here the shape also contributes to the migration due to denaturation, but the lower gel density (bigger pore size) reduces the effect of the shape of crosslinked RNA. In the second dimension, the higher gel density (smaller pore size) increases friction for the crosslinked RNA fragments, substantially more than in noncrosslinked ones. The RNA stuck in the first dimension may be more structured RNA that could not enter the second dimension of higher percentage gels. **c**, Comparison of ND2D and DD2D gels. It is worth noting that the DD2D gel does not require crosslinked RNA to be strongly base paired and is therefore also useful for identifying crosslinkable tertiary contacts. **d**, PARIS outperforms other methods in terms of percentage of gapped alignments. Fraction of gapped alignments for various methods were calculated as (gapped alignments + RNA-RNA interactions) / total alignments. Short indels are discarded since they are often due to sequencing errors, which are especially high in the presence of psoralen adducts. The following data were used. PARIS (HEK293, Lu et al. 2016), PARIS (mES, Lu et al. 2016), LIGR (Sharma et al. 2016), SPLASH (Aw et al. 2016), COMRADES (Ziv et al. 2018). Data are mean ± s.d.; n=2 of sequencing replicates. **e**, Testing combinations of various gel concentrations for the first and second dimensions. **f**, Analysis of the RNase III digested total RNA with different shortcut conditions. **g**. DD2D purification of the crosslinked RNA with different RNase III conditions. The experiments in **e**, **g** were independently repeated twice with similar results.

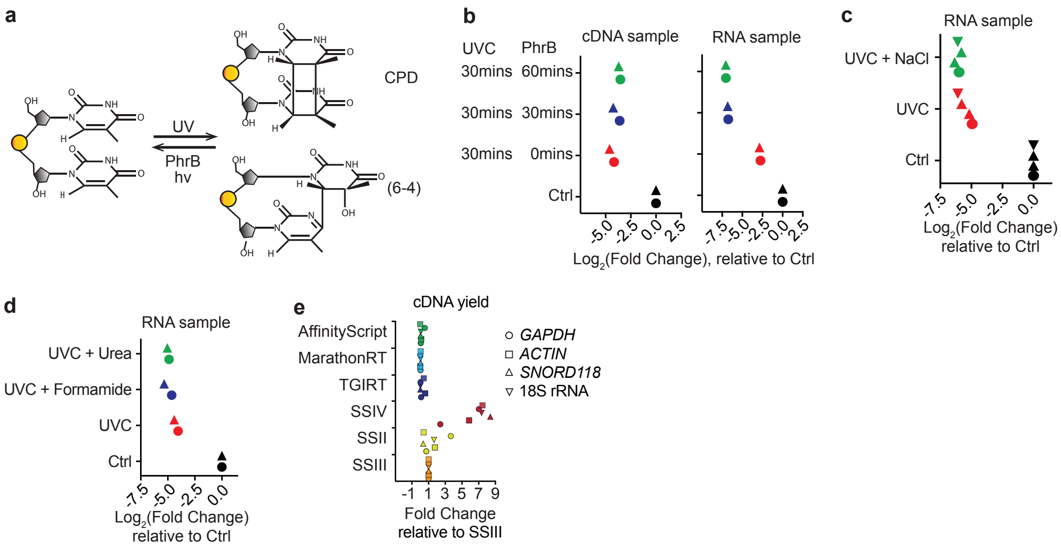

Supplementary Figure 9. Effects of various condtions on the repair, prevention or bypass of UVC damages on RNA. **a**, Funtion of photolyase in DNA damage repair. UV irradiation produces heavy DNA damages, such as cyclobutane pyrimidine dimer (CPD) and (6-4) lesion. These DNA damages can be repaired by the enzyme photolyase (PhrB) in the presence of long-wavelength light (e.g. UVA and blue). **b**, PhrB cannot repair UVC damaged RNA. UVC damage repair efficiency showed by Ct value obtained by qPCR. cDNA sample is generated from total RNA of HEK293 cells by SSIII with random hexamer. cDNA and RNA sample is irradiated by 254 nm UV for 30 mins to introduce damage. Then the enzyme PhrB is used to repair UVC damage under 365 nm UV for 30 and 60 mins respectively. Log2-fold change values determined by qPCR and normalized to control sample. **c**, High salt reverse crosslinking condition with UVC. 1M NaCl was added to reversal reaction, to help RNA to form stable secondary structures. UVC 254nm 30min damage was tested by cDNA yield of *ACTB*. Log2-fold change values determined by qPCR and normalized to control sample. **d**, UVC reversal of crosslinking with denaturing buffer, such as 50% formamide or 4M Urea. UVC 254nm 30min damage was tested by cDNA yield of *ACTB* mRNA. Log2-fold change values determined by qPCR and normalized to control sample. **e**, Bypass UVC damages by different reverse transcriptases. cDNA yield of *GAPDH* (circles), *beta-ACTIN* (square), *SNORD118* (up triangle) and 18S rRNA (down triangle) were tested to show the bypass by reverse transcriptases after UVC damage. UVC damage is introduced by 254 nm UV irradiated RNA for 30 mins. cDNA yield was determined as the Ct value obtained by qPCR of the reverse transcriptional cDNA and normalized to a Superscript III condition. Standard buffers and reaction conditions were used unless otherwise indicated. Two replicates in panel **b, c** and **d** are shown for each condition.

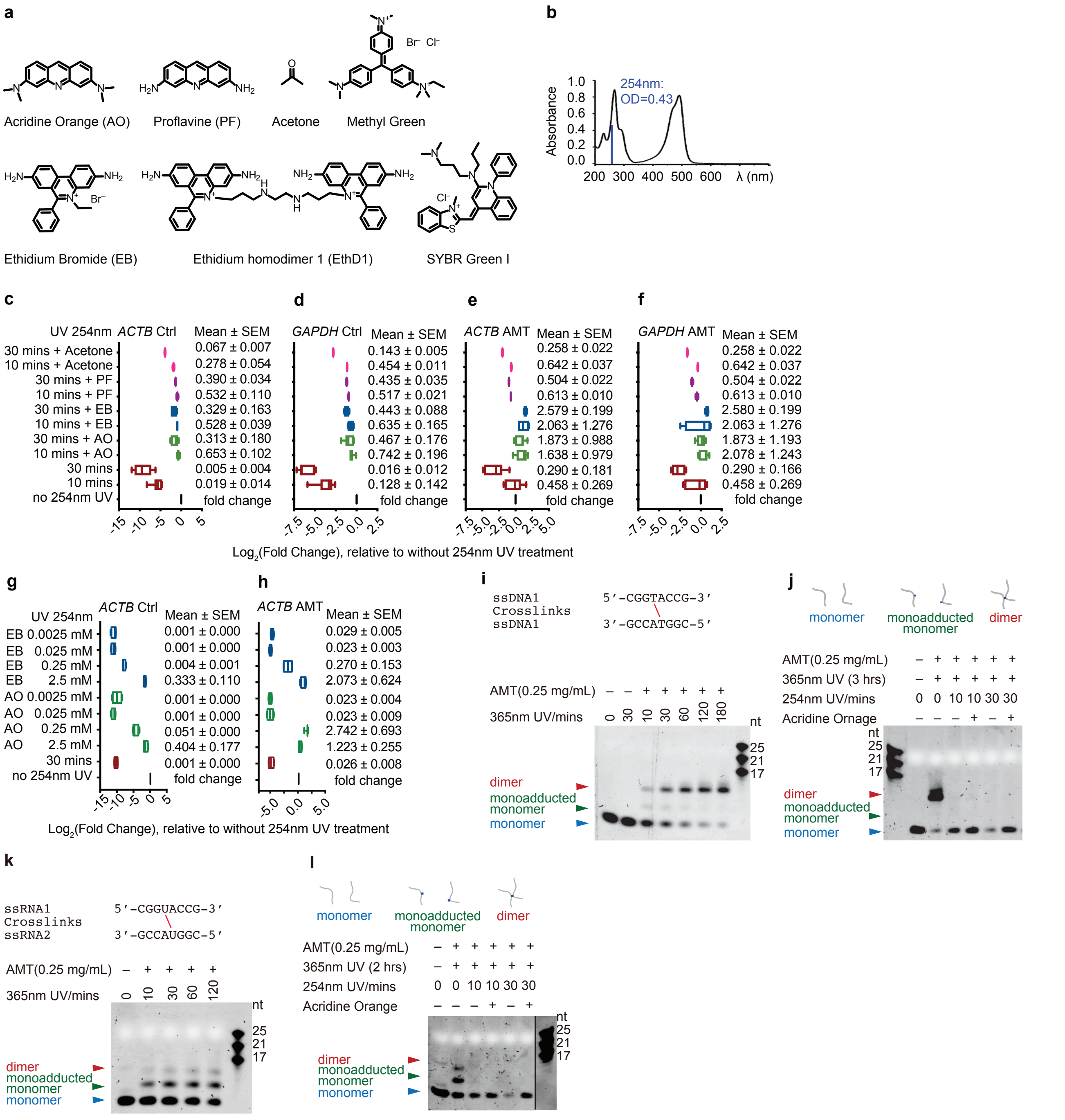

Supplementary Figure 10. Singlet quenchers prevent UVC induced RNA damage without inhibiting reversal of psoralen crosslinks. **a**, Structures of singlet state quenchers used to protect RNA from UVC damages. The structure of SYBR Gold is proprietary so SYBR Green I is shown instead. **b**, UV absorbance of acridine orange; OD=0.43 at 254nm (Selvaggi et al. 2015, Applied Catalysis B: Environmental). **c-f**, cDNA synthesis yield of *ACTB* and *GAPDH* mRNAs with or without different dimer inhibitors during 254nm photo-reversal. Log2-fold change values determined by qPCR and normalized to non-photo-reversal samples. 254nm UV 10 mins and 30 mins were tested. (AO) Acridine Orange, 2.5 mM; (EB) Ethidium Bromide, 2.5 mM; (PF) Proflavine 25 mM; Acetone, 50%. panel **c**: n=12, 12, 12, 8, 12, 4, 4, 2, 2, 2, 2 replicates (from bottom to top); panel **d**: n=11, 10, 11, 6, 10, 4, 4, 2, 2, 2, 2 replicates (from bottom to top); panel **e**: n=11, 9, 11, 8, 11, 4, 4, 2, 2, 2, 2 replicates (from bottom to top); panel **f**: n=10, 10, 10, 7, 8, 4, 4, 2, 2, 2, 2 replicates (from bottom to top); **g-h**, cDNA synthesis yield for *ACTB* mRNA with or without different dose of dimer inhibitors during photo-reversal step. Log2-fold change values determined by qPCR and normalized to non-photo-reversal sample. 254nm UV 30 mins were applied for photo-reversal condition. n=4, biological replicates. **i**, Photo crosslinking of 8-mer DNA oligos at 365 nm UV. Crosslinking times were 10, 30, 60, 120 and 180 mins, respectively. Crosslinked products of 180 mins were used to test photo-reversal condition. **j**, AO did not affect the photo-reversal efficiency of DNA oligo dimers. Crosslinked DNA oligo dimers were reversed by 254 nm UV with or without the protection of AO. The reverse crosslinking times were 10 and 30 mins, respectively. **k**, Photo crosslinking of 8-mer RNA oligos at 365 nm UV. The crosslinking time is 10, 30, 60 and 120 mins, respectively. Most RNA oligos formed monoadducts. Crosslinked products of 120 mins were used to test photo-reversal condition. **l**, AO did not affect the photo-reversal efficiency of RNA oligo dimers and monoadducted monomer. Photo-reversal of crosslink was performed at 254 nm UV. AO was added to inhibit pyrimidine photoproducts. The reverse crosslinking times were 10 and 30 mins, respectively. Box plots in **c-f** and **g-h** show centre line as median, box limits as upper and lower quartiles, whiskers as minimum to maximum values. The experiments in **i, j, k, l** were independently repeated twice with similar results.

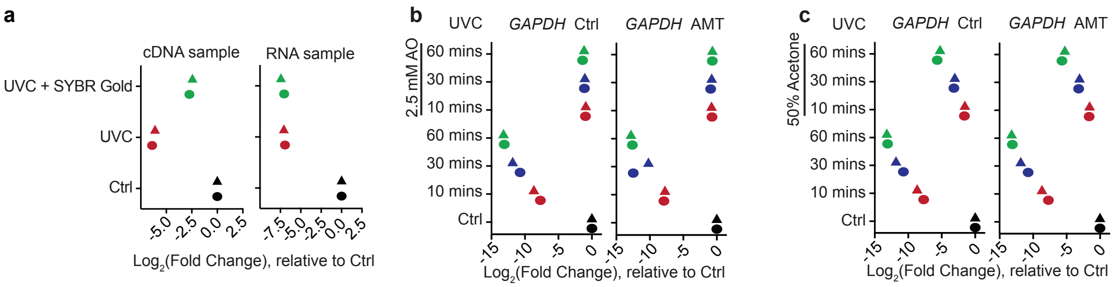

Supplementary Figure 11. Singlet quenchers prevent UVC induced DNA damage. a, SYBR Gold partially protects DNA against UVC damage. UVC damage was quantified by qRT-PCR (*GAPDH*) on the UVC treated cDNA or mRNA. cDNA sample was generated from total RNA of HEK293 cells by SSIII. UVC, 254 nm UV for 30 mins. SYBR Gold can partially block UVC damage for cDNA sample (left side), but not RNA sample (right side). **b**, Acridine orange (AO) prevents DNA sample from UVA damages. 300ng of control and 0.5 mg/ml AMT crosslinked DNA were irradiated by 254 nm UV for 10 mins, 30 mins and 60 mins. 2.5mM acridine orange was used to protect DNA. UVC damages was quantified by qRT-PCR on the *GAPDH* mRNA. Log2-fold change values determined and normalized to control sample. **c**, Acetone partly block UVC damages on DNA sample. 300ng of control and 0.5mg/ml AMT crosslinked DNA were used for testing. 50% of acetone was added to protect DNA sample.

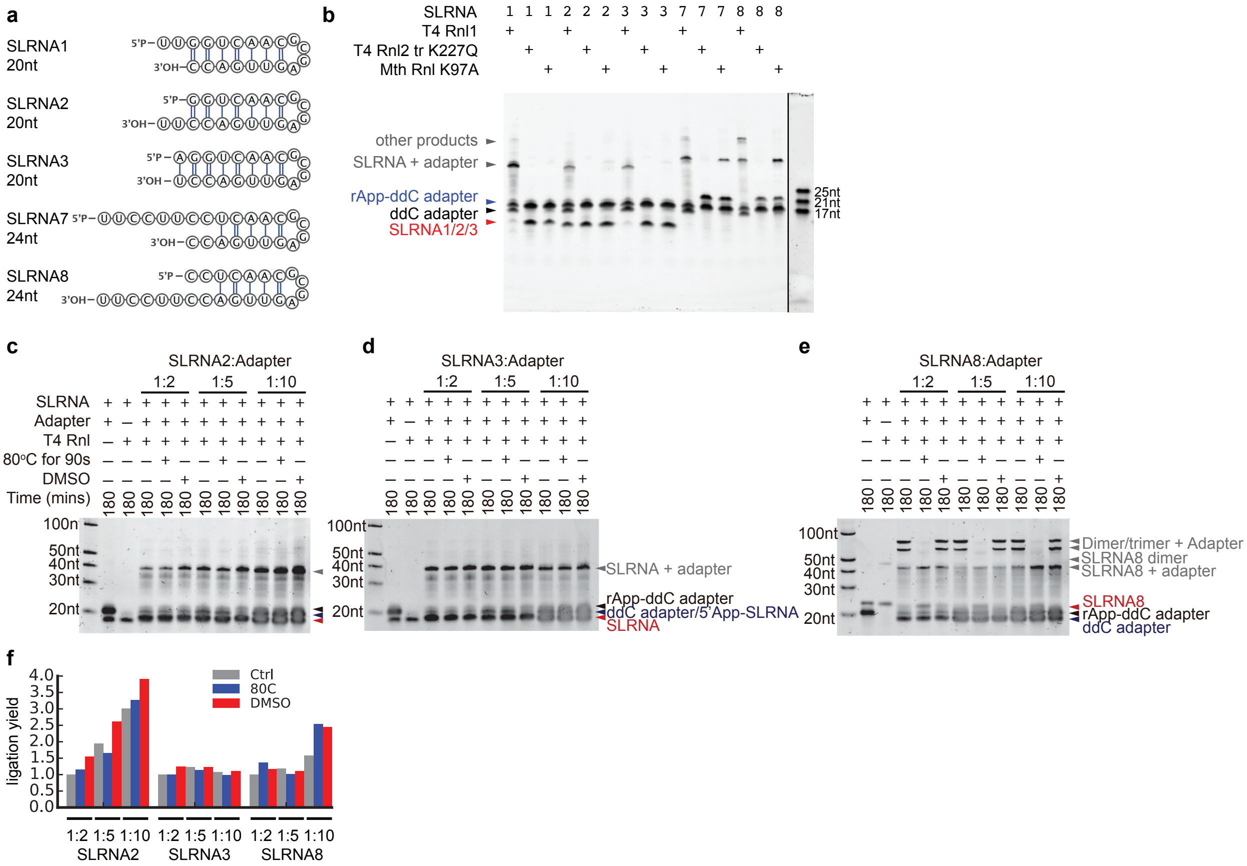

Supplementary Figure 12. Systematic optimization of adapter ligation. **a**, Sequences and secondary structures of synthetic stemloop RNA (SLRNA) oligos used to test ligation conditions. The various stemloop structures were designed to test the efficiency of ligating to structure RNAs. **b**, Electrophoretic gel showing the adapter ligation efficiency of T4 Rnl, T4 Rnl2 tr K227Q and Mth Rnl K97A, respectively. Tested ligation of SLRNA oligos to adenylated ssDNA adapters. **c-e**, Denaturing conditions and abundant adapter increase adapter ligation efficiency. 5pmole SLRNA2/3/8 and 10/25/50 pmole rApp-ddC Adapter were used ligagted by T4 Rnl at room temperature for 3hour. Denaturing treatment: SLRNA 2/3/8 was incubated at 80°C for 90 seconds and snap cooled on ice for at least 1min; DMSO treatment, 10% (v/v) DMSO was added to sample. **f**, Gel quantified ligations efficiency for panels **c-e**. The experiments in **b, c, d, e** were independently repeated twice with similar results.

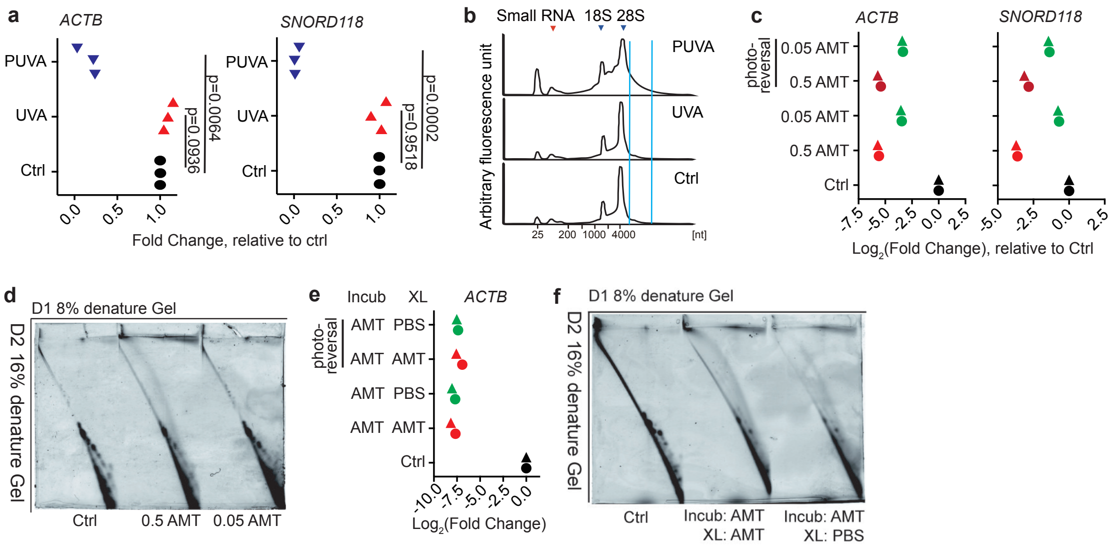

Supplementary Figure 13. PUVA cause RNA damages. a, PUVA, not UVA, will induce RNA damge. cDNA synthesis yield of *ACTB* and *SNORD118* are tested to study the RNA damage. UVA, 365 nm UV irradiated RNA sample for 30 mins; PUVA, AMT 365 nm UV irradiated RNA sample 30 mins with 0.5 mg/ml; n=3, biological replicates. two-tailed, unpaired t-test. **b**, RNA profile after UVA and PUVA treatment. UVA alone did not affect RNA interity. PUVA will induce RNA crosslinking (within blue two lines). **c**, PUVA damage on RNA was related with the concentration of AMT. 0.5 mg/ml and 0.05 mg/ml AMT were used to introduce PUVA damage. The lower concentration of AMT, the less PUVA damages on RNA. PUVA damage still remained after reverse crosslinking by 254 nm UV with the protection of arcidinr orange. cDNA synthesis of *ACTB* and *SNORD118* were used to test PUVA damage. Two replicates were shown for each condition. **d**, 2D gel showing the crosslinking efficiency of different concentration of AMT. Less AMT will reduce the crosslinking efficiency. 0.5mg/ml AMT is necessary for high efficient crosslinking. **e**, Incubation with AMT solution and crosslinking with PBS solution do not reduce PUVA damage. Incubation step (Incub): HEK293 cells were incubated in 0.5mg/ml AMT solution for 15 mins, to make sure AMT penetrate into RNA duplex. Crosslinking step (XL): after incubation, HEK293 cells were irradiated by 365 nm UV for 30 mins in either the same AMT solution or PBS solution. Two replicates wre shown for each condition. **f**, Incubation with AMT solution and crosslinking with PBS solution reduce the crosslinking efficiency. The experiments in **d**, **f** were independently repeated twice with similar results.

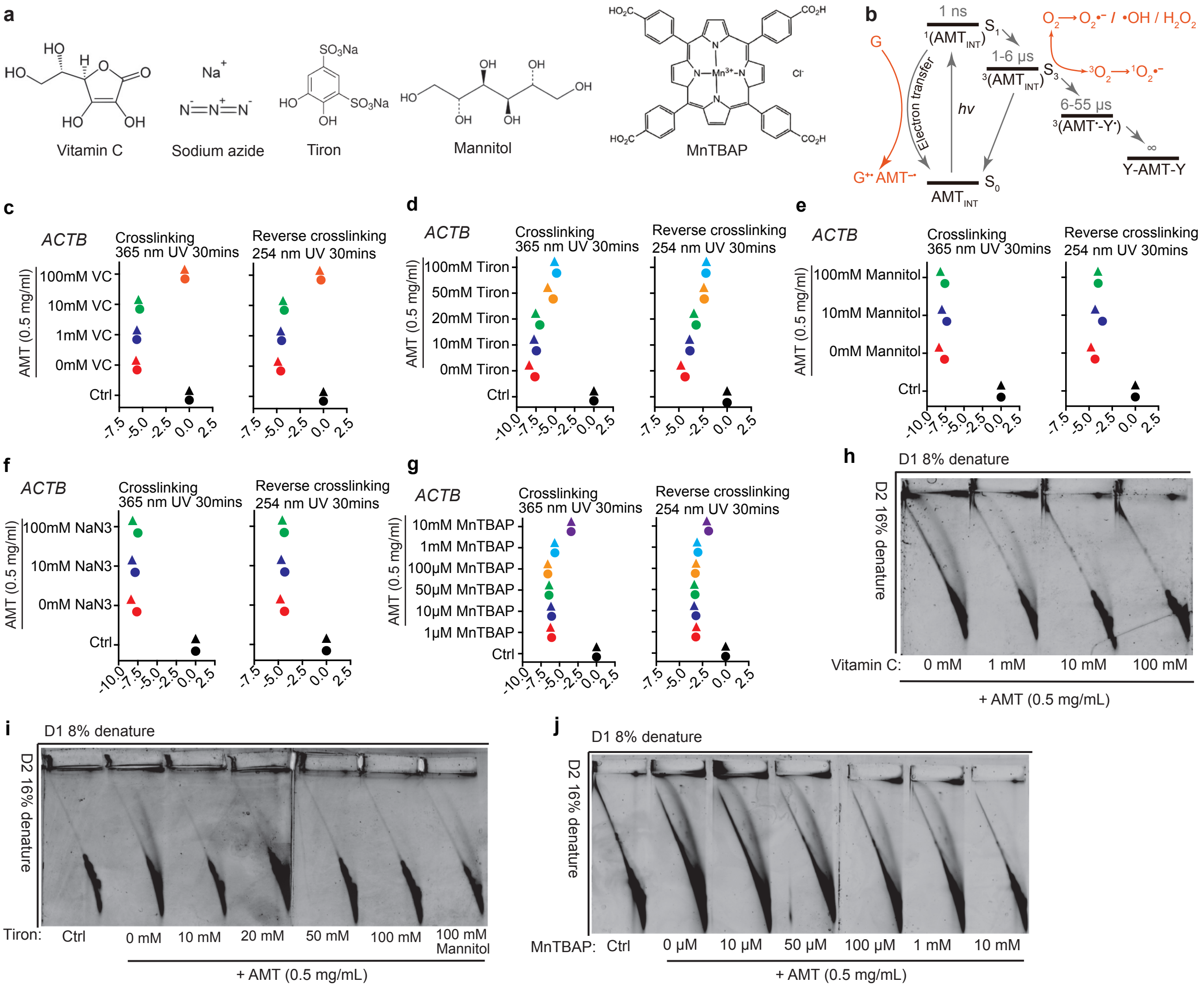

Supplementary Figure 14. Scavengers prevent PUVA damage but also block crosslinking. a, Structures of oxidant scavengers tested in this study. Antioxidants: vitamin C (VC, electron donor); O_2•- scavenger: Tiron and MnTBAP; •OH scavenger: Mannitol; $^1O_2$ scavenger: NaN_3. **b**, An overview of two types of photosensitized RNA damages. Type I, photo-induced electron transfer mechanism. Guanine radical cation forms through the electron transfer reacts with singlet state of AMT, leading to the formation of the oxidized products of guanine. Type II, generation of reactive oxygen species mechanism. Different types of oxygen species are generated from the triplet state AMT. $O_2^{•-}$, superoxide anion radical; •OH, hydroxyl radical; H_2O_2, hydrogen peroxide; $^1O_2$, singlet oxygen. Guanin has lowest oxidation pontential among the four bases are the most frequently oxidized. Less frequent damages, such as hydrates and strand breaks are not shown here. The direct involvement of electron transfer in guanine damage is similar to the pyrimidine crosslinking process, both of which can be quenched by antioxidants. **c-g**, PUVA induced damage in RNA are prevented by some scavengers based on analysis of cDNA synthesis yield from *ACTB* mRNA. Log2-fold change values were determined by qPCR and normalized to control samples. Reverse crosslinked samples by 254nm UV 30 mins are also tested. High concentration of VC (**c**), Tiron (**d**) and MnTBAP (**g**) can reduce PUVA damage. Mannitol (**e**) and NaN_3 (**f**) have no effects on PUVA damage. Two replicates were shown for each condition. **h-j**, 2D gel showing the crosslinking efficiency of HEK293 cells by 0.5 mg/ml AMT in the presence of different scavergers. High concentration of VC (**h**), Tiron (**i**) and MnTBAP (**j**) blocked crosslinking, while mannitol did not (**i**, last panel). The experiments in **h, i, j** were independently repeated twice with similar results.

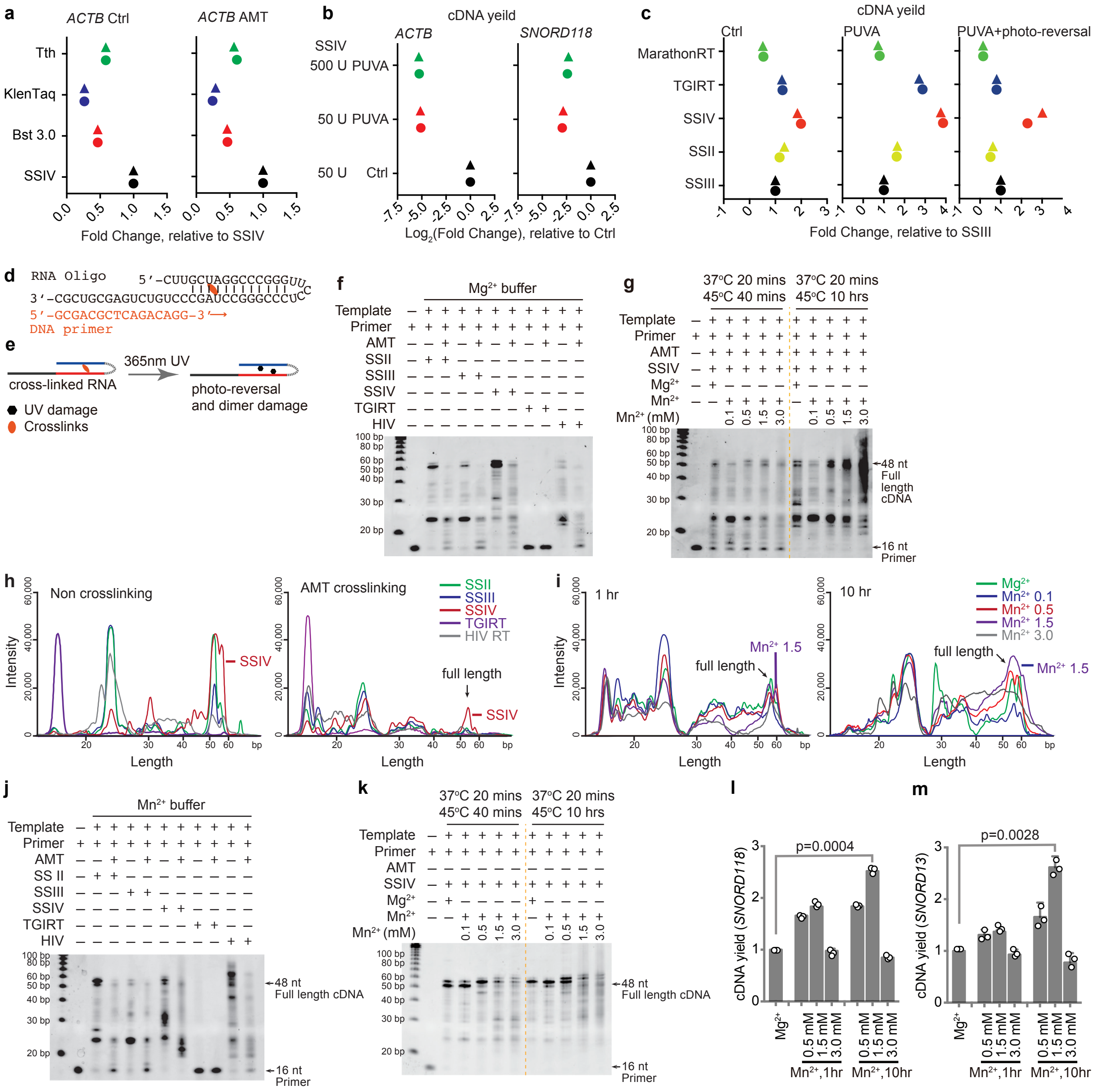

Supplementary Figure 15. Bypass of PUVA induced oxidative damage of RNA. a, DNA polymerases with reverse transcriptase activity cannot bypass PUVA damages. Bypass ability is shown based on cDNA synthesis yield of *ACTB*. Gene specific primer targeting *ACTB* (5'AGCACTGTGTTGGCGTACAG3') was used for reverse transcription. Tth DNA polymerase, KlenTaq DNA polymerase and Bst 3.0 DNA polymerase were tested and compared to SuperScript IV (SSIV). Reverse transcription step was performed according the standard manual of each enzyme. PUVA damage is induced by 0.5mg/ml AMT crosslinking under 365nm UV for 30 mins. Two replicates were shown for each condition. **b**, Reverse transcriptase units with the PUVA damage on RNA. 30ng of control and PUVA damaged RNA was used to test the by pass ability of SuperScript IV (SSIV). Reverse transcription are performed according to standard manual of SSIV. Two replicates are shown for each condition. **c**, The effects of different reverse transcriptases on PUVA damage. cDNA yield of 18S rRNA were tested. PUVA, HEK293 cells were crosslinked by 365 nm UV for 30 mins with 0.5 mg/ml AMT. PUVA+photo-reversal, PUVA sample plus 254 nm UV reversal with the protection of acridine orange. cDNA yield was determined as the Ct value obtained by qPCR of the reverse transcriptional cDNA and normalized to a Superscript III condition. Standard buffers and reaction conditions were used here. Two replicates were shown for each condition. **d,** Sequence of the 48nt RNA oligo template and DNA primer used for primer extension assay. **e**, UVA (365nm) reversal of crosslink simultaneously induce oxidative damages. **f,** Electrophoresis of primer extensions by different reverse transcriptases. Full extension of the primer results in a 48 nt product. Reverse transcriptases used include the Superscript series SSII, SSIII and SSIV, TGIRT-III, and recombinant HIV RT. **g**, The effect of divalent cations and incubation time on full-length cDNA synthesis by SSIV. **h-i**, Quantifying the gel pictures in panels **f**-**g**. The intensity profile for each gel lane was extracted using iBright Analysis Software (v3.1.2). Background was substracted by using a rolling-ball algorithm with 100 μm radius to estimate the amount of background at each position. Pixel positions were converted to DNA length by interpolating the 10bp DNA ladder against pixel position. **j**, Analysis of primer extension products synthesized by different reverse transcriptases in manganese buffer. **k**,The effect of Mg^{2+} and Mn^{2+} buffer on full-length of cDNA synthesis by SSIV. Initial RNA is non-crosslinked. **l-m**, cDNA yield of two snoRNAs *SNORD118* and *SONRD13*, using SSIV in different reaction buffers and different incubation time. Normalized to a standard Mg^{2+} buffer. Data in **l** and **m** are mean ± s.d.; n=3, biological replicates. two-tailed, unpaired t-test. The experiments in **f, g, j, k** were independently repeated twice with similar results.

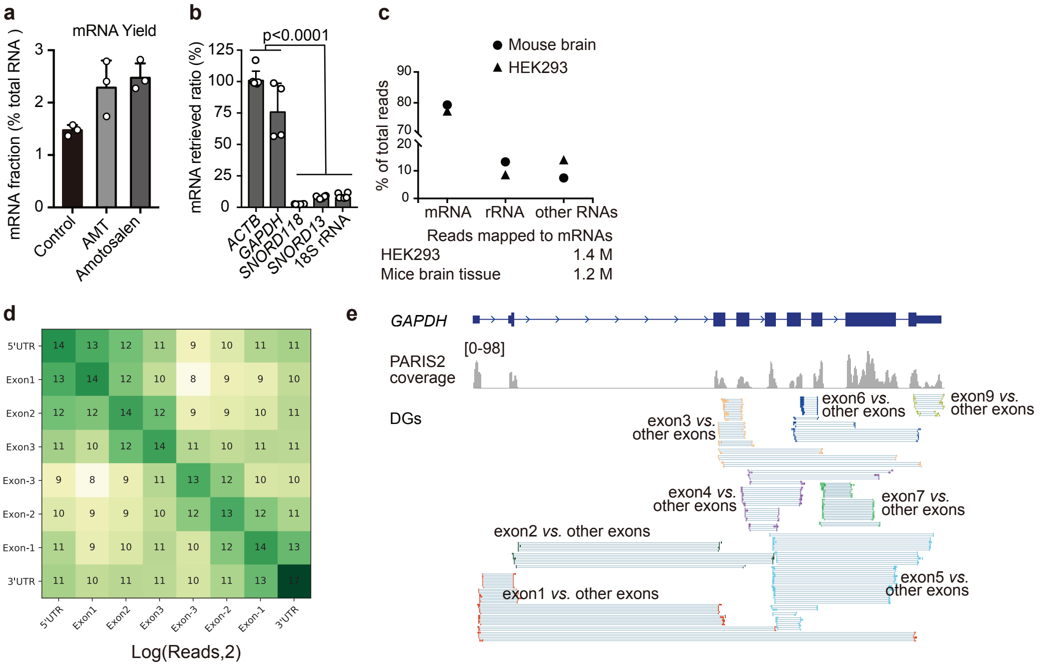

Supplementary Figure 16. PARIS2 analysis of polyA enriched RNAs. a, Enrichment of polyA RNA (including mRNAs and other polyA RNAs) using oligo-dT beads, from control, 0.5mg/mL AMT and 0.5mg/mL Amotosalen crosslinked HEK293 cells. The obvious higher yield from crosslinked samples indicate other RNAs that are covalently linked to polyA RNAs. Data are mean ± s.d.; n=3, biological replicates. **b,** Enrichment of mRNAs relative to noncoding RNAs based on qRT-PCR. Data are mean ± s.d.; *GAPDH*: n=4; others: n=5 biological replicates. two-tailed, unpaired t-test. **c**, Enrichment of mRNAs based on PARIS2 sequencing data from mouse brain and HEK293 cells. Only filtered gapped or chimeric reads were used in the calculation. **d**, Highly structured mRNA. Metagene distribution of PARIS-determined helices among each exons. **e**, PARIS2 showing *GAPDH* mRNA secondary structures in HEK293 polyA enriched RNAs.

a
gencode.v33.annotation.gtf, for hg38, 25 "chr"

Pseudogenes:
IG_C_pseudogene 9
IG_J_pseudogene 3
IG_pseudogene 1
IG_V_pseudogene 188
TR_J_pseudogene 4
TR_V_pseudogene 33
polymorphic_pseudogene 55
processed_pseudogene 10173
pseudogene 18
rRNA_pseudogene 500
transcribed_processed_pseudogene 497
transcribed_unitary_pseudogene 135
transcribed_unprocessed_pseudogene 924
translated_processed_pseudogene 2
translated_unprocessed_pseudogene 1
unitary_pseudogene 97
unprocessed_pseudogene 2629
— Mostly repetitive and masked

Mitochondrial RNA genes:
Mt_rRNA 2, mask and add back
Mt_tRNA 22

Multicopy noncoding genes:
scaRNA 49
snRNA 1901
snoRNA 942
misc_RNA 2212
rRNA 52 (some pseudogenes)

snRNA, mask and add back:
RNU1 (1,11 and 12): 127
RNU2: 71
RNU4 (4 and 4atac): 108
RNU5: 31
RNU6 (6 and 6atac): 1318
RNU7: 149
RNVU1: 24
RN7SK: 1
Others (alphanumeric): 81, mask

repetitive snoRNAs, do not mask:
SNORD113: 9
SNORD114: 30
SNORD115: 48
SNORD116: 30
— special analysis
SNOR, SCA, RNU (no 113-116): 321
Others (alphanumeric): 504, mask

misc_RNA:
RNY: 56, mask, add back
7SK: 292, mask, add back
7SL: 679, mask, add back
VTRNA: 5
Others (alphanumeric): 1189, mask

Protein coding genes:
protein_coding 19957
IG_C_gene 14
IG_D_gene 37
IG_J_gene 18
IG_V_gene 144
TR_C_gene 6
TR_D_gene 4
TR_J_gene 79
TR_V_gene 106

Other RNA genes:
TEC 1060
lncRNA 16892
miRNA 1881
ribozyme 8
sRNA 5 (alphanumeric)
scRNA 1 (BCYRN1)
vaultRNA 1
— Left untreated

ribozyme:
RMRP (1 real and 1 pseudo)
RPPH1 (1 real and 2 pseudo)
Others (alphanumeric): 3

b
hg38_refGene.txt, from IGV, 33478 genes, masking 345

rRNA: RNA5S, 161, RNA5-8, 16, RNA18S, 14, RNA28S, 4, RNA45S, 4.
snRNA: *RNU1*, 40, *RNU2*, 10, *RNU4*, 3, *RNU5*, 5, *RNU6*, 56, *RNU7*, 1, *RNVU1*, 22
Others: *7SK*, 1, *7SL*, 4, *RNY*, 4

c
Dfam RNAs to mask:

5S 1280
7SK 1276
7SL 3007
HY1 462
HY3 572
HY4 174
HY5 25
LSU-rRNA_Cel 131
LSU-rRNA_Hsa 238
SSU-rRNA_Hsa 86
U1 202
U13 484
U14 8
U17 10
U2 830
U3 216
U4 169
U5 176
U6 1789
U7 241

d
Human RNA, 734 from 2450 Rfam families:

miRNAs (mir, let, lin-4): 249. Do not add back
tRNA and tRNA-Sec: 2. Do not add back
SNORA, SNORD, sno, SCAR, ACA, U3, U8: 212
ignore the others
— incomplete compared to genome

RF00030_RNase_MRP (RMRP, 1 real, 1 pseudo in hg38)
RF00009_RNaseP_nuc (RPPH1, 1 real, 2 pseudo in hg38)
RF00024_Telomerase-vert (1 in hg38)

RF00019_Y_RNA
RF00017_Metazoa_SRP (7SL)
RF00100_7SK
RF00001_5S_rRNA
RF00003_U1
RF00004_U2
RF00015_U4
RF00020_U5
RF00026_U6
RF00548_U11
RF00007_U12
RF00618_U4atac
RF00619_U6atac
RF00066_U7

e
Addback (14 "chr"):
hssnRNA (9 together)
RNU7
hs12S
hs16S
hs5S
hs45S (18S, 5.8S, 28S)
RN7SK
RN7SL
RNY (RNY1,3,4,5)
U3
U8
U13
U14AB
U17

f
Before processing:
hg38.fa, 25 chr
gencode.v33.annotation.gtf, 60662 genes,

Processing:
1. Mask pseudogenes and multicopy genes from gencode, refGene and Dfam
2. Add back 14 new "genes", each as a "chromosome"

After masking and adding back single copy genes:
hg38genrefdfamadd.fa, 39 chr
gencode.v33.annotation.gtf, 60662 genes (no need to remove masked)
Plus addback genes.

Supplementary Figure 17. Manually curated human genome reference and annotations. We used the basic hg38 assembly, which contain 25 reference sequences, or "chromosomes", masked the multicopy genes and added back single copies. This reference is best suited for the PARIS analysis. The adjusted genome reference is used for mapping reads and IGV visualization. **a,** Classification and annotation of the Gencode v33 GTF file. Some of the snoRNAs and scaRNAs are repetitive in the genome, but we did not mask them, because there is no easy way to add back a complete set of non-redundant ones. For example, *SNORD3* (10 copies), *SNORD113-SNORD116* all have multiple copies. Several other snoRNAs have fewer copies. snoRNA and scaRNA paralogs can be gathered after mapping to examine interactions. **b**, List of multi-copy genes in the hg38_refGene.txt file from IGV. In the hg38_refGene.txt file, *RNU1* means *RNU1** (including *RNU11*, *RNU12* etc.). **c**, Repetitive RNA genes in Dfam that needs to be masked. **d**, Classification and annotation of the human RNA genes in Rfam. **e**, The list of RNAs to add back to the masked human genome. The 9 snRNAs, U1, U2, U4, U5, U6, U11, U12, U4atac and U6atac are concatenated into one reference, separated by 100nt "N"s. The entire 45S unit is added as one reference. Note. ITS and ETS regions in rRNAs are not masked properly, so reads mapped to these regions should be treated properly. The 4 *RNY* genes are concatenated with 100N spacers. **f,** Pipeline to mask hg38 and add back single copy genes, and summary of the input and output files. Scripts used: maskgencode.py, maskrefgene.py, maskdfam.py.

a
gencode.vM24.annotation.gtf for mm10, 22 "chr"

Pseudogenes:
IG_C_pseudogene 1
IG_D_pseudogene 3
IG_pseudogene 2
IG_V_pseudogene 158
TR_J_pseudogene 10
TR_V_pseudogene 34
polymorphic_pseudogene 88
processed_pseudogene 10002
pseudogene 60
transcribed_processed_pseudogene 300
transcribed_unitary_pseudogene 25
transcribed_unprocessed_pseudogene 272
translated_unprocessed_pseudogene 1
unitary_pseudogene 58
unprocessed_pseudogene 2716
| Mostly repetitive and masked

Mitochondrial RNA genes:
Mt_rRNA 2, mask and add back
Mt_tRNA 22

Multicopy noncoding genes:
scaRNA 51 (no distinct names)
snRNA 1383 (no distinct names) mask and add back
snoRNA 1507 (no distinct names)
misc_RNA 562 (no distinct names)
rRNA 354 (no distinct names) mask and add back

Protein coding genes:
protein_coding 21856
IG_C_gene 13
IG_D_gene 19
IG_J_gene 14
IG_LV_gene 4
IG_V_gene 218
TR_C_gene 8
TR_D_gene 4
TR_J_gene 70
TR_V_gene 144

Other RNA genes:
TEC 3238
lncRNA 9959
miRNA 2202
ribozyme 22
sRNA 2 (alphanumeric)
scRNA 1 (Bc1-ps1)
| Left untreated

ribozyme:
Rmrp (1 real and ?)
RPPH1 (1 real, 3 Rprl and ?)
Others (alphanumeric): 17

b
mm10_refGene.txt, from IGV, 36868 genes

Unlike hg38, many RNA genes are not annotated in mm10_refGene.txt. The 5.8S rRNA locations were extracted from mouse genome+transcripts using BLAST. Among three full length matches, one perfect match is on a 45S sequence two imperfect matches are on assembled chromosomes: chr18:73533406-73533537 (88% identity), chr6:94826786-94826922 (77% identity).

c
Dfam RNAs to mask:

LSU-rRNA_Cel 61
LSU-rRNA_Hsa 182
SSU-rRNA_Cel 1
SSU-rRNA_Hsa 39
U1 320
U13 41
U14 4
U17 21
U2 819
U3 99
U4 97
U5 79
U6 1472
U7 53
U8 6
HY1 43
HY3 14
HY4 5
HY5 1
4.5SRNA 2230
5S 1159
7SK 719
7SLRNA 711
BC1_Mm 31912

d
Mouse RNA, 539 from 2450 Rfam families:

miRNAs (mir, let-7, lin-4): 179. Do not add back
tRNA and tRNA-Sec: 2. Do not add back
SNORA, SNORD, sno, SCAR, ACA, U3, U8: 191
ignore the others (IRES, RNA motifs, etc.)
| incomplete compared to genome

RF00030_RNase_MRP (RMRP)
RF00009_RNaseP_nuc (RPPH1)
RF00024_Telomerase-vert (TERC)

RF00019_Y_RNA
RF00017_Metazoa_SRP (7SL)
RF00100_7SK
RF00001_5S_rRNA
RF00003_U1
RF00004_U2
RF00015_U4
RF00020_U5
RF00026_U6
RF00548_U11
RF00007_U12
RF00618_U4atac
RF00619_U6atac
RF00066_U7

e
Addback 16 "chr":
mmsnRNA (9 together)
RNU7
mm12S
mm16S
mm5S
mm45S (18S, 5.8S, 28S)
RN7SK
RN7SL
RNY (RNY1,3,4,5)
U3
U8
U13
U14
U17
mm4.5S
mmBC1

f
Before processing:
mm10.fa, 22 chr
gencode.vM24.annotation.gtf, 55385 genes

Processing:
1. Mask pseudogenes and multicopy genes from gencode, refGene and Dfam
2. Add back 16 new "genes", each as a "chromosome"

After masking and adding back single copy genes:
mm10genrefdfamadd.fa, 38 chr
gencode.vM24.annotation.masked.gtf, 55385 genes (no need to remove masked)
Plus addback genes

Supplementary Figure 18. Manually curated mouse genome reference and annotations. We used the basic mm10 assembly, which contain 22 reference sequences, or "chromosomes", masked the multicopy genes and added back single copies. This reference is best suited for the PARIS analysis. The adjusted genome reference is used for mapping reads and IGV visualization. **a**, Classification and annotation of the Gencode vM24 GTF file. Some of the snoRNAs and scaRNAs are repetitive in the genome, but we did not mask them, because there is no easy way to add back a complete set of non-redundant ones. For example, *Snord3*, *Snord113-Snord116* all have multiple copies. Several other snoRNAs have fewer copies. snoRNA and scaRNA paralogs can be gathered after mapping to examine interactions. **b**, To maintain consistency with the hg38 genome curation, we also examined the mm10_refGene.txt from IGV, but this annotation missed most of the multicopy RNA genes. **c**, Multicopy RNA genes from Dfam that needs to be masked. **d**, Classification and annotation of the mouse RNA genes in Rfam. **e**, The list of RNAs to add back to the masked mouse genome. The 9 snRNAs, U1, U2, U4, U5, U6, U11, U12, U4atac and U6atac are concatenated into one reference, separated by 100nt "N"s. The entire 45S unit is added as one reference. Note. ITS and ETS regions in rRNAs are not necessarily masked properly, so reads mapped to these regions should be treated properly. **f,** Pipeline to mask hg38pri and add back single copy genes. The 2 RNY genes, they are not all masked in the Gencode list. mm4.5S and mmBC1 are two noncoding RNAs that are not present in the human genome.

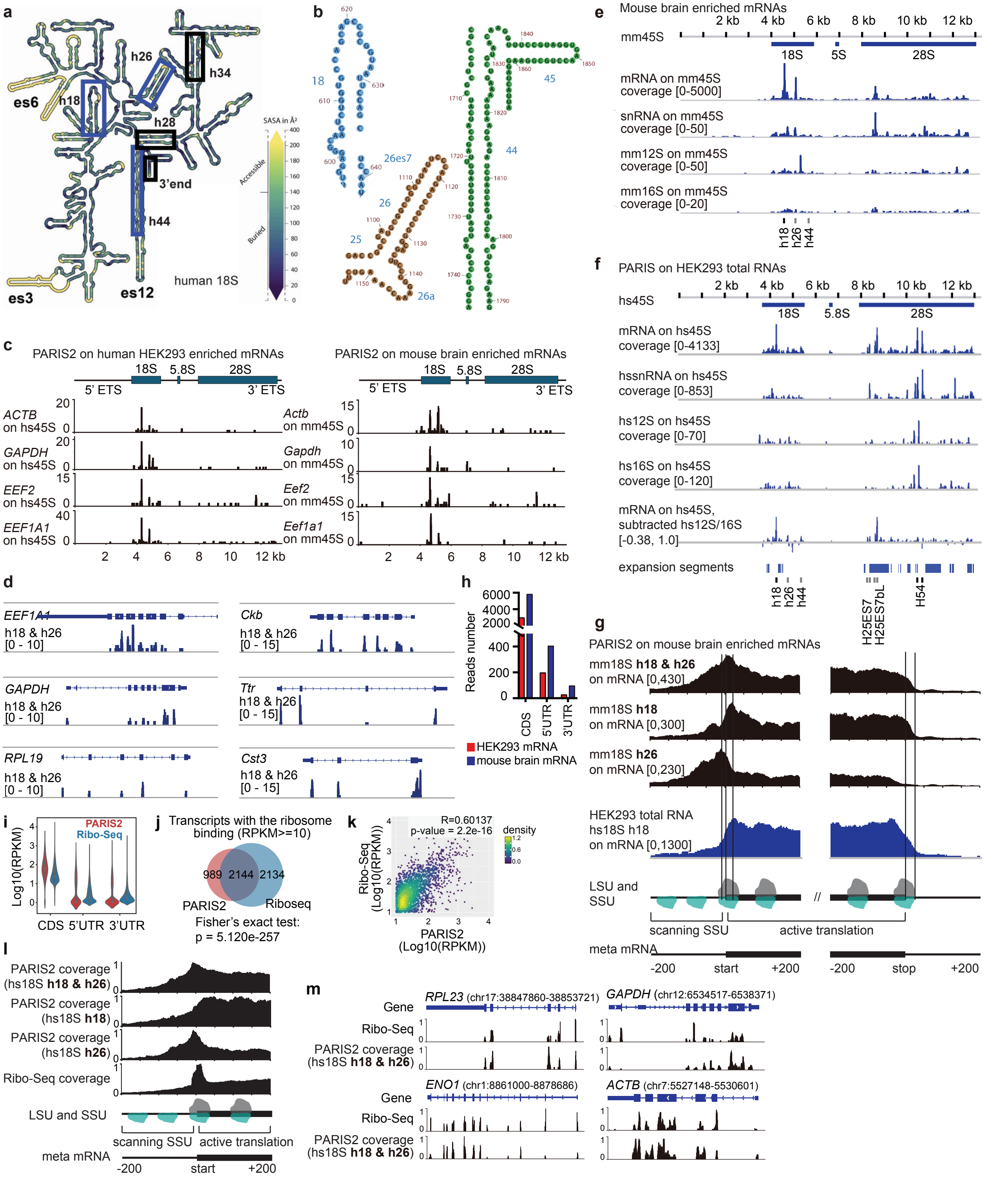

Supplementary Figure 19. PARIS enables ribosome SSU profiling. a, Locations of the mRNA-interacting regions in the 18S ribosomal RNA secondary structure. es3, es6 and es12 are three prominent expansion sequences in 18S. Solvent accessible areas are marked based on the ribosome gallery, from GATech. The blue boxes indicate the three mRNA-interacting 18S regions detected in PARIS. The 3 black boxes highlight additional regions in 18S that can be crosslinked to mRNAs based on Pisarev et al. 2008. **b**, Three18S regions highlighted in blue in panel **a**, including h18, h26, h44-h45 and the 3' end of the transcript. **c**, Examples of mRNA-rRNA interactions in human HEK293 and mouse brain enriched mRNA PARIS2 data. **d**, Examples of mRNAs interact with h18 & h26 domains on 18S rRNA. Left side, HEK293T cells enriched mRNAs; Right side, mouse brain tissues enriched mRNAs. **e**, mRNA binding sites on the mouse 45S rRNA. snRNA, 12S and 16S serve as controls for the specificity. **f**, mRNA binding sites on human 45S rRNA, based on data from PARIS on total RNA in HEK293T cells (Lu et al. 2016 Cell). **g**, SSU h18 and h26 binding sites on mouse and mouse mRNAs. **h**, Comparison of 18S h18 & h26 associated reads number on mRNAs in CDS, 5'UTR and 3'UTR regions. 50nt of 5'UTR and 3'UTR next to CDS were excluded to avoid the extended tails from reads mapped to the CDS. Read numbers were as follows in the order of CDS, 5'UTR and 3'UTR. HEK293: 2958, 196 and 26, mouse brain tissue: 5799, 403 and 95. **i**, Violin plot showing the ribosome binding on CDS, 5'UTR and 3'UTR regions, based on PARIS2 and Ribo-Seq. PARIS2: n=920; Ribo-Seq: n=4274 genes; For the violin and box, the white centre point represents the median, the box limits represent the Q1 and Q3, the whiskers are the most extreme data points within 1.5 × the interquartile range (from Q1 to Q3), and the upper–lower limits represent the maximum–minimum values. **j**, Venn diagram showing overlapped ribosome-associated transcripts between PARIS2 and Ribo-Seq. mRNAs with ribosome binding level (RPKM >= 10) were analyzed here. PARIS2, 0.47million of 18S rRNA chimeric alignments were analyzed here; Ribo-Seq, 4.5 million alignments were analyzed. **k**, Scatterplot comparison of ribosome binding levels on transcripts between PARIS2 and Ribo-Seq. n=2144 genes. P value was calculated using two tailed Fisher's exact test. **l**, Ribosome binding profile around start codons sites on meta mRNAs, with the maximal signal normalized to 1. **m**, Four example mRNAs showing the good correlation between PARIS2 and Ribo-Seq, with maximal signal normalized to 1.

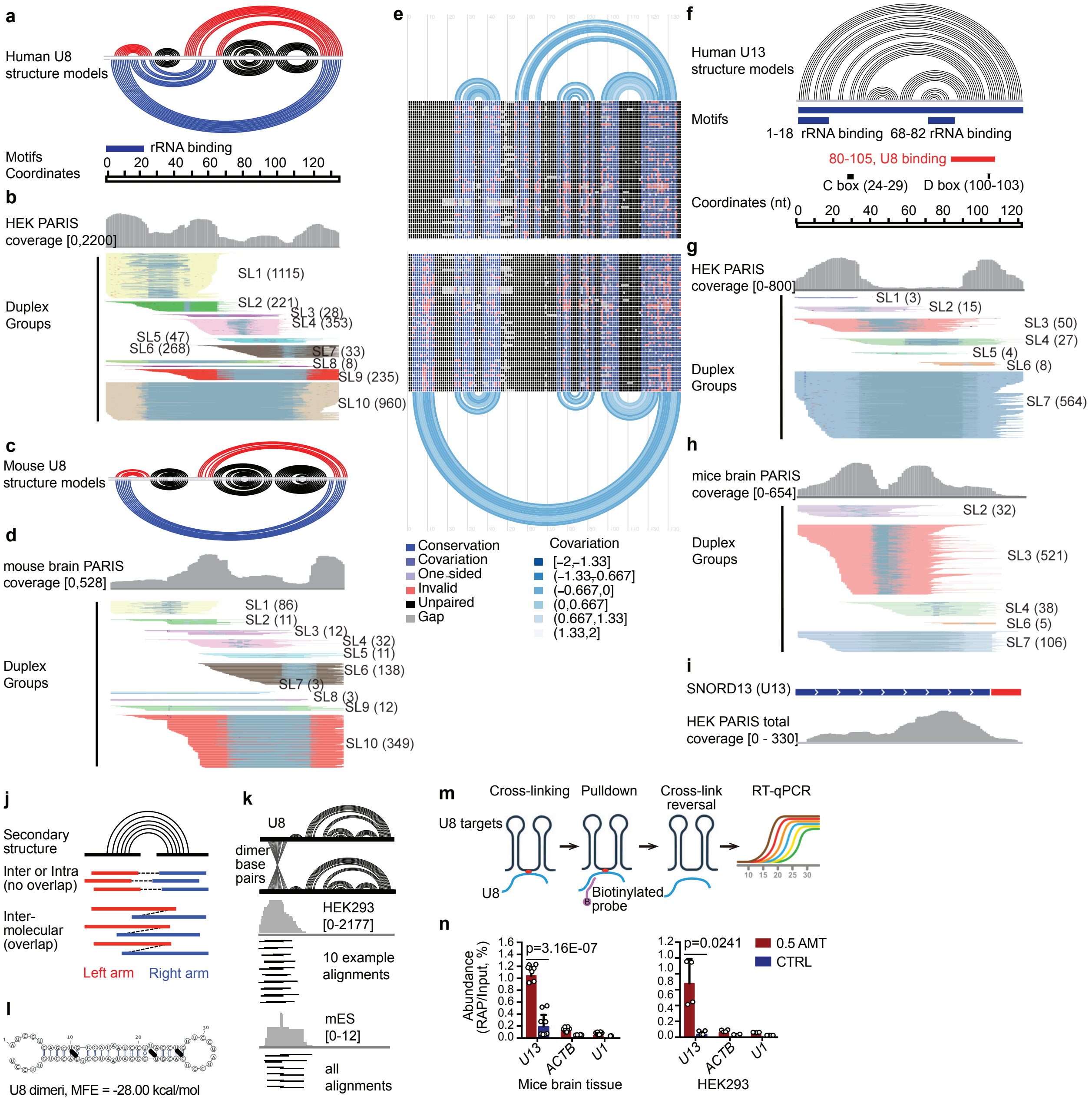

Supplementary Figure 20. Discovery of alternative structures in U8 and U13. a-d, Analysis of U8 structures and interactions in human HEK293 cells and mouse brain. Specific stem loop (SL) structures and their abundances are labeled next to the DGs. **e**, Multiple sequence alignments of U8 homologs confirms 2 alternative U8 intramolecular conformations in all metazoan species that have the U8 gene. **f-h**, Analysis of U13 structures and interactions in human HEK293 cells and mouse brain. The rRNA binding sites are masked in its own duplex regions in both human and mouse cells. These results suggest structural switches in regulating rRNA processing. **i**, The U13 snoRNA gene model was updated based on RNA-seq data from human HEK293 cells. Red bar indicates the new 3' end extension. **j**, Identification of alignments with overlapping segments indicating RNA homodimer. Diagram showing the typical gapped alignments and chimeric alignments where the two arms overlap, indicating RNA homodimers. For small hairpin structures, proximity-ligated fragments with non-overlapping arms may come from either inter or intra molecular duplexes, while overlapping arms can only come from inter-molecular duplexes. Because of this, this analysis is an underestimation of the abundance of inter-molecular duplexes. **k**, Overlapping chimera mapped to the U8 snoRNA in both human and mouse PARIS data. Ten example alignments were illustrated out of 2177 total alignments supporting the homodimer model. **l**, Secondary structure model of the 5' end homodimer region of U8. **m**. Schematic of RNA Antisense Purification combined qPCR (RAP–qPCR). **n**. RAP–qPCR validates *SNORD118* (U8) specific interaction with *SNORD13* (U13). Data are mean ± s.d.; Mice brain: n=8; HEK293: n=4 biological replicates. two-tailed, unpaired t-test.

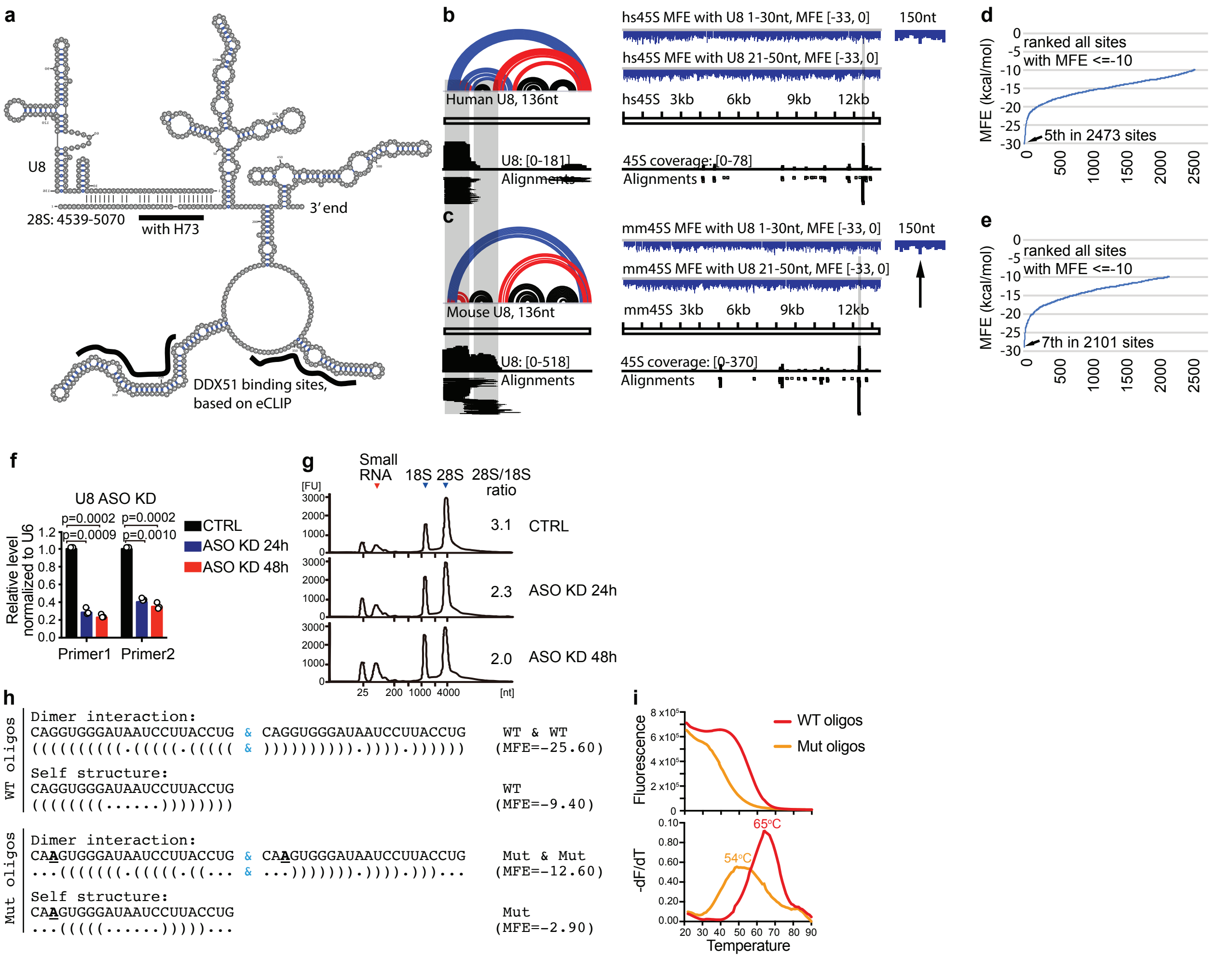

Supplementary Figure 21. Analysis of U8:28S interaction specificity and function. **a**, Mapping the U8:28S interaction site on the 28S secondary structure (4539nt-5070nt, the 3' end of 28S) reveals proximity to the 3' end of 28S, ~18nt away in physical distance, instead of the ~500nt distance on the sequence level. "with H73", a region in 28S that forms helix H73 in the mature ribosome, here alternatively opening up to base pair with U8. Binding sites of DDX51, an RNA helicase previously implicated in rRNA biogenesis are indicated on the 28S rRNA. **b-c**, The U8:28S interaction strength was compared to all possible base pairing sites along the 45S rRNA in both human and mouse. Left panels: alternative secondary structures for human (top) and mouse (bottom) U8. Coverage and alignments supporting the U8:28S interactions were presented below the snoRNA structure models. Middle panels: scanning of the 45S for potential interaction sites (in 30nt windows and 5nt steps). The "MFE with U8" tracks plots were the MFE values for scanning windows on the 45S rRNA with two regions in U8 snoRNA 1-30nt and 21-50nt. A 150nt region surrounding the actual interaction site is shown on the right side for both human and mouse, and the arrows point to the MFE peak for the actual interaction site. The low intensity non-specific interactions between U8 with the rRNA precursor (beyond the true binding site) suggest a scanning mechanism in rRNA binding, where the strongest sites are selected in the end. **d-e**, The PARIS-determined binding sites were among the most stable in all potential binding sites on the 45S rRNA in both human (**d**) and mouse (**e**). **f**. RT-PCR analysis of U8 in control and after ASO knockdown in HEK293 cells. 50nM of ASO were used; Data are mean ± s.d. of n=3 independent experiments. two-tailed, unpaired t-test. **g**, Total RNA profile from control and U8 knockdown HEK293 cells. 28S/18S rRNA ratios were list on the right. **h**, Secondary structure model of the wildtype and LCC patient 3G>A mutant U8 snoRNA. 3G>A mutation reduces secondary structure stability based on minimum free energy (MFE) predictions. **i**, Fluorometric melting curve analysis of secondary structure stability of WT and Mut U8 oligos.

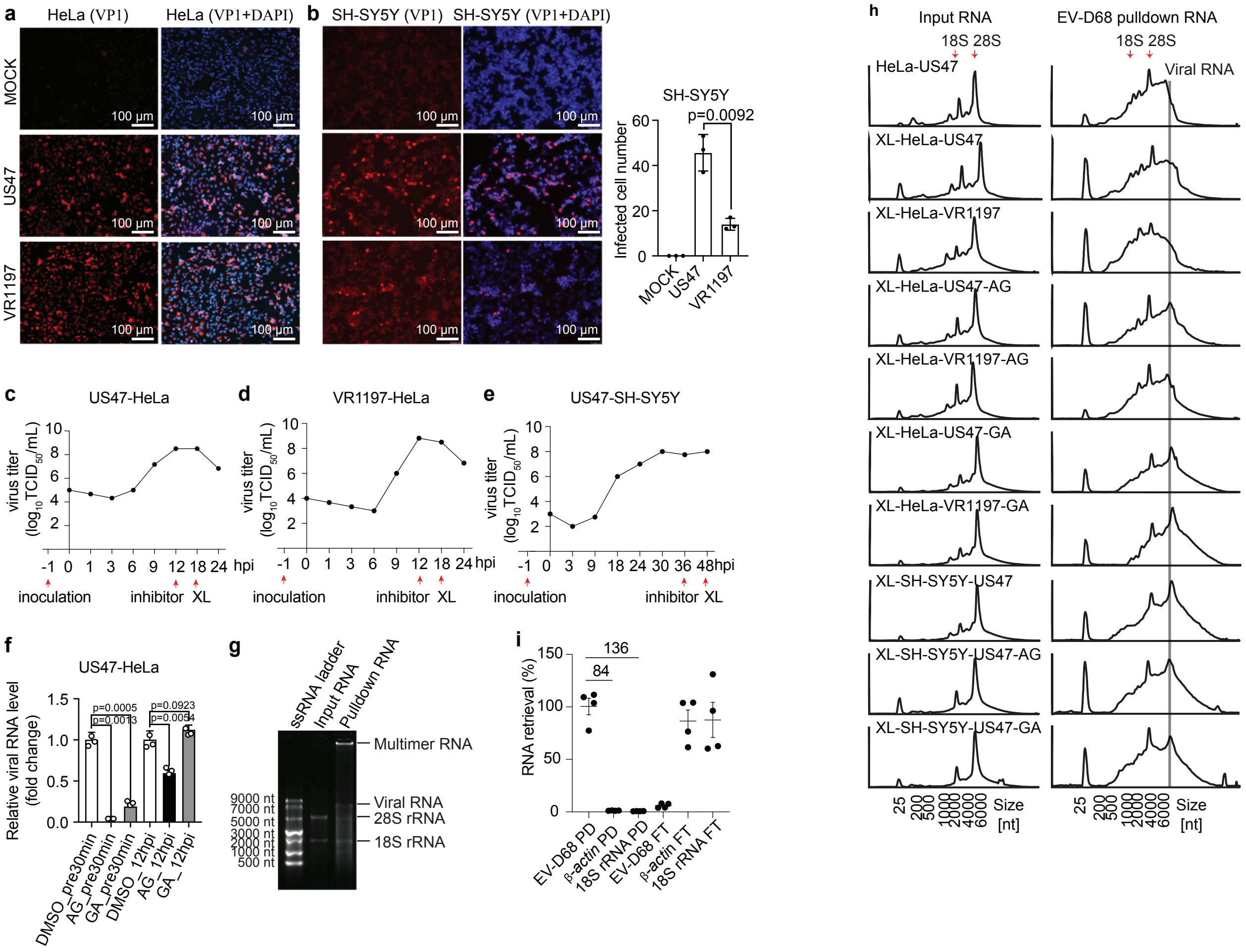

Supplementary Figure 22. Virus culture, enrichment and PARIS2 experiments. a-b, HeLa (**a**) and SH-SY5Y (**b**) cells were infected with US/MO/14-18947 and VR1197 at an MOI of 1.0, Cells were stained with polyclonal antiserum against EV-D68 VP1 (red) and counterstained with DAPI (blue) at 18 hpi (**a**) or 48 hpi (**b**). The bar graph is a comparison of infected cell. Data are mean ± s.d. of n=3 separate fields. two-tailed, unpaired t-test. **c-e**, One-step growth curve of two strains of EV-D68 in HeLa and SH-SY5Y cells. Red arrows were time points of virus inoculation, inhibitor treatment and psoralen crosslinking (XL). **f**, Inhibition efficiency of AG/GA treatment of HeLa cells before (pre30min: 30 min pre inoculation) and after (12hpi: 12 hours post infection) US47 infection. Primers targeting 5'UTR of US47 were used. Pretreatment with inhibitors almost completely blocked virus replication, making it impossible to analyze the RNA structures, therefore we focused on the post-infection treatments. Data are mean ± s.d. of n = 3 independent experiments. two-tailed, unpaired t-test. **g**, Agarose gel RNA profiles of EV-D68 pulldown or input RNA from cells infected with EV-D68. **h**, Input and EV-D68 RNA pulldown profiles based on TapeStation analysis (retraced and aligned based on the agarose gel electrophoresis). **i**, Enrichment of viral genome RNA and depletion of host RNAs based on qRT-PCR. PD: pulldown, FT: flow-through. Viral RNA primers and two sets of control primers for *beta-actin* and 18S rRNA, respectively, were used to amplify EV-D68 PD and FT samples. The enrichment folds of viral RNA versus both controls are labeled above the black lines. Data are mean ± s.d. of n=4 replicates. The experiments in **a** and **g** were independently repeated twice with similar results.

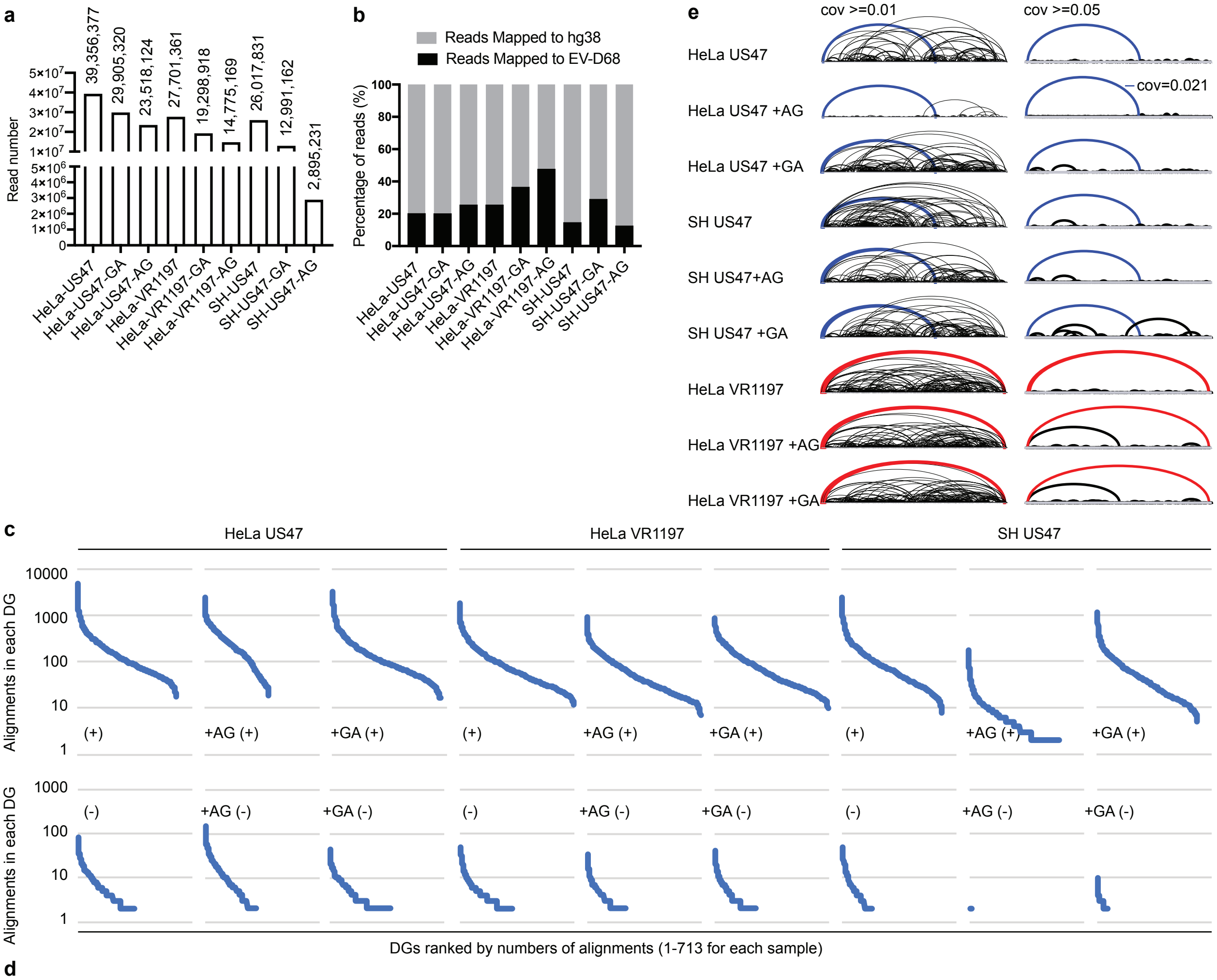

d

PARIS2 Samples	All DGs	All pri_gap1	All DG max	cov 0.01 DGs	cov 0.01 pri_gap1	cov 0.01 DG min	cov 0.1 DGs	cov 0.1 pri_gap1	cov 0.1 DG min
HeLa US47 (+)	12925	193484	4652	618	120123	18	53	42321	196
HeLa US47 +AG (+)	5289	124678	2368	402	105935	19	138	71151	130
HeLa US47 +GA (+)	9496	178511	3275	676	122606	17	69	50888	131
HeLa VR1197 (+)	11073	127103	1826	714	78632	12	65	28759	90
HeLa VR1197 +AG (+)	5665	69772	902	712	50395	7	77	17918	17
HeLa VR1197 +GA (+)	6881	95318	837	711	67174	10	66	22662	96
SH US47 (+)	6337	101665	2416	629	75344	8	86	33859	85
SH US47 +AG (+)	578	4457	176	567	4435	2	137	2585	3
SH US47 +GA (+)	3702	56918	1166	629	45797	28	101	23690	44
HeLa US47 (-)	382	2883	82	355	2827	2	36	990	9
HeLa US47 +AG (-)	351	4280	149	323	4223	2	86	2702	7
HeLa US47 +GA (-)	382	1995	45	378	1987	2	43	640	6
HeLa VR1197 (-)	344	1987	48	325	1948	2	21	466	5
HeLa VR1197 +AG (-)	237	1105	33	237	1105	2	42	444	2
HeLa VR1197 +GA (-)	256	1333	42	256	1333	2	51	606	3
SH US47 (-)	189	1322	48	187	1318	2	52	738	2
SH US47 +AG (-)	4	8	2	4	8	2	4	8	2
SH US47 +GA (-)	66	198	10	66	198	2	57	176	2

Supplementary Figure 23. Summary of EV-D68 PARIS2 data and DG assembly for all 9 samples. a, Total reads for each condition. **b**, Percentage of reads mapped to the EV-D68 genome and human genome (hg38). Analyzed reads number (n) of each condition was listed in panel **a**. **c,** DGs assembled by CRSSANT for both strands (+ and -) were ranked by numbers of alignments in each DG and plotted in log scale. The minus strands (bottom panels) have fewer DGs due to the lower coverage. **d**, Numbers of DGs and alignments for all datasets. All DGs: Numbers of all duplex groups. All pri_gap1: total numbers of alignments for all DGs. All DG max: numbers of alignments for the biggest DG. cov 0.01 DGs: numbers of DGs where the minimal cov >=0.01 (cov defined as gapped alignments divided by the square root of the product of coverages at the two arms). cov 0.01 pri_gap1: total numbers of alignments with cov >=0.01. cov 0.01 DG min: the smallest DG with the cov >=0.01. Similarly, the numbers were calculated for DGs with a minimum cov >=0.1. The numbers were calculated for both strands. **e**, Visualization of DGs with relative coverage cutoff (cov) at 0.01 and 0.05 for all 9 samples on the (+) strand. The blue and red arcs highlight the key different long-range structures between US47 and VR1197 strains, which is more prominent at cov>=0.05 (with the exception of the HeLa US47+AG sample, where the long-range arc has a cov=0.021, between 0.01 and 0.05). This analysis shows that there are no clear large scale domains. This is in contrast to lncRNAs like *XIST*, *MALAT1* and *NEAT1*, which have strong and conserved large scale domains (see our previous studies Lu et al. 2016 Cell, Lu et al. 2020 Nature Communications).

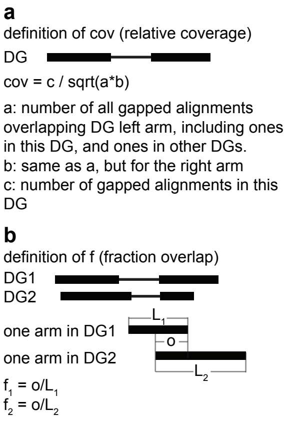

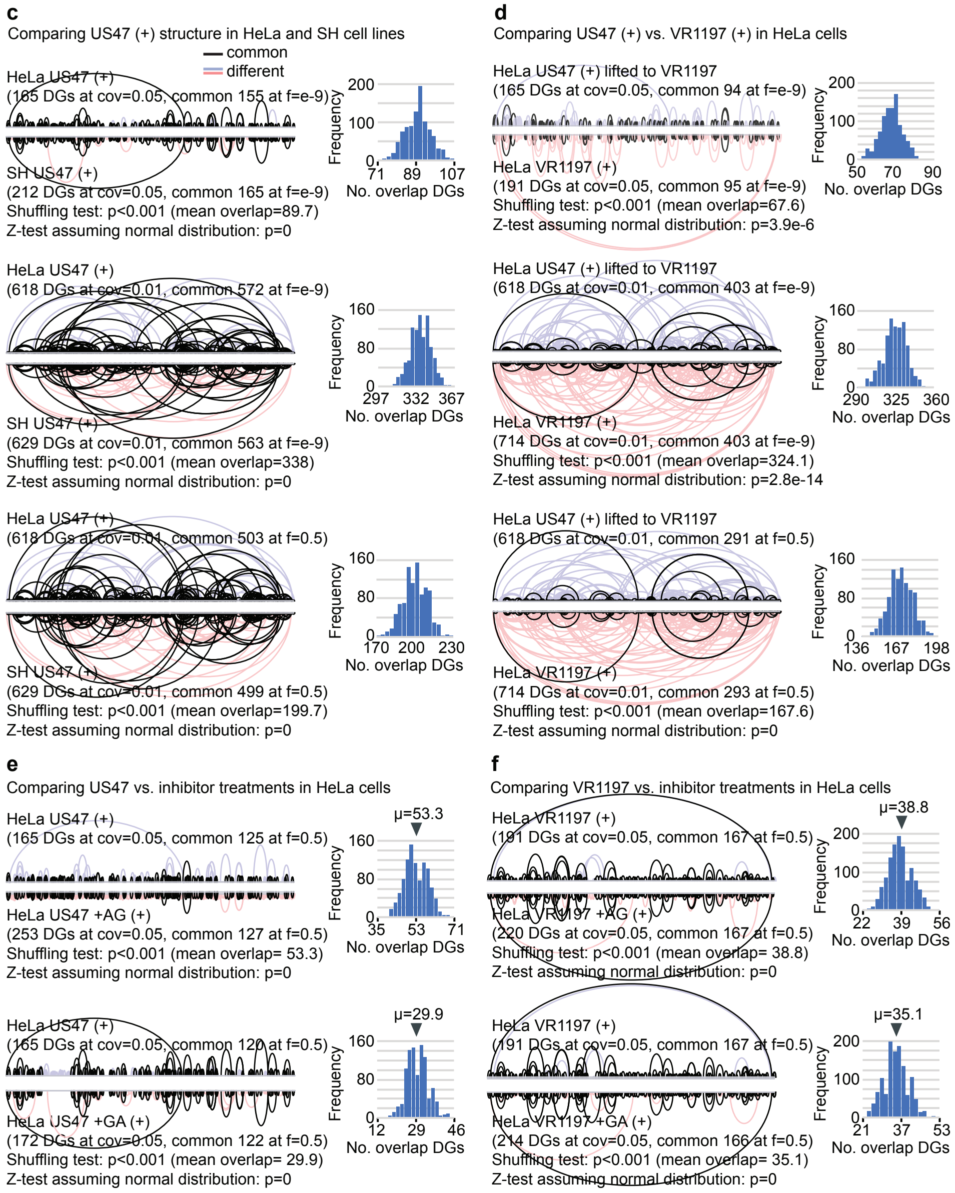

Supplementary Figure 24. Pair-wise comparisons of DGs among different conditions. **a**, Definition of relative coverage (cov), as described in Lu et al. 2016 Cell. **b**, Definition of fraction overlap is the same as in the Bedtools package. In the current analysis, we require reciprocal overlap, i.e. both overlap f_1 and f_2 are higher than the threshold, for both arms of DG1 and DG2. **c**, Comparing US47 (+) strand RNA structures in HeLa vs. SH-SY5Y cells. Black arcs: structures that are shared between the two conditions. Blue and red arcs: structures are are unique in either conditions. Overlaps were calculated at the relative coverage (cov) of 0.01 and 0.05, and fraction overlap (f) of 10^{-9} (effectively 1nt for each arm) and 0.5 (50% for each arm). Positions of the DGs were shuffled 1000 times along the genome. Overlaps were calculated and plotted in a histogram (on the right). The mean overlap is the average numbers of overlapped DGs in the 1000 shuffles. Significance of overlap (P value) was calculated by comparing the number of overlapped DGs to the shuffled distribution, or using a Z-test assuming normal distribution of the shuffled results. **d**, Comparing US47 and VR1197 (+) strand structures in HeLa cells at various cov and f thresholds. Local structures are more conserved than the long-range ones. **e-f**, Comparing structures in normal and inhibitor treated conditions for the 2 strains.

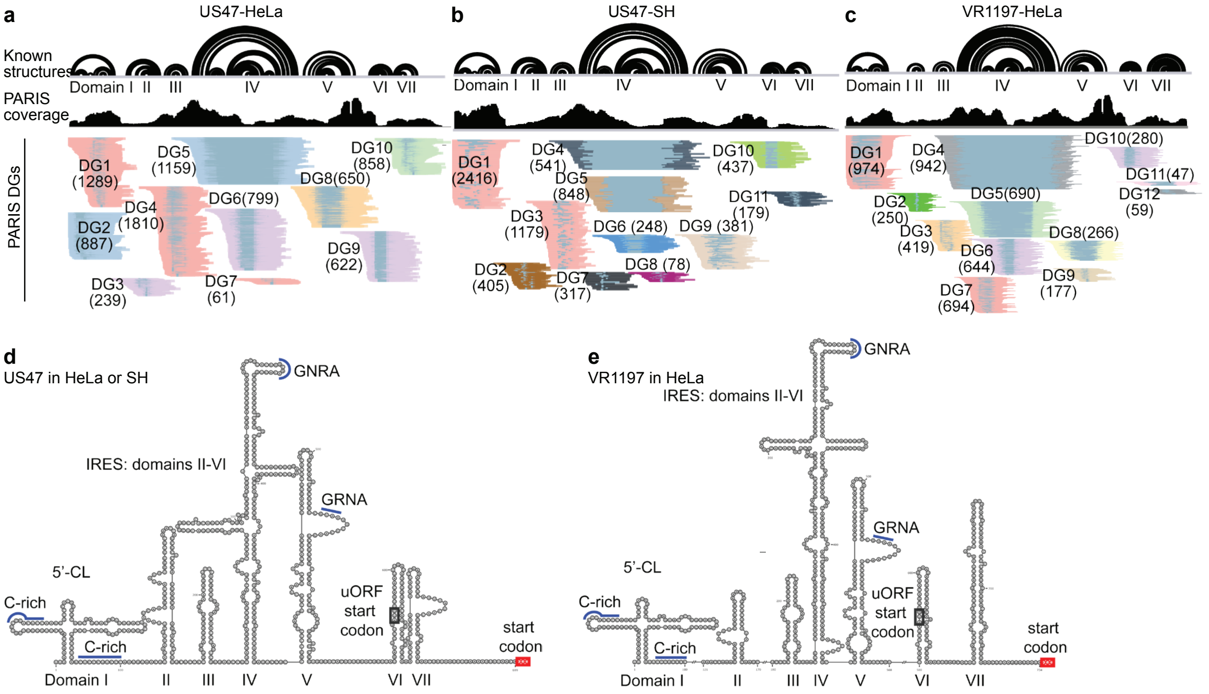

Supplementary Figure 25. PARIS2 confirms known structures in 5'UTR of EV-D68 in cells. a-c, PARIS2 data, duplex groups, and structure models for the 5'-cloverleaf (5'-CL) and IRES element in 5'UTR of US47 and VR1197 in HeLa and SH-SY5Y cells. The numbers of alignments in each DG are indicated in parentheses. **d-e**, Stemloop structure model of the 5'UTR. The IRES structure model consists of five domains designated II-VI. Several motifs within the IRES, two GNRA tetraloops in domains IV and V and a pyrimidine-rich track (Yn) between domains V and VI, are essential for the recruitment of translation initiation factors. These motifs are clearly identified on the PARIS2-derived structure models.

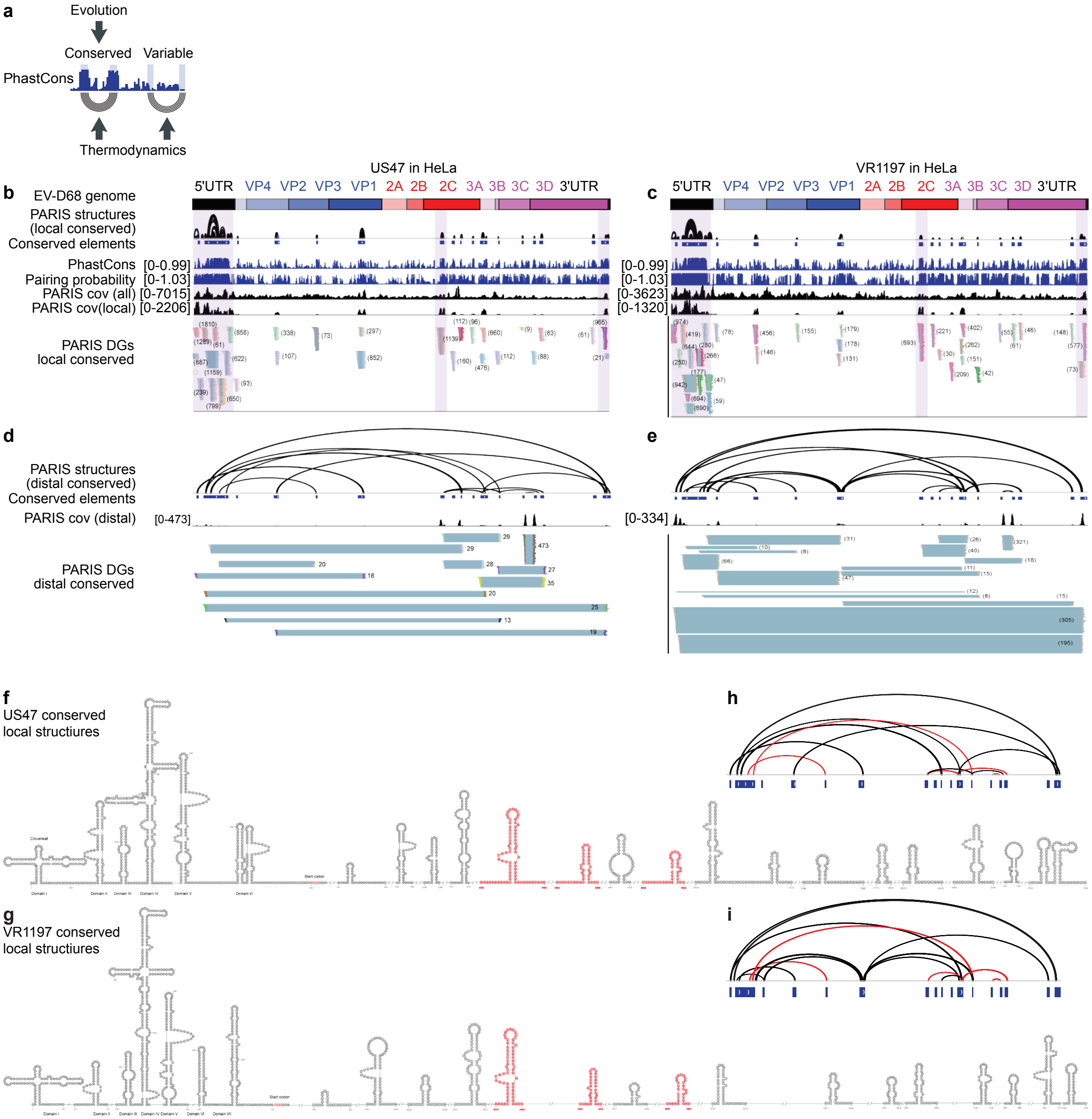

Supplementary Figure 26. **Global conserved local structures and long-range structures in EVD68 VR1197 genome supported by PARIS2 data. a**, Thermodynamics is the primary force underlying RNA structure formation, whereas a subset of these structures are constrained by evolution. The phastcons track is an example showing the conservation on the sequence level. **b-c**, Local structures overlapping conserved sequence elements in US47 and VR1197 in HeLa cells. Phastcons, conserved element and pairing probability tracks were EV-D68 multiple sequence alignments. In the DG track, numbers of alignments for each DG are included in paretheses. The 3 shaded area are the 5'UTR, the 2C-CRE and the 3'UTR plus nearby coding region. **d-e**, Long-range DGs that overlap the conserved sequence elements in US47 and VR1197. **f-g**, Secondary structure models for the DGs overlapping conserved sequence elements in the two strains. The red stemloops are structures in the coding regions that are shared between the two strains. **h-i**, Comparing US47 and VR1197 long-range structures that overlap the conserved sequence alignments. Highlighted in red are common ones between US47 and VR1197.

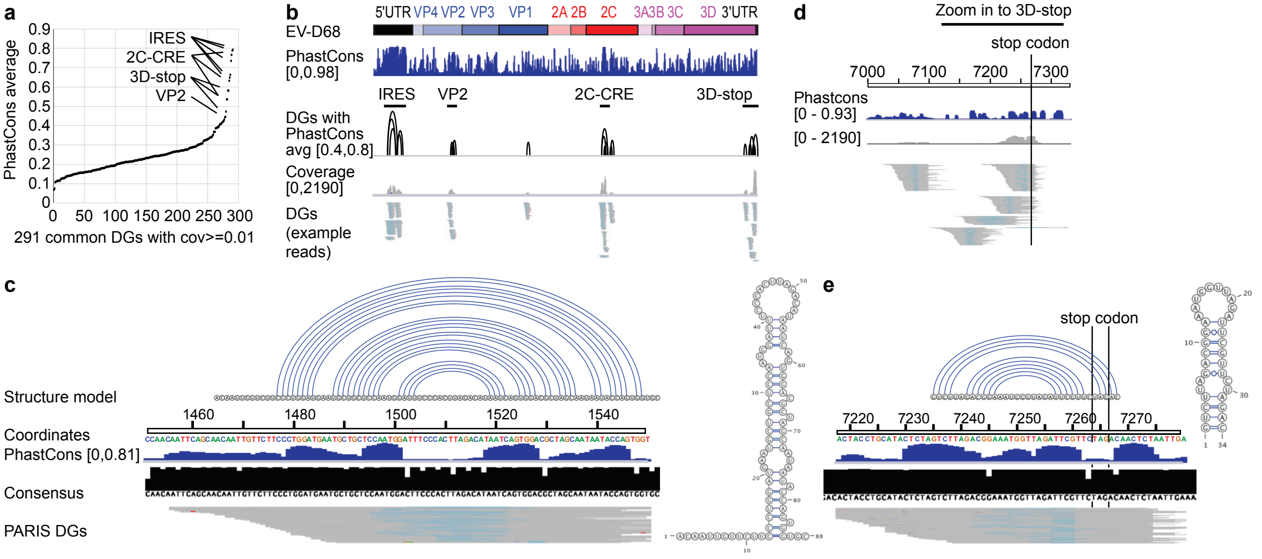

Supplementary Figure 27. Analysis of structures in the highly conserved regions in the EV-D68 genome. a, All common DGs were ranked based on the Phastcons average value. Structures in the IRES, 2C-CRE, the 3Dstop region and VP2 were among the most highly conserved. **b**, Locations and DGs for the most conserved structures. **c**, Structure in the VP2 region, showing the structure model, Phastcons, consensus sequence from 492 complete genomes and the PARIS DG. The stemloop model was shown on the right. **d**, DGs near the 3D stop codon. **e**, Structure of the DG spanning the stop codon.

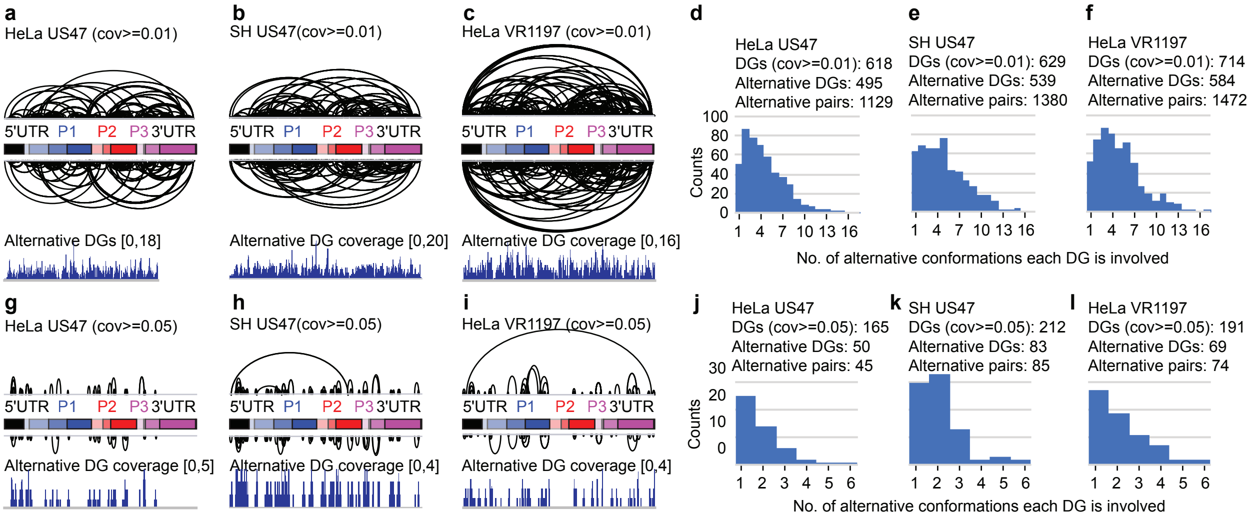

Supplementary Figure 28. Analysis of alternative conformations on the (+) strand RNAs. a-c and **g-i,** DGs that form alternative conformations, and their coverage along the genome. In each pair of alternative conformations, one DG was plotted as an arc above the genome annotation, while the other was plotted below. **d-f** and **j-l,** numbers of alternative conformations that each DG was involved in. **a-f**, DGs at with a coverage >=0.01. **g-l**, DGs at with a coverage >=0.05. This analysis showed that alternative conformations were prevalent along the entire genome. About 80% of all DGs (cov >=0.01) were involved in alternative conformations. About 30-40% of all DGs (cov >=0.05) were involved in alternative conformations.

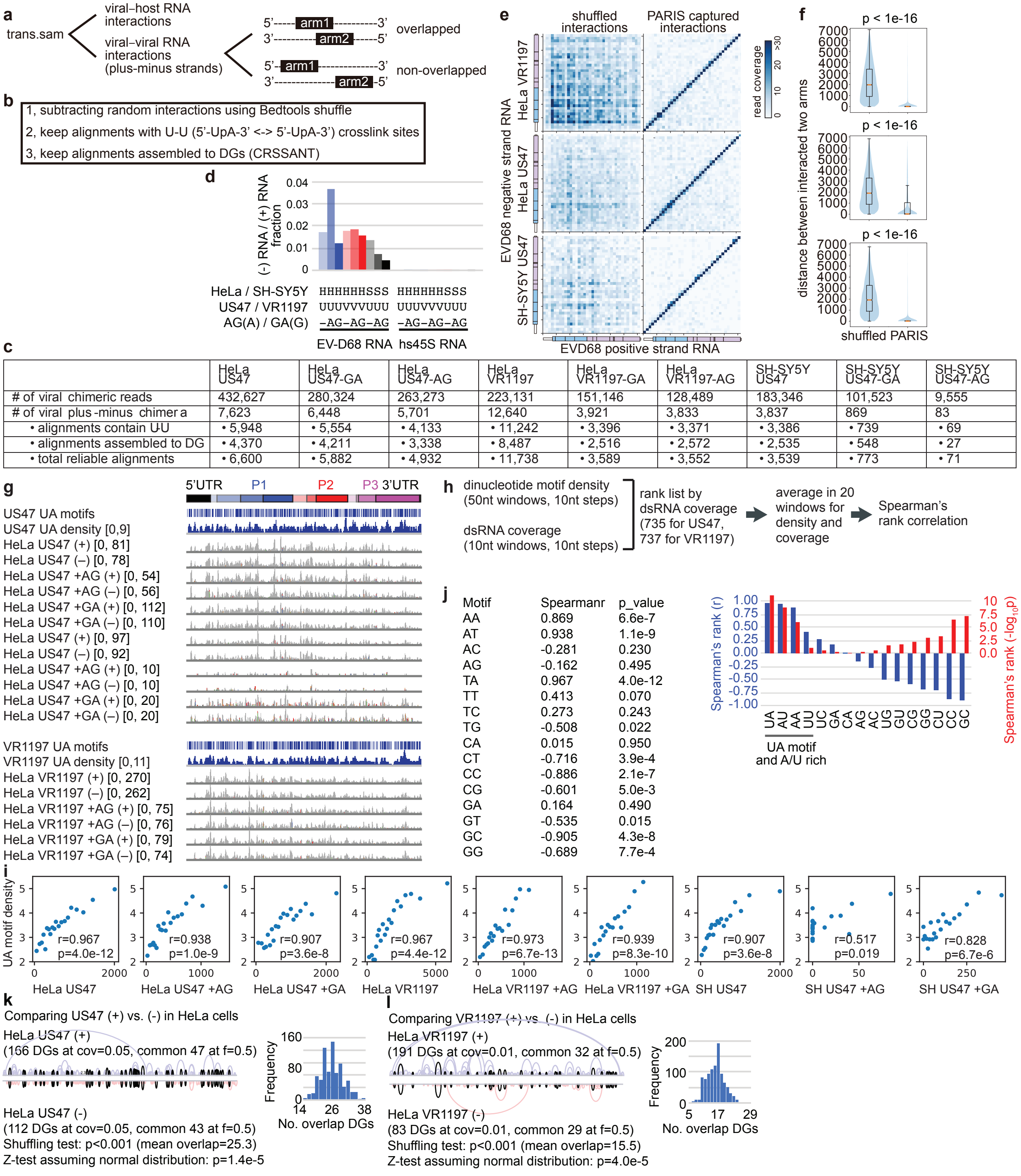

c

	HeLa US47	HeLa US47-GA	HeLa US47-AG	HeLa VR1197	HeLa VR1197-GA	HeLa VR1197-AG	SH-SY5Y US47	SH-SY5Y US47-GA	SH-SY5Y US47-AG
# of viral chimeric reads	432,627	280,324	263,273	223,131	151,146	128,489	183,346	101,523	9,555
# of viral plus-minus chimera	7,623	6,448	5,701	12,640	3,921	3,833	3,837	869	83
• alignments contain U-U	• 5,948	• 5,554	• 4,133	• 11,242	• 3,396	• 3,371	• 3,386	• 739	• 69
• alignments assembled to DG	• 4,370	• 4,211	• 3,338	• 8,487	• 2,516	• 2,572	• 2,535	• 548	• 27
• total reliable alignments	• 6,600	• 5,882	• 4,932	• 11,738	• 3,589	• 3,552	• 3,539	• 773	• 71

j

Motif	Spearmanr	p_value
AA	0.869	6.6e-7
AT	0.938	1.1e-9
AC	-0.281	0.230
AG	-0.162	0.495
TA	0.967	4.0e-12
TT	0.413	0.070
TC	0.273	0.243
TG	-0.508	0.022
CA	0.015	0.950
CT	-0.716	3.9e-4
CC	-0.886	2.1e-7
CG	-0.601	5.0e-3
GA	0.164	0.490
GT	-0.535	0.015
GC	-0.905	4.3e-8
GG	-0.689	7.7e-4

Supplementary Figure 29. PARIS2 detects interactions between (+) and (-) RNA strands. a, Diagram for the analysis strategy. **b**, Analysis steps for counting alignments. **c**, Numbers of alignments that fall into each of the categories described in panel (**b**). **d**, Specificity and sensitivity of (-) RNA recovery, based on the detection of (-) RNA for EV-D68 but not ribosomal RNAs which are highly abundant. On average, the relative amount of (-) EV-D68 RNA was about 400-700 fold of hs45S (barely detectable), suggesting that the (-) RNA is not library prep artifact. Analyzed reads number (n) of each condition was listed in **supplementary figure 23a**. **e**, Heatmap of the PARIS2 coverage for EVD68 plus-minus strand RNA chimera. Shuffled interactions served as negative control. **f**, Violin plot for the distributions of distance between the two arms. HeLa-US47: n=6600; HeLa-VR1197: n=11738; SHSY5Y-US47: n=3539 of alignments. For the violin and box, the pink line represents the median, the box limits represent the 1st and 3rd quartiles, and the upper–lower limits represent the maximum–minimum values. p-values of two-sided Wilcoxon rank-sum tests were displayed. **g**, Comparing the UA dinucleotide motif density to the coverage of chimera from PARIS2 where the two arms overlap. The UA dinucleotide motif density was calculated in 50nt windows and 10nt steps. **h-i**, Spearman's rank correlation between the UA motif density and the coverage of chimera. n=20 for each plot. p-values are computed using algorithm AS 89. Note, the lower correlation for SH US47 +AG is due to the extremely low coverage. **j**, Spearman's rank correlation of the overlapped chimera coverage in the US47-HeLa sample to all 16 possible dinucleotide motifs and the significance. The bargraph shows the sorted correlations, highlighting the strongest correlation to UA and A/U rich sequences. This analysis confirmed the bias in the UpA crosslinking by psoralens, and also showed that the good coverage of the entire length of the RNA. n=735 of rank list by dsRNA coverage. p-values are computed using algorithm AS 89. **k-l**, Comparing structures on the (+) and (-) strands for the 2 EV-D68 strains at cutoff of cov=0.05 and f=0.5.

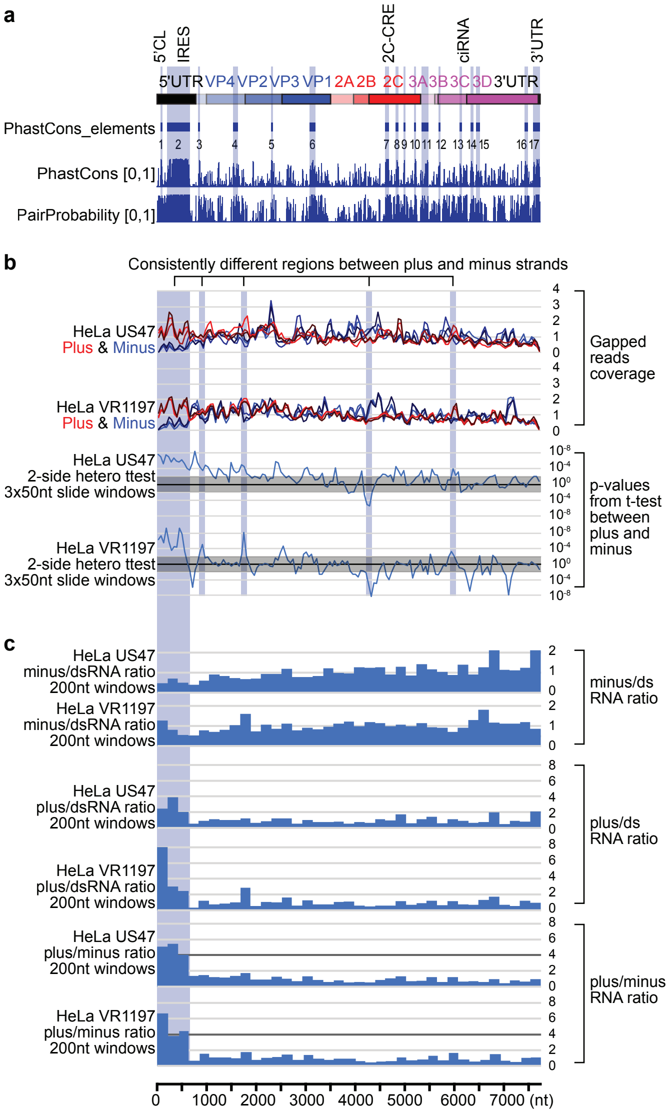

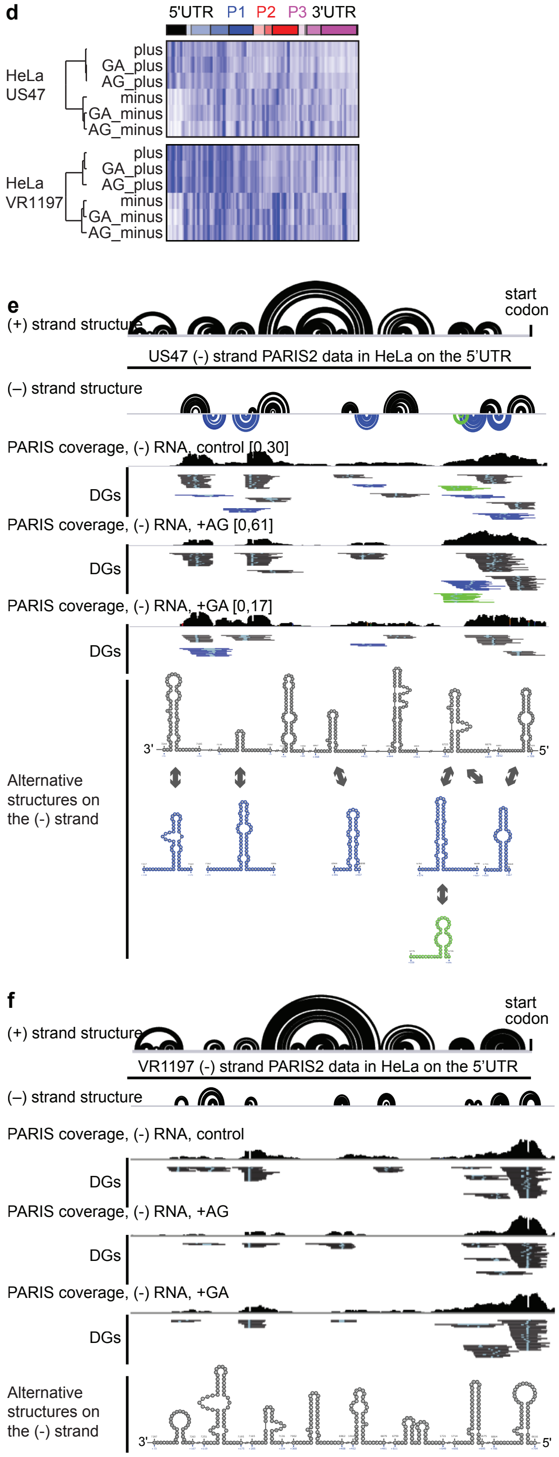

Supplementary Figure 30. Comparing structures on the plus (+) and minus (-) strands of EV-D68 genomes. a, EV-D68 genome annotation and conserved sequence elements. **b**, Comparing structures on the (+) and (-) strands in thw two strands. The 3 shades of red and 3 shades of blue were the normal, +AG and +GA inhibitor treated samples. The top two panels were the coverage of gapped alignments. All tracks were normalized so that the median was 1. The bottom 2 tracks were the p-values of two-sided unpaired t-tests between the three (+) and (-) strands. Consistently different regions between plus and minus strands were marked by the light blue shadows. **c**, Comparing the plus strand, minus strand and dsRNA (chimera of plus+minus strands) coverage across the genome for the two strains in 200nt windows. The last ~600nt on the plus strand (corresponding to the first ~600nt on the plus strand was significantly less structured. **d**, Unsupervised clustering of coverage for the plus and minus strands showing the difference between the two strands. **e-f**, Comparing the (+) and (-) strand structures on the 5'UTR of the (+) RNA. The long-range structures were lost on the (-) RNA in both strains. The (-) straind counterpart of the 5'CL structure was completely lost.

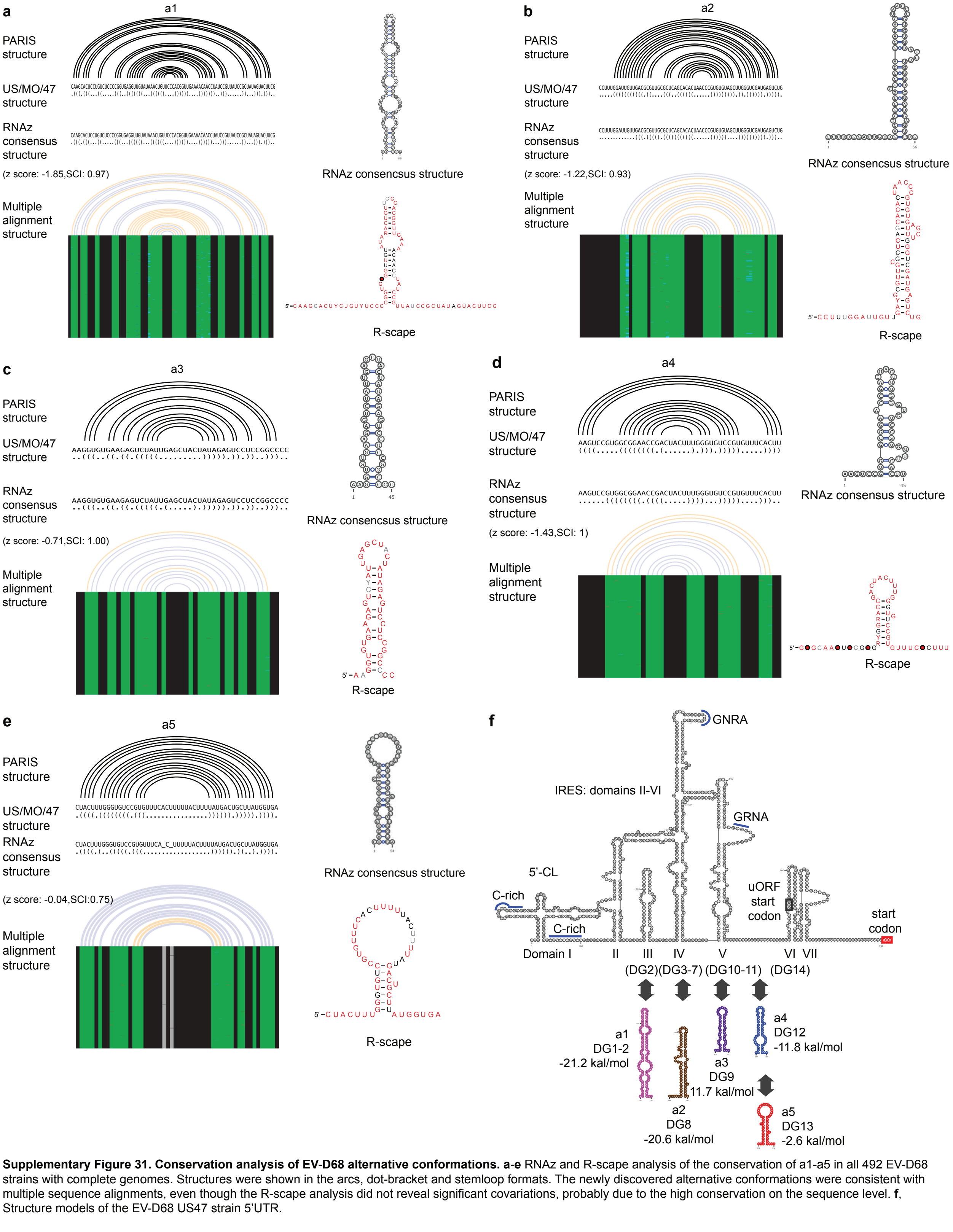

Supplementary Figure 31. Conservation analysis of EV-D68 alternative conformations. a-e RNAz and R-scape analysis of the conservation of a1-a5 in all 492 EV-D68 strains with complete genomes. Structures were shown in the arcs, dot-bracket and stemloop formats. The newly discovered alternative conformations were consistent with multiple sequence alignments, even though the R-scape analysis did not reveal significant covariations, probably due to the high conservation on the sequence level. **f**, Structure models of the EV-D68 US47 strain 5'UTR.

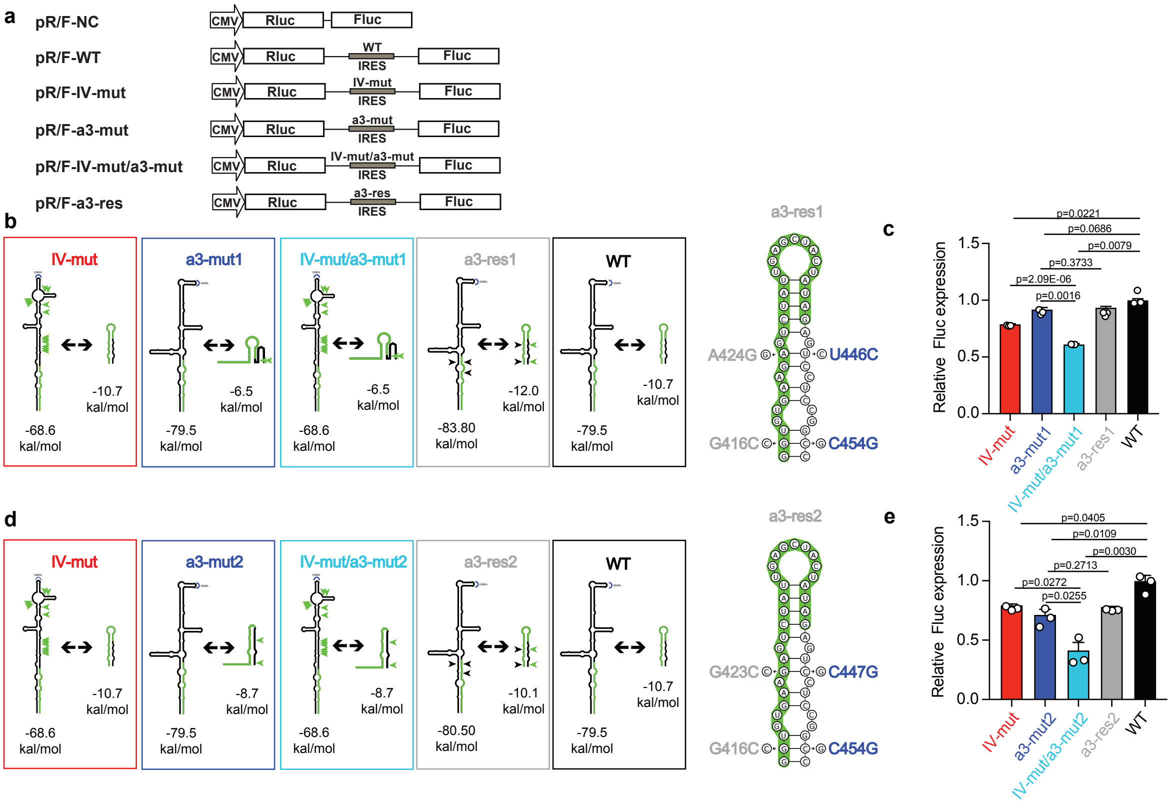

Supplementary Figure 32. Alternative structures play important roles in EV-D68 IRES activity. a, Schematic of bicistronic luciferase reporter plasmids. Various EV-D68 IRES fragments, including Wildtype, three mutants and compensatory rescue were inserted between the Renilla and firefly luciferase genes. The negative control in this assay had no insertion between these two genes. Renilla luciferase activity was measured to detect cap-dependent translation, while firefly luciferase activity was measured to detect IRES-mediated translation. IRES activity was calculated as the ratio of firefly luciferase activity to Renilla luciferase activity. **b,d**, Predicted secondary structure models of domain IV and alternative structure 3 (a3), including IV-mut only, a3-mut only, IV-mut + a3-mut, a3-res and WT (wildtype). The green arrows point to positions of mutations. The black arrows point to compensatory rescue mutations. The MFE values were shown below the respective models. **c,e**, Effects of domain IV and a3 mutations on IRES activity. HEK293T cells were transfected with pR/F-IRES plasmids, and 24 h later, firefly and Renilla luciferase activities were individually measured, and IRES activity was calculated as the ratio of firefly luciferase activity to Renilla luciferase activity. Data in **c** and **e** are mean ± SEM; n = 3 of biological replicates. two-tailed, unpaired t-test.

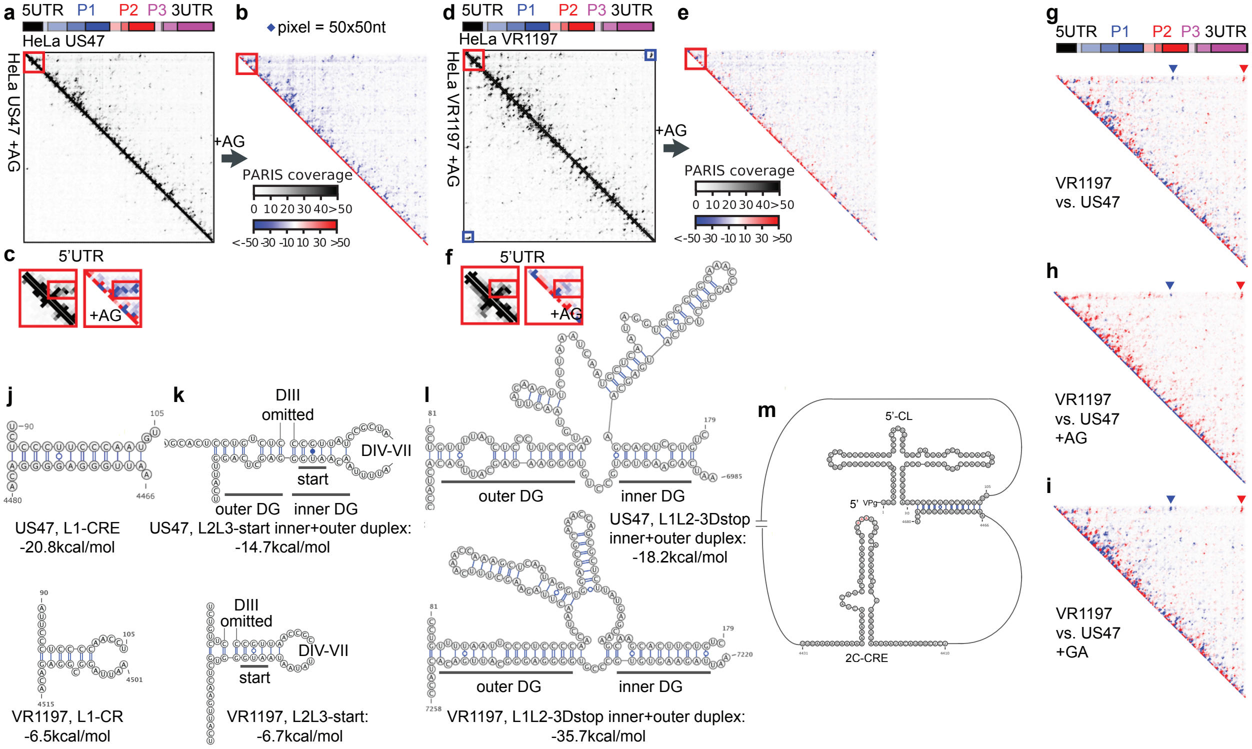

Supplementary Figure 33. Dynamic local and long-range structures associated with the EV-D68 5'UTR. a-f, The difference between normal and AG-treated EV-D68 (+) RNA structures, based on a comparison of heatmaps for US47 (a-c) and VR1197 (d-f) in HeLa cells. Gray scale was adjusted between the 2 conditions based on total read numbers. Given the read numbers, here we normalize all of them to 100000. Signal was averaged in 50nt x 50nt windows (each window in one pixel) **a,d**, RNA structures shown in a heatmap in normal and AG-treated HeLa cells. **b,e**, The normal structure density was subtracted from the AG treated condition. **c,f**, Zoom in to the 5'UTR in the two strains, showing the separate intensity (left) and difference (right). The L2L3-start was lost in US47 upon AG treatment. It is weaker in the VR1197 strain. **g-i**, Identification of differential dynamic long-range structures between US47 and VR1197. The heatmap is subtracting VR1197 from the US47 intensity. Blue arrow head: L1-CRE long-range structure. Red arrow head: L2L3-3Dstop long-range structure. **j-l**, Structures of L1-CRE, L2L3-start and L1L2-3Dstop in EV-D68 strains US47 (upper panels) and VR1197 (lower panels). The inner and outer DG (duplex) was calculated together in panels **k** and **l**. **k**, In the US47 strain, DIII forms a 3-way junction with two stems around the start codon. Domains III -VII are omitted in this diagram. The two stems around the start codon may stack together to form one continuous stem. This structure is lost upon AG treatment in both US47 and VR1197 strains. **m**, The big picture of the connection of L1 to the vicinity of CRE.

Sample preparation	Comparison	Average Ratio	Related figures
Amotosalen crosslinking<br>• 0.5 mg/ml amotosalen<br>• 2.0 mg/ml amotosalen<br>• 5.0 mg/ml amotosalen	0.5 AMT	3.42–6.95<br>• 3.42<br>• 5.13<br>• 7.95	Supplementary Fig. 2d
TNA method<br>• 0.5 mg/ml amotosalen<br>• 5.0 mg/ml amotosalen	TRIzol	2.13–6.48<br>• 2.13<br>• 6.48	Fig. 2k<br>Supplementary Fig. 3 and 6
DD2D gel purification	ND2D	1.54	Supplementary Fig. 8
Total improvement ratio<br>• 0.5 mg/ml amotosalen<br>• 5.0 mg/ml amotosalen	PATIS1	11.21–69.35<br>• 11.21<br>• 69.35	

Library preparation (after 2D gel)	Comparison	Average Ratio	Related figures
Adapter ligation<br>• SLRNA2<br>• SLRNA8	Standard	2.45–3.91<br>• 2.45<br>• 3.91	Supplementary Fig. 12
UVC protection (AO)<br>• based on *GAPDH* (70 bp)<br>• based on *ACTB* (184 bp)	no AO	6.51 - 9.90<br>• 6.51<br>• 9.90	Fig. 2m-n<br>Supplementary Fig. 10
PUVA bypass (SSIV)	SSIII	3.32	Fig. 2o
PUVA bypass (SSIV with Mn^{2+} buffer)<br>• based on *SONRD118* (96 bp)<br>• based on *SNORD13* (63 bp)<br>• based on *ACTB* (184 bp)	Mg^{2+} buffer	2.48 - 23<br>• 2.48<br>• 2.62<br>• 23.03	Fig. 2p<br>Supplementary Fig. 15
Total library yield	PARIS1	76	

Supplementary Table 1. Summary of PARIS2 improvements. The sample and library preparation improvements are listed separately. Sample preparation starts from crosslinking of cells to the 2D gel isolation of croslinked RNA. The library preparation starts from the 2D gel purified crosslinked RNA to the final DNA library ready for sequencing. See Supplementary Table 2 for detailed numbers of the improvements for every step. PUVA, psoralen plus UVA; AO, arcidine orange; SSIII, Superscript III; SSIV, Superscript IV. The TNA vs. TRIzol comparison is based on the improvement over all retrieved RNA from aqueous phase in TRIzol. Therefore the improvement of recovery for larger RNAs is even higher. The total library yield improvement of 76 fold was based on experimental results of library yield from the same amount of starting crosslinked RNA after 2D gel purification (not the same total RNA, see Supplementary Table 2). This number is close to the lower bound of the theoretical multiplied improvements for the library preparation steps (adapter ligation, UVC protection, PUVA bypass (SSIV) and PUVA bypass (SSIV with Mn2+ buffer)), which is in the range of [131, 2956]. This is because the several steps could be bottlenecks at the same time and improving one step may not be sufficient to lift the yield for the entire pipeline. The prevention of UVC-induced damage and bypass of PUVA-induced damage is positively correlated to RNA length. Longer amplicons contain more damage sites, and therefore the improvement is more significant.

**Step1. Amotosalen crosslinking**				
Crosslinked RNA fraction	0.5 AMT	0.5 Amoto	2.0 Amoto	5.0 Amoto
Rep1	0.92%	1.83%	3.43%	4.65%
Rep2	0.63%	2.71%		
Rep3	0.65%	1.72%		
Rep4	0.45%	2.90%		
Rep5	0.70%			
Average fraction	0.67%	2.29%	3.43%	4.65%
Ratio (to 0.5 AMT)	1.00	3.42	5.13	6.95

**Step2. TNA method**								
RNA yield (μg) form 2 million cells	TRIzol method				TNA method			
	Ctrl	0.5 AMT	0.5 Amoto	5.0 Amoto	Ctrl	0.5 AMT	0.5 Amoto	5.0 Amoto
Rep1	20.06	6.86	6.00	3.18	19.6	13.34	11.98	18.64
Rep2	18.46	6.26	5.96	2.96	18.56	14.36	14.82	21.3
Rep3	NA	NA	NA	NA	16.82	11.54	11.44	19.78
Average yield	19.26	6.56	5.98	3.07	18.33	13.08	12.75	19.91
Riato (to TRIzol)					0.95	1.99	2.13	6.48

**Step3. DD2D**		
Crosslinked RNA fraction	ND2D	DD2D
Rep1	0.47%	0.70%
Rep2	0.35%	0.63%
Rep3	0.46%	0.65%
Average fraction	0.43%	0.66%
Ratio (to ND2D)		1.54
Note: ND2D data is from Figs. 1e of Lu. *et al.* Cell 2016.		

**Step4. Adapter ligation**			
	tested oligos	Control	New condition
Adj. Vol. (Int)	SLRNA2	40479770	158230455
	SLRNA8	26086600	64037635
Ratio	SLRNA2		3.91
Ratio	SLRNA8		2.45
Adj. Vol. (Int), gel quantification data by Image Lab 6.0 Software.			

**Step5. UVC protection (AO)**				
Fold change of cDNA yield (normalized to control)	*GAPDH*		*ACTB*	
	No protection	With protection	No protection	With protection
Rep1	0.09	0.63	0.04	0.91
Rep2	0.08	0.66	0.04	0.99
Rep3	0.15	1.41	0.04	2.28
Rep4	0.12	1.09	0.35	3.48
Rep5	0.09	1.09	0.14	1.07
Rep6	0.28	1.08	0.54	0.87
Rep7	0.27	1.52	0.29	2.07
Rep8	0.30	1.37	0.13	2.14
Rep9	0.18	NA	0.14	2.00
Rep10	0.14	NA	0.13	2.37
Average cDNA yield	0.17	1.11	0.18	1.82
Ratio (to no protection)		6.51		9.90

**Step6. PUVA bypass (SSIV)**		
Fold change of cDNA yield (normalized to SSIII)	SSIII	SSIV
Rep1	1.00	2.29
Rep2	1.00	3.01
Average ratio (to SSIII)		2.65

**Step7. PUVA bypass (SSIV with Mn^{2+} buffer)**			
Fold change of cDNA yield (normalized to Mg^{2+} buffer)	*ACTB*	*SNORD118*	*SNORD13*
Rep1	19.33	2.57	2.77
Rep2	21.48	2.46	2.47
Rep3	19.65	2.39	
Rep4	31.62		
Average ratio (to Mg^{2+})	23.02	2.48	2.62

**Total library yield**		
Library yield (nmol)	PARIS1	PARIS2
Rep1	0.38	60.19
Rep2	0.71	51.86
Rep3	1.59	93.17
Rep4	0.49	42.86
Rep5	NA	58.85
Rep6	NA	53.27
Average yield	0.79	60.03
Ratio (to PARIS1)		75.75
Note: Library products yield from 50 ng of crosslinked RNA.		

Supplementary Table 2. PARIS2 improvements for each step. The first 3 steps (1-3) are improvements of sample preparation (from cells to crosslinked fragments), while the last 4 steps (4-7) are improvements of library preparation. The total improvement of sample preparation is ~60-70 fold. The "total library yield" improvement of ~76 fold was based on experimental results ofrom 50ng starting crosslinked RNA after the 2D gel purification.

**METHOD**	**PARIS2**	**PARIS**	**SPLASH**	**LIGR-seq**	**COMRADES**
Crosslinker solubility	amotosalen (>200mg/ml)	AMT (1mg/ml)	bio-psoralen (unknown)	AMT (1mg/ml)	Psoralen-TEG azide (unknown)
Crosslink efficiency vs. AMT	> 6-fold at 5mg/ml	used at 0.5mg/ml	used at 0.3mg/ml (0.2mM)	used at 0.02mg/ml	used at 0.4mg/ml
RNA purification (0.5mg/ml AMT)	TNA (100% recovery)	S1/PK (low, ~ 30%)	TRIzol (low)	TRIzol (low)	RNeasy (low)
RNA purification (5mg/ml Amotosalen)	TNA (100% recovery)		TRIzol (low)	TRIzol (low)	RNeasy (low)
Antisense enrichment	Yes	No	lost heavily crosslinked	lost heavily crosslinked	lost heavily crosslinked
RNA fragmentation	Optimized RNase III	S1 and RNase III	Mg^{2+} (90–110 nt)	S1	RNase III
Selection of crosslinked fragments	DD2D (100% pure)	ND2D(100% pure)	low (have monoadducts)	low (have monoadducts)	low (have monoadducts)
RNA damage	prevented/bypassed	severe	severe	severe	severe

Supplementary Table 3. Comparison of PARIS2 to other related methods that directly captures RNA base pairing information in cells. Methods that do not capture direct base pairing information, such as MARIO, RPL and RIC-seq, are not included. hiCLIP and CLASH are only applicable to protein-bound structures and not generally applicable to RNA duplex discovery. Only major differences are discussed here. The amotosalen that we used here has the highest known solubility among all psoralens, enabling crosslinking at much higher concentrations. At 5mg/ml, it has at least 6-fold higher crosslinking efficiency than AMT used at 0.5mg/ml. The solubilities of Bio-psoralen (EZ-Link™ Psoralen-PEG3-Biotin from Thermo Fischer) and Psoralen-TEG azide are not known, but they are only used at much lower concentrations, likely due to their lower solubility and much higher cost. Four different methods have been used in RNA purification, TRIzol (SPLASH, LIGR-seq), RNeasy (COMRADES), S1/PK+TRIzol (PARIS) and TNA (PARIS2). The S1/PK+TRIzol method increases yield over typical TRIZol but makes it impossible to enrich specific RNAs using antisense oligos. As shown in Fig. 2 and Supplementary Fig. 6, for cells crosslinked with 0.5mg/ml AMT, TNA improves yield by ~2-fold compared to TRIZol and RNeasy. For cells crosslinked with 5mg/ml amotosalen, TNA improves yield by 8-15 folds compared to TRIZol and RNeasy. Given that crosslinked longer RNAs are preferentially lost in TRIzol and RNeasy purifications, the TNA method improvement is more dramatic. Antisense oligo-based RNA enrichment is possible with TRIzol and RNeasy purified RNA, but the yield is much lower, given that most crosslinked RNAs are lost. The optimized RNase III fragmentation is also better than other methods. Either S1 or RNase III used alone results in broad size distribution, limiting the resolution of duplex models. Magnesium fragmentation leads to 5'-OH and 3'-phosphate that need to be repaired before proximity ligation. Both DD2D and ND2D gels yield pure crosslinked fragments, whereas other methods recover both crosslinked ones and fragments with psoralen monoadducts, which increase with higher crosslinker concentration (see Supplementary Fig. 10). The dominance of monoadducts leads to higher background in other methods. Our newly developed damage prevention and bypass together improves yield by 8-70 folds compared to the default conditions used in all previous methods.

	HEK293T mRNA	Mice brain mRNA
Total input reads	42,236,572	25,215,538
Primary alignments	22,641,746	14,559,941
continuous alignments	20,184,157	12,652,537
gap1 alignments	1,179,743	1,087,852
filtered gap1 alignments	627,884	740,304
gapm alignments	18,578	17,880
filtered gapm alignments	11,436	13,746
trans alignments	1,193,628	775,401
homotypic alignments	4,342	4,278
bad alignments	61,298	21,993
Filtered gap1+gapm+trans alignments	1,832,948	1,529,451

Supplementary Table 4. Library statistics for the mRNA enrichment. The primary alignments were filtered to remove low-confidence segments, rearranged and classified into 6 types using gaptypes.py (https://github.com/zhipenglu/CRS-SANT). Gap1: non-continuous alignments with one gap; gapm: non-continuous alignments with more than one gaps; trans: continuous alignments with the two arms on different strands or chromosomes; homotypic: non-continuous alignments with the two arms overlapping each other. Gap1 and gapm alignments containing splicing junctions and short 1-2 nt gaps were filtered out before further processing. Then filtered gap1 alignments, filtered gapm alignments and trans alignments were combined and used to analyze RNA structures and interactions.

Name	Sequence(5'-3')	Length
**Crosslinking oligos**		
ssDNA1-25mer	ACAGGGAAGGGTTATCCCACCTGAC	25 nt
ssDNA2-25mer	GTCAGGTGGGATAATCCTTACCTGT	25 nt
ssDNA3-25mer	ACAGGGAAGGGTTATGCCGCCTGAC	25 nt
ssDNA4-25mer	GTCAGGCGGCATAACCCTTCCCTGT	25 nt
ssDNA-8mer	CGGTACCG	8 nt
ssRNA-8mer	CGGUACCG	8 nt
**Primer extension oligos**		
RNA template	CUUGCUAGGCCCGGGUUCCUCCCGGGCCUAGCCCUGUC UGAGCGUCGC	48 nt
DNA primer	GCGACGCTCAGACAGG	16 nt
**Adapter ligation oligos**		
SLRNA1	UUGGUCAACGCGAGUUGACC	20 nt
SLRNA2	GGUCAACGCGAGUUGACCUU	20 nt
SLRNA3	AGGUCAACGCGAGUUGACCU	20 nt
SLRNA7	UUCCUUCCUCAACGCGAGUUGACC	24 nt
SLRNA8	CCUCAACGCGAGUUGACCUUCCUU	24 nt
**Library generation oligos**		
Adapter	/5rApp/AGATCGGAAGAGCGGTTCAG/3ddC/	21 nt
RT primer	/5phos/WWWNNNATCACGNNNNNTACCCTTCGCTTCA CACACAAG/iSp18/GGATCC/iSp18/TACTGAACCG C	56 nt
P3Tall	GCATTCCTGCTGAACCGCTCTTCCGATCT	29 nt
P6Tall	TTTCCCCTTGTGTGTGAAGCGAAGGGTA	28 nt
P3Solexa	CAAGCAGAAGACGGCATACGAGATCGGTCTCGGCATTC CTGCTGAACCGCTCTTCCGATCT	61 nt
P6Solexa	AATGATACGGCGACCACCGAGATCTACACTCTTTCCCC TTGTGTGTGAAGCGAAGGGTA	59 nt
**EV-D68 antisense oligos**		
EV-D68_US47_428	GAGGACTCTATAGTAGCTCA/3BioTEG/	20 nt
EV-D68_US47_1647	AAAGGTATGTTGGGACACCT/3BioTEG/	20 nt
EV-D68_US47_2553	AATTCTCCACTAGAGTCTCG/3BioTEG/	20 nt
EV-D68_US47_3366	CTGATTGCCAATCCACATAG/3BioTEG/	20 nt
EV-D68_US47_4369	CAAACCGGTTCAATGCGAGA/3BioTEG/	20 nt
EV-D68_US47_5518	GTCAAGTCTCTAAGTGCACA/3BioTEG/	20 nt
EV-D68_US47_7042	TTCATTGGCATCACTGGATG/3BioTEG/	20 nt

Name	Sequence(5'-3')	Length
**RT-qPCR primers**		
human-ACTB-F	AGAGCTACGAGCTGCCTGAC	20 nt
human-ACTB-R	AGCACTGTGTTGGCGTACAG	20 nt
human-GAPDH-F	CCATGAGAAGTATGACAACAGCC	23 nt
human-GAPDH-R	GGGTGCTAAGCAGTTGGTG	19 nt
human-SNORD118-F	TGGGATAATCCTTACCTGTTCCT	23 nt
human-SNORD118-R	TCCTGATTACGCAGAGACGTTA	22 nt
human-SNORD13-F	GTGATGATTGGGTGTTCATACG	22 nt
human-SNORD13-R	CACGTCGTAACAAGGTTCAAGG	22 nt
human-18SrRNA-F	CTTAGAGGGACAAGTGGCGTTC	22 nt
huamn-18SrRNA-R	ACGCTGAGCCAGTCAGTGTA	20 nt
EV-D68_US47_2C-F	GTGGAAGCAAAGAGGGTAGTAG	22 nt
EV-D68_US47_2C-R	GTTCCTGGAGAGCCATGTATTAT	23 nt
**Luciferase reporter assay primers**		
US-IRES-F1	CGGGGTACCCCCAATGTAACTTAGAAGTTC	30 nt
US-IRES-R1	ATAGTTTAGCGGCCGCTGTTAAAATTTTCAAATTAAAG	38 nt
IVm-fF1	CGTTGGCGGCCTACGCATCGTGAAAGCCATGAGACCGC AGCGGTGAACAAGGTGTGAAG	59 nt
IVm-fR1	AGGCCGCCAACGCAACCGGGACCACTGTCGCCAGTGAG GTATGGATAGACTCATCGACC	59 nt
a3m-1-fF1	ATAGAGCCCTCCGGGCCCTGAATGCGGCTAATC	33 nt
a3m-1-fR1	GCCCGGAGGGCTCTATAGTAGCTCAATAG	29 nt
a3m-r-1-fF1	GCTGTGAAGGGTCTATTGAGCTACTATAGAGCCCTCCG GGCCCTGAATGCGGCTAATC	58 nt
a3m-r-1-fR1	AATAGACTGTTCACAGCTTGTTCATGTCTAGCGTC	35 nt
a3m-2-fF1	ATAGAGTGCTCCGGGCCCTGAATGCGGCTAATC	33 nt
a3m-2-fR1	GCCCGGAGCACTCTATAGTAGCTCAATAG	29 nt
a3m-r-2-fF1	GCTGTGAACAGTCTATTGAGCTACTATAGAGTGCTCCG GGCCCTGAATGCGGCTAATC	58 nt
a3m-r-2-fR1	AATAGACTGTTCACAGCTTGTTCATGTCTAGCGTC	35 nt
IVm-r-fF1	GTACCAGCTTTGGATTGTTGACGCGTTG	28 nt
IVm-r-fR1	AATCCAAAGCTGGTACTAGGTTTCTCGAAG	30 nt
NC-F	CGGGGTACCGGATCCGGACTTAGACACGCCATTACTC	29 nt
NC-R	ATAGTTTAGCGGCCGCTGCTGCTATGAACCCTATCAAG	48 nt
**Human U8 kncok down ASO**		
/52MErG/*/i2MErG/*/i2MOEA/*/i2MErT/*/i2MErT/*A*T*C*C*C*A*C*C*T*G*/i2MOE rA/*/i2MOErC/*/i2MOErG/*/i2MOErA/*/32MOErT/		20 nt

Supplementary Table 5. Oligos used in this study.

Supplementary Notes

Optimized photochemistry enables efficient analysis of dynamic RNA structuromes and interactomes in genetic and infectious diseases

Minjie Zhang[1,4], Kongpan Li[1,4], Jianhui Bai[1,4], Willem A. Velema[2], Chengqing Yu[1], Ryan Van Damme[1], Wilson H. Lee[1], Maia L. Corpuz[1], Jian-fu Chen[3] and Zhipeng Lu[1,*]

In these supplementary notes, we provide a historical account of the technical challenges in the field of RNA duplex analysis using photochemical crosslinkers, physical and chemical mechanisms of these problems, and detailed descriptions of the optimizations. Much of the valuable information regarding RNA photochemistry was difficult to find in the literature, and most of the new results are not included in the main text due to limited space. In addition to the optimal conditions that we discovered, we also present negative data on alternative approaches that we have attempted, in the hope that these data will be useful for other researchers who are interested in further optimizations. Some of the studies, although not useful for improving PARIS, revealed fundamental principles of RNA physics and chemistry.

List of supplementary notes.

Supplementary Note 1.	Phase partition of crosslinked RNA and development of the TNA method
Supplementary Note 2.	Optimization of RNA fragmentation
Supplementary Note 3.	Developing the DD2D gel separation method
Supplementary Note 4.	Protection of RNA against UVC damage
Supplementary Note 5.	Bypass of PUVA induced oxidative damage on RNA

Supplemental Note 1. Phase partition of crosslinked RNA and development of the TNA method

<u>1. Background about the AGPC method.</u>

The classical acid guanidinium thiocyanate-phenol-chloroform (AGPC) method has been considered a gold standard in RNA extraction [1] (cited more than 70,000 times by April 2020). This method uses guanidinium thiocyanate (GuSCN), one of the strongest chaotropic agent, and acidic phenol, a denaturant, to disrupt biological materials and stabilize RNA. After adding chloroform, cellular components partition to the two liquid phases according to polarity/hydrophobicity: RNA in the upper aqueous phase, DNA in the interphase, and proteins and lipids in the interphase and lower organic phase (**Fig. 2e-f**). In contrary, the neutral phenol-chloroform isoamyl alcohol (PCI) method uses near neutral pH (~8) to partition DNA to the upper aqueous phase.

The original AGPC method used a mixture of solution D (4M GuSCN, 25mM sodium citrate, pH 7; 0.5% sarcosyl, 0.1M 2-mercaptoethanol (RNase inhibitor)), 0.2M sodium acetate pH 4, and water saturated phenol at 1:0.1:1 ratio [1]. Later, the protocol was modified so that all components are combined in a monophasic solution: 0.8M GuSCN (0.5-2M range), 0.4M ammonium SCN (0.1-0.6M range), 0.1M sodium acetate, pH 5 (4-6 range), 38% phenol (30-50 range), 5% glycerol (3-10% range) [2]. The 5% glycerol was used to blend the components of different polarity into one phase. This method has been commercialized in several kits, such as TRIzol, QIAzol and TRI reagent, and more recently, RNAzol, that were widely used in RNA research. The TRIzol LS (liquid sample) reagent has a proprietary composition, but it likely contains higher concentrations of these components, especially GuSCN, phenol and sodium acetate, so that lower volumes of TRIzol are used for liquid samples. The AGPC method allows quantitative recovery of pure RNA without any degradation of this labile molecule. Almost all cellular RNA molecules >20nt can be completely recovered, with one exception. The Kim lab reported that short structured miRNAs with low GC content are selectively lost during TRIzol extraction, leading to artifacts in miRNA quantification [3].

The theoretical basis of the phase partition of RNA and DNA in the aqueous-organic systems at different pH remains poorly understood. No quantitative analysis of the hydrophobicity/polarity has been published, to the best of our knowledge. Some researchers suggested that the lower pH (4-5) neutralizes the negative charge on DNA, which caused the DNA to be more hydrophobic (see brief overview of the method and its history by Paul Zumbo, "*Phenol-chloroform Extraction*"), but we believe this is not true. The phosphate backbone has a lowest pKa around 1-2, much lower than the pH range 4-5 used for RNA extraction, and thus should remain negatively charged (**Fig. 2e**).

<u>2. Psoralen crosslinked RNA partitions to the interphase in TRIzol extraction</u>

When we used TRIzol to extract RNA from AMT crosslinked cells, we noticed that the yield reduced as crosslinking strength increased (**Supplementary Fig. 3a**). Crosslinking cells with 0.1mg/ml AMT for 30min reduced yield to ~60%, and produced insoluble material in the TRIzol lysate from cells. The insoluble material promoted emulsion formation after addition of chloroform (see Fig. 2 from [4]), and then partitioned to the interphase after phase separation. Higher concentrations of psoralen resulted in higher crosslinking efficiency and even lower recovery of RNA from the aqueous phase (**Fig. 2b-c**). Initially we suspected that RNA was crosslinked to proteins and selectively trapped in the interphase. To test this hypothesis, we performed proteinase K (PK) treatment prior to TRIzol extraction. PK treatment consistently but modestly increased yield; there was still significant loss of RNA (**Supplementary Fig. 3a** and Supplemental data from [5]). This was consistent with previous reports that psoralen can crosslink nucleic acids to proteins, although at much lower efficiency than between nucleic acid strands [6, 7].

<u>3. S1/PK digestion as a temporary solution to recover crosslinked RNA.</u>

We used higher concentrations of AMT and amotosalen for PARIS experiments, which lead to even lower RNA recovery, down to ~20-30% (**Fig. 2b-c** in this paper and **Fig. S1** from [5]). To improve yield, we used S1 nuclease, which is active on DNA and RNA in cell lysates even under extremely highly denaturing conditions, such as 9M urea and 0.1% SDS [8]. Lysate digestion with S1 nuclease in 4M urea and 0.1% SDS led to higher yield of crosslinked RNA, especially in comparison with non-crosslinked samples (see **Fig. S1** from [5]). Together, the S1 nuclease and PK treatment led to sufficient RNA yield and was successfully used in our initial PARIS method, but there were still two problems. First, we were not sure if all crosslinked RNA has been recovered. Second, RNA fragmentation prior to extraction made it difficult to perform targeted RNA enrichment, for example using oligo(dT) for mRNAs, or RNA-specific antisense oligos.

<u>4. Psoralen crosslinking increases RNA hydrophobicity.</u>

The earlier observation that PK treatment plus TRIzol purification did not completely recover all crosslinked RNA suggest that crosslinked RNA itself became more hydrophobic. To confirm that AMT crosslinking alters RNA hydrophobicity, we purified normal total RNA from cells for crosslinking in vitro, and then directly precipitated RNA from the solution or used the standard TRIzol method to purify RNA (**Fig. 2g**). Non-crosslinked RNA was extracted efficiently in both methods (slightly lower yield from TRIzol, compared to direct ethanol precipitation, because of incomplete recovery of the aqueous phase), while crosslinked RNA was lost from standard TRIzol extraction, compared to direct ethanol precipitation. Noncrosslinked RNA appears as sharp peaks in the small RNA (50-300nt), 18S and 28S peaks (first 3 panels), while the crosslinked RNA shows as a broad smear spanning the entire profile (4th panel). The electrophoresis profile showed clear separation of noncrosslinked and crosslinked RNA between the aqueous and inter+organic phases (last two panels). While stronger crosslinking lead to gradual loss of RNA from the aqueous phase, more RNA accumulates in the interphase (**Fig. 2h**). These results suggest that crosslinked RNA is more hydrophobic, and partitions to the interphase, and is therefore lost during TRIzol extraction. Given that proteins were removed prior to RNA purification, the increased hydrophobicity was likely due to crosslinked RNA itself.

To further confirm that crosslinking increased RNA hydrophobicity, we tested addition of formamide in the standard TRIzol purification. Adding formamide greatly increased the partition of RNA to the aqueous phase (**Supplementary Fig. 3b**). Together, these results proved that crosslinking increases RNA hydrophobicity, making it similar to DNA, which normally partitions to the interphase during standard TRIzol extraction (see **Fig. 2f** for a summary).

<u>5. Most crosslinked RNA is in the interphase of TRIzol-chloroform mixture</u>

In order to confirm that most crosslinked RNA is in the interphase, we digested RNA from the aqueous and inter+organic phase using RNase III and ran a DD2D gel to separate crosslinked and noncrosslinked RNA (**Supplementary Fig. 3c-d**). From the same amount of RNase III digested RNA, we recovered similar amounts of crosslinked RNA from the aqueous phase and inter+organic phase (**Supplementary Fig. 3e**, both the columns “stuck in 1D” and “2D upper diagonal” are crosslinked RNA). Given that there is significantly more RNA in the interphase than the aqueous phase (**Fig. 2h**), these results suggest that in standard TRIzol purification, most crosslinked RNA are stuck in the interphase. For example, assuming that 20% RNA was recovered in the aqueous phase in standard TRIzol extraction, then more than 80% crosslinked RNA is lost. More importantly, larger RNAs are preferentially lost, leading to bias in the results.

<u>6. Smaller crosslinked RNA do not partition to the interphase.</u>

In summary, our studies showed that, first, psoralen crosslinks RNA to proteins, in addition to among nucleic acids, and second, crosslinking increases RNA hydrophobicity and causes it to re-partition to the interphase. To test whether we can use the difference in the hydrophobicity between crosslinked and noncrosslinked RNA to enrich for crosslinked RNA fragments during TRIzol extraction, we crosslinked RNA in vitro and then digested RNA using S1 nuclease and RNase III to make most RNA < 150nt. After the two digestions, we added TRIzol to the solution and then added chloroform, however, no clear interphase was observed, suggesting that the small crosslinked RNA fragments did not partition to the interphase. Furthermore, the precipitate from the inter+organic phase was not soluble in water and the Nanodrop profile shows major peaks at 230nm and 270nm, clearly different from RNA (260nm). Together these results showed that large crosslinked RNA, but not small crosslinked RNA fragments partition to the interphase, consistent with previous results (**Fig. 2g**). Therefore, we cannot selectively purify small crosslinked RNA fragments from the interphase using TRIzol.

<u>7. Developing the new TNA method.</u>

In the studies described above, we discovered that PK was needed to improve recovery of crosslinked RNA, and crosslinking made RNA more hydrophobic. Based on these observations, we decided to develop a new method to efficiently purify crosslinked RNA. First, we tested direct precipitation of total nucleic acids (TNA) from PK digested lysates from crosslinked cells. To obtain intact RNA, we searched for lysis conditions that would effectively inhibit all nucleases but at the same time allow efficient PK digestion. The TRIzol solution inhibits proteinase K, so it is not appropriate for cell lysis. Guanidine thiocyanate (GuSCN) is one of the strongest chaotropic agent, and at above 4M can denature most proteins, including nucleases [1, 9]. Therefore, we first lysed cells in 4M GuSCN (1 volume cell pellet + 2 volumes 6M GuSCN, pH ~5.3), and this usually leads to a clear solution, even for the crosslinked samples. Then we diluted the lysate with phosphate buffered saline (PBS) to 1M GuSCN and added EDTA to chelate divalent cations and performed PK treatment on cell lysates at 37C for 1 hour. The PBS dilution of GuSCN solution resulted in some insoluble material, which was then cleared by PK, suggesting most proteins were mostly digested. However, addition of 1 volume isopropanol (relative to the sum of cell pellet, PBS, GuSCN and EDTA.) lead to precipitates that could not be dissolved in water (**Supplementary Fig. 3f,** blue bars). Surprisingly, adding TRIzol before isopropanol precipitation lead to precipitates that

could be dissolved, suggesting that the TRIzol components, probably phenol, help keep the residual proteins in solution during isopropanol precipitation (**Supplementary Fig. 3f**).

To confirm that it was phenol that kept proteins in solution, we compared TRIzol and phenol during isopropanol precipitation (**Supplementary Fig. 3g**). Indeed, adding phenol and isopropanol was sufficient to produce RNA pellets that could be dissolved in water. Both ethanol and isopropanol can be used for precipitation. We preferred isopropanol in precipitation because less volume is needed. The disadvantage is that isopropanol is less volatile, and salts are less soluble in isopropanol, which may result in excess salt precipitation (Paul Zumbo, "*Phenol-chloroform Extraction*"). The salt precipitation problem can be resolved by extended 70% ethanol washes or reprecipitation.

8. Apparent lower yield of crosslinked RNA due to hypochromicity of RNA structures.

While testing direct ethanol precipitation of RNA from in vitro crosslinking, and the TNA method for nucleic acid extraction from crosslinked cells, we noticed that consistently less nucleic acids were recovered from the crosslinked samples (usually ~60-80%) and the A260/A280 ratio was lower (**Supplementary Fig. 3g**). We are certain that this is not caused by nucleic acid loss, because in vitro crosslinked pure RNA samples can be completely recovered. This was probably because crosslinking forced the formation of duplexes that absorb less UV light (1 OD260 Unit = 50ug/ml for dsDNA, or 40ug/ml for ssRNA, or 33ug/ml for ssDNA, or 20ug/ml for ssOligo) [10]. In fact, this higher percentage of double stranded regions in RNA, which is more hydrophobic, is consistent with our observation that crosslinked RNA is more hydrophobic, partitions to the interphase, like DNA, during standard TRIzol extraction. In addition, the selective crosslinking of uridines would also lead to different A260/A280 ratios as adenine and uracil are the major components in nucleic acids that absorbs UV light (see reference: Thermo Scientific T042-Technical Bulletin).

9. Removing DNA from total nucleic acids to recover all RNA.

Given that the TNA extraction protocol recovered total nucleic acids, next we performed DNase treatment to purify crosslinked RNA. Using Turbo DNase, we were able to digest away most DNA (**Fig. 2i-j**), and recover RNA that makes up ~40-60% of TNA. In practical applications, another round of DNase treatment can be performed if necessary, for example, after antisense enrichment of certain RNA populations. This second step of DNase treatment will be more efficient after antisense enrichment, given that the antisense enrichment will also reduce DNA contamination. Therefore, DNA contamination is not a concern in PARIS and similar experiments. In particular, real-time quantitative PCR with or without reverse transcription, a commonly used method, can be employed to assess the amount of DNA contamination. In our experience, one round of DNase treatment reduced DNA contamination to undetectable levels based on qPCR (Ct values always >40). Together, the above results demonstrated that we have developed a new method that is superior to the classical AGPC method in extracting crosslinked RNA.

10. Chlorambucil and carmustine crosslinking increases RNA hydrophobicity.

Is the crosslink-induced hydrophobicity a general property for RNA and different types of crosslinkers? To answer this question, we performed RNA crosslinking using two chemotherapy drugs chlorambucil (CHL) and carmustine (BCNU), which react with nucleic acids via different mechanisms (**Supplementary Figs. 4 and 5**).

Chlorambucil (CHL) is a nitrogen mustard that acts as a bifunctional alkylating agent and is used as a pharmaceutical agent, especially in chemotherapy (IARC 1987) (**Supplementary Fig. 4a**). During crosslinking, the aziridinium rings formed by intramolecular displacement of the chloride by amine nitrogen, alkylates DNA once it is attacked by the N-7 nucleophilic center on the guanine base. Then a second consecutive attack after the displacement of the second chlorine results in the formation of interstrand cross-links (ISC) (**Supplementary Fig. 4b**). The alkylation rates are limited by the rate of aziridinium ions' formation, and DNA ISC induced by CHL is formed at a specific site, 5'-GGC sequence (an 1,3 cross-link, G1-G3) by an DNA strand cleavage assay [11]. Using a synthetic DNA oligo duplex, we tested various concentrations and incubation time, and observed significant crosslinking after 3 hours (**Supplementary Fig. 4c-d**). Then we performed in vitro crosslinking of purified total RNA using CHL and observed strong smear that spans beyond the 28S rRNA peak, suggesting successful in vitro crosslinking (**Supplementary Fig. 4e**). We then precipitated the crosslinked RNA, digested RNA with RNase III and separated RNA using the DD2D gel system (**Supplementary Fig. 4f**). Crosslinked RNA was observed above the diagonal in the 2D gel. These results establish CHL as a strong RNA crosslinker.

To test whether the CHL crosslinking made RNA more hydrophobic, we crosslinked RNA, purified RNA using either direct ethanol precipitation, or the standard TRIzol method, where we extracted RNA from both the aqueous and inter+organic phases (**Supplementary Fig. 4g**). While non-crosslinked RNA was extracted from the aqueous phase efficiently (first three panels), crosslinked large RNA (e.g. 18S and 28S) partitioned to the interphase (bottom 3 panels), similar to psoralen crosslinked RNA (**Fig. 2g**). These results demonstrated that CHL crosslinking increased RNA hydrophobicity, similar to AMT and amotosalen crosslinking.

Carmustine (BCNU) is another category of commonly used chemotherapy drug, which can crosslink DNA in cells and is used for multiple cancers (**Supplementary Fig. 5a**). It belongs to chloroethylnitrosoureas (CENU), a new kind of alkylating agent which was developed later than nitrogen mustards. CENU exert cytotoxicity by inducing DNA interstrand crosslinks (ICLs) between guanine and the complimentary cytosine, namely dG–dC crosslink (**Supplementary Fig. 5b)**. The formation of a covalent connection between two DNA strands requires 2 successive reactions: (a) an alkylation or other modification of one strand; (b) a reaction of the modified strand with the complementary DNA strand (Kurt W. Kohn, 1977). We crosslinked purified total RNA, performed RNase III digestion and DD2D gel analysis. Crosslinked RNA fragments above the diagonal indicated that the crosslinking worked well (**Supplementary Fig. 5c-d)**. The crosslinking

process induces appreciable degradation (**Supplementary Fig. 5e**), however, this did not affect the experiments. Similar to the experiments on CHL and psoralens, we tested the partition of crosslinked RNA in the two phases during TRIzol extraction (**Supplementary Fig. 5f**). Despite the degradation, it was clear that larger crosslinked fragments are partitioned to the inter and organic phase (bottom panels).

Taken together, the analysis of chlorambucil and carmustine, two different categories of nucleic acid crosslinkers, confirmed that crosslinked RNA is more hydrophobic, the TNA method is generally applicable to crosslinked RNA, and the crosslinked fragments can be isolated using the DD2D gel system. These results showed that the crosslinking-induced hydrophobicity is not unique to psoralens, and is likely to be a general property of RNA. Although both chemotherapy drugs can crosslink RNA, they are not easily applicable to the analysis of RNA structures and interactions, because the crosslinks are not reversible.

11. Comparison among AGPC (TRIzol), TNA and silica-gel (RNeasy) methods

As an alternative to the phase partition approach in the AGPC method, silica gels (e.g. RNeasy kit from Qiagen and many other column-based nucleic aicd purification kits) have been used in the isolation of RNA and DNA from cell lysates. Nucleic acids absorb onto the silica in the presence of high concentrations of salt and chaotropic agents, and dissociate from silica at lower concentrations [12]. This method was recently used in another study to purify psoralen crosslinked RNA [13]. To compare the TNA method with the silica gel method, we first crosslinked pure total RNA with psoralen, then purified RNA with either direct ethanol precipitation or the RNeasy kit (**Supplementary Fig. 6a**). Compared to the TNA method, silica gel based method results in partial loss of crosslinked RNA (**Supplementary Fig. 6b**). Shorter RNAs, such as in the range of 50-300nt, are lost in the flow-through (**Supplementary Fig. 6c**).

To test recovery of RNA from psoralen crosslinked cells, we purified RNA using standard TRIzol, TNA and the RNeasy kit (**Supplementary Fig. 6d**). The TNA method retrieved all RNA, while TRIzol and RNeasy kit retrieved much lower amount RNA, and the retrieved RNA are significantly biased towards the lower end of size distribution (**Fig. 2k**, **Supplementary Fig. 6e**). The difference in performance was most dramatic at the highest crosslinker concentrations. For example, from cells crosslinked with 5mg/ml amotosalen, standard TRIzol and RNeasy methods lost more than 80% of the RNA. Together, these two sets of experiments showed that TNA is the best method for purifying crosslinked RNA. To the best of our knowledge, it is the only method capable of complete recovery of crosslinked RNA

12. Summary and discussion

While this study was in progress, several groups reported a new method for the isolation of crosslinked RNA-protein complexes based on their hydrophobicity [14-17]. The partition of crosslinked RNA-protein complexes to the interphase of an aqueous-organic mixture depends on the nonpolar amino acid residues in the protein part. This mechanism is different from the crosslinking/structure-induced hydrophobicity of RNA by itself.

We note that, in all previously published studies that employ psoralen crosslinking, it is very likely that most of the highly crosslinked large RNA were lost during purification [18]. Most previous in vitro and in vivo psoralen crosslinking protocols use way less AMT and much shorter time, which is why this abnormal behavior of crosslinked RNA has never been noticed. Earlier studies often focused on highly abundant RNAs, like rRNAs, snRNAs and snoRNAs, so the reduced sensitivity was not a problem. None of the recent studies that employ high throughput sequencing noticed or investigated this problem either [13, 19-21]. Some of the previous studies used PK to digest the samples after crosslinking, but only partially resolved the problem of low RNA recovery [22].

In summary, we made a surprising discovery that crosslinked RNA behaves differently from non-crosslinked RNA. Crosslinking increases RNA hydrophobicity, leading to its repartition to the interphase during standard TRIzol extraction. Based on this discovery, we have developed a new method to extract crosslinked RNA from cells. Our method represents a major breakthrough in solving this bottleneck problem and will greatly facilitate future studies using psoralen and other crosslinkers.

Supplementary Note 2. Optimization of RNA fragmentation

1. Introduction to the RNA fragmentation problem

Fragmentation of crosslinked RNA to small pieces is necessary for establishing secondary structures or RNA-RNA interactions at near base pair resolution. Various types of metal ion buffers and nucleases have been described, including RNase III (commercial name ShortCut from NEB, which produces dsRNA fragments above 18bp) [23], S1 nuclease, RNase A/T1, RNase I and magnesium (Mg^{2+}) [18]. These approaches differ in their cost, sensitivity to experimental conditions, product size distribution, product terminal chemistry (phosphate or hydroxyl on the 5' or 3' ends) etc. Initially, we tested the commonly used RNase T1 but found that it is difficult to control the digestion to obtain a narrow distribution of the fragment size (**Supplementary Fig. 7a**). RNase A/T1, RNase I, and divalent cations all produce 5' OH and 3'phosphate that require additional repair to make 5'phosphate and 3'hydroxyl for the next step of proximity ligation and adapter ligation.

2. Earlier optimizations of RNA fragmentation

We have chosen S1 nuclease and RNase III for several reasons [5]. First, S1 is active under highly denaturing conditions, such as 9M urea and 0.1% SDS. Second, S1 nuclease and RNase III both produces 5' phosphate and 3' hydroxyl (OH) that can be directly used for ligation and library preparation without further repair. Third, *E. coli* RNase III, when used with Mn^{2+}, cleaves dsRNA while protecting the products such that minimal RNA fragments are around 18-25bp [23], a suitable size window for our structure analysis (small enough for accurate modeling of base pairing, and big enough for mapping to the genome). To test whether S1 nuclease alone is sufficient for the fragmentation, we tested digestion for various time, but could not bring most of the RNA fragments to below 100nt even after prolonged incubation (**Supplementary Fig. 7b**).

3. Optimization of RNase III fragmentation of RNA.

While using the Native-Denatured 2D (ND2D) gel system to select crosslinked RNA fragments, we noticed that crosslinked and RNase III digested RNA tend to be bigger in apparent size than noncrosslinked RNA in the first dimension gel (see Fig. S1 from Lu et al., 2016). We reasoned that fragmentation conditions that amplify this difference could be used to isolate crosslinked RNA in a 1D gel alone, which would greatly reduce the time and effort of PARIS experiments and reduce loss of RNA during gel extraction. In fact, earlier studies have shown that *E. coli* RNase III does not only cleave dsRNA [24]. At lower ionic strength, it cleaves both ssRNA and dsRNA efficiently to small sizes (see Figure 3 in [24]. We found that the low ionic strength buffers did produce shorter fragments from the noncrosslinked RNA, mostly less than 40nt, while the digestion of crosslinked RNA gave rise to larger fragments (**Supplementary Fig. 7c,** significant tail above 40nt, indicated by the arrows). This result is consistent with previous studies of the wildtype E. coli RNase III [24]. However, when we run the 2D gels to select crosslinked RNA, the yield was consistently lower than before (~0.1%, compared to previous yield of 0.25-0.5%)[5], suggesting that the denatured 1D gel alone was insufficient for retrieving all crosslinked RNA fragments.

Given that RNase III can cleave both single and double stranded RNA, we sought to determine whether RNase III alone was sufficient for RNA fragmentation for the 2D gel separation. Varying the enzyme amount and incubation time, we found that the kinetics of the reaction determines the size distribution (**Supplementary Fig. 7d**). A short reaction time with high amounts of RNase III produced fragments that mostly lie in the range of 30-100nt (indicated by arrows), perfectly suited for 2D gel separation and library preparation. This kinetic effect is due to the tight binding of RNase III to the products, which inhibits further digestion of the short fragments [23]. High ratios of enzyme vs. RNA lead to efficient digestion leading to a more uniform size protected by the enzyme, while low ratios lead to both shorter fragments and longer fragments.

4. Summary and discussion

Here we presented a simplified strategy for RNA fragmentation that resulted in a narrow size distribution perfectly suited for RNA structure analysis and many other studies that require fragmented RNA. This method is better than other approaches for several reasons. First the protocol is simple, requiring only one enzyme. Second, the size distribution is narrow. Third, the products have 5' phosphate and 3' OH, suited for direct subsequent ligation reactions in library preparation and other types of enzymatic reactions.

Supplementary Note 3. Developing the DD2D gel separation method

1. Overview of the problem of enriching crosslinked RNA fragments

Several strategies have been used for enriching crosslinked RNA in recent high throughput analysis of RNA duplexes, including ND2D gel [5], biotinylated psoralen pull down [13, 21], and RNase R trimming of single stranded RNA [20] (**Supplementary Fig. 8a-c**). It has been shown that monoadducts are the major products in psoralen crosslinking, while only 20-40% of adducts are crosslinks in DNA [25]. Higher concentrations of psoralens are likely to cause higher ratios of monoadducts because less efficient sites are also forced to react with psoralens. We also found that RNA crosslinking is much less efficient, producing much more monoadducts than crosslinks (see details in **Supplementary Note 4** and **Supplementary Fig. 11**). Purification of biotinylated psoralen crosslinked nucleic acids recovers more monoadducts than crosslinks, dramatically reducing the sensitivity of the method (only a small percentage of RNA fragments can be used to produce proximally ligated RNA). This is in contrast to single chemical tagging reactions with target biomolecules, where the biotin handle is good enough for purifying the reacted molecules [26]. RNase R digestion of single stranded RNA is also blocked by the bulky monoadducts. As a result, the ND2D gel is the only method that ensures isolation of pure crosslinked RNA fragments. We calculated the percentage of gapped and chimeric reads in published methods, such as hiCLIP, SPLASH, LIGR-seq and COMRADES [13, 20, 21, 27], and found that PARIS [5], which used the ND2D gel method, consistently outperforms other methods, consistent with the idea that the 2D gel isolation of pure crosslinked RNA is essential for obtaining high percentages of gapped/chimeric reads (**Supplementary Fig. 8d)**.

2. The ND2D method and its problems

While performing the RNase III digestion and denatured gel purification of crosslinked RNA fragments, we noticed that the yield (~0.1%) is much lower than what we have achieved previously (0.23-0.55%) [5] (see Figure S1 in [5]). This low yield is likely due to the strong RNase III digestion that reduced size of certain crosslinked RNA to below 30-40nt. To solve this problem, we tested other nuclease fragmentation methods, including S1 alone, lighter RNase III alone, RNase A and RNase A/T1. While testing these conditions, we noticed that the 12% native and 20% denatured 2D gel cannot separate crosslinked fragments from non-crosslinked below 40-50nt. This observation prompted us to reexamine the theoretical basis of the 2D gel method.

Vigne and Jordan described the first native-denatured 2D (ND2D) gel system to analyze RNA structures [28]. In this pioneering study the authors showed that the second denatured dimension gel will separate an RNA duplex from the first native dimension and thus allow the identification of the two fragments held together by hydrogen bonding (not covalent bound). Zwieb and Brimacombe adapted ND2D gel for the analysis of crosslinked RNA fragments [29] (see diagram in **Supplementary Fig. 8a**). Here the crosslinked fragments run as a tight duplex in the first dimension and then opened up to an "octopus" shape in the second denaturing dimension and therefore ran much slower, separating from the non-crosslinked fragments. Thompson and Hearst applied the ND2D gel to analyze AMT-crosslinked RNA fragments [30]. However, the claim that "the crosslinked hairpin loops actually run slightly faster in the second dimension because their radius decreases somewhat" is likely to be an incomplete statement, because hairpins are likely to run slower upon denaturation. It has been shown that short circular RNAs (e.g. 20nt), which are constrained and base paired, run faster than their linear forms which would be more single-stranded in TBE-urea gel. We also noticed that in native gels, the singled stranded RNA runs much slower than an RNA duplex of the same molecular weight, suggesting that upon denaturation in the second dimension, stem-loops would run slower to match that of an RNA of the same molecular weight that was already single-stranded in the first dimension. This slow-down would mask some crosslinked dsRNA fragments in the second dimension.

3. The DD2D method for separating crosslinking RNA from noncrosslinked.

To solve this problem, we explored the possibility of running denatured-denatured 2D (DD2D) gel, where the gel percentage is lower in the first dimension than the second (**Supplementary Fig. 8b**). The rationale for this design is as follows: structured RNA molecules encounter higher friction in the higher percentage gels, therefore, they run much slower relative to their linear counterparts. In previous experiments, I noticed that the 113nt tricRNAs runs near its real size in a 6% gel, but close to 200nt in a 10% gel [31]. Earlier studies also showed that a 5% denatured – 10% denatured 2D system can be used to separate lariat RNAs from linear RNAs [32]. We tested several combinations of gel percentages in the first and second dimensions and found that the 8%+16% combination produced clear separation of crosslinked RNA from the noncrosslinked (**Supplementary Fig. 8e-g**). The crosslinked RNA forms a smear above the upper diagonal, or gets stuck in the 1D-2D interface. Compared to the ND2D gel, the recovery of crosslinked RNA increased by 50%, therefore increasing the sensitivity of the PARIS method (**Supplementary Tables 1 and 2**).

We found that RNA crosslinked with BCNU and chlorambucil can be easily separated from noncrosslinked fragments (**Supplementary Figs. 4 and 5**). As long as the density of the second dimension is different from the first dimension, the separation should work well. Therefore, combinations different from the 8%+16% gel system shown in this study can be used in other applications where either longer or shorter crosslinked fragments can be studied.

A caveat with this design is that shorter crosslinked RNA duplexes may not run slower in the higher percentage gel. This is based on the observation that while longer circular RNAs actually run slower than their counterpart, shorter circular RNAs run faster than their linear counterparts. Indeed, in our 8% denatured – 16% denatured 2D gels, we cannot see any crosslinked dsRNA below 50nt above the diagonal. Because of this, we need to make sure that the nuclease digestion keeps most RNA fragments above this range. Given that this new system does not separate RNA based on their base pairing status, it is generally applicable to all types of nucleic acid crosslinking studies.

Here is a simplified quantitative model for the DD2D separation of linear and structured nucleic acids.
$f(p) = \exp\{-\mathrm{k}_1 p\}$ for $p \in [p_1, p_2]$, speed dependence on gel concentration p. k_1 is a constant within certain range of p.
$g(l) = \mathrm{k}_2/\ln(l)$ for $l \in [l_1, l_2]$, speed dependence on nucleic acid length l. k_2 is constant within certain range of l.
$v_{\mathrm{L}}(p,l) = f(p) \cdot g(l)$, speed for linear nucleic acids
$v_{\mathrm{S}}(p,l,s) = f(p) \cdot g(l) \cdot h(p,l,s)$, speed for structured nucleic acids
$f(p)$ is a monotonically decreasing function of gel concentration, or increasing function of gel pore size (Stellwagen and Stellwagen 2009, Effect of the matrix on DNA electrophoretic mobility)
$g(l)$ is a monotonically decreasing function, inversely proportional to the logarithm of nucleic acid length within a certain range, therefore, $v_{\mathrm{L1}}/v_{\mathrm{L2}}$ still has some dependence on nucleic acid length (find original reference) (https://www.thermofisher.com/us/en/home/life-science/cloning/cloning-learning-center/invitrogen-school-of-molecular-biology/na-electrophoresis-education/na-separation-overview.html).
v_{L} is the migration speed of a linear nucleic acid.
v_{S} is for the structured, e.g. circular, branched, lariat, crosslinked duplexes, etc.
$v_{\mathrm{L,2D}}/v_{\mathrm{L,1D}} = f(p_{\mathrm{2D}})/f(p_{\mathrm{1D}}) = \exp\{-\mathrm{k}_1(p_{\mathrm{2D}} - p_{\mathrm{1D}})\}$, relative speed of linear nucleic acid for the same length

$(v_{\mathrm{S,2D}}/v_{\mathrm{S,1D}})/(v_{\mathrm{L,2D}}/v_{\mathrm{L,1D}}) = h(p_{\mathrm{2D}},l,s)/h(p_{\mathrm{1D}},l,s) \begin{cases} > 1, \text{if } l < l_0 \\ = 1, \text{if } l = l_0 \\ < 1, \text{if } l > l_0 \end{cases}$, relative speed of structured vs. linear nucleic acids.
l_0 is close to 30 in the case of circular RNAs. For example, we know $h(p,l,s) > 1$ for $l \leq 24$, and < 1 for $l > 37$, see discussion of the circular ssDNA C44, C60, C66 and C70 [33]. The dependence of migration speed on RNA structure s is more difficult to quantify. $h(p_{\mathrm{2D}},l,s)/h(p_{\mathrm{1D}},l,s)$ is a monotonically decreasing function of nucleic acid length and gel concentration, which is why on the higher percentage gel, the impact of gel concentration on structured nucleic acids is larger. The size of the crosslinked RNA fragments is difficult to estimate based on the denatured gel, because they run much slower than a linear RNA of exactly the same size. Earlier studies showed that in a 14% denatured polyacrylamide gel, a 72nt circular ssDNA runs close to 500nt linear ssRNA in size [33].

4. Summary and discussion

Here we showed that a denatured 2D gel with different density between the two dimensions effectively separates crosslinked from noncrosslinked nucleic acids. The DD2D method does not depend on specific structures of the crosslinkers or the RNA fragments, therefore it is generally applicable to all types of nucleic acid crosslinking experiments.

Supplementary Note 4. Protection of RNA against UVC damage

1. Introduction to the problem of photochemical damage of RNA

When preparing PARIS sequencing libraries, we noticed that the vast majority of the DNA in the final step of gel selection have very short inserts, many in the range of a few base pairs, even though the original crosslinked RNA fragments that we selected were at least 30-40nts [5]. These results suggest that additional factors besides proximity ligation lead to the small insert size, and we suspect that this is due to RNA damage during to AMT-mediated long-wavelength (UVA, 365nm) photo-crosslinking and subsequent short wavelength (UVC, 254nm) reversal. Psoralen + UVA (PUVA) primarily lead to oxidative damage, while UVC primarily induces the formation of cyclobutane pyrimidine dimers (CPDs), and several other forms of photoproducts, such as (6-4) pyrimidine dimers, the Dewar valence isomers, hydrates, oxidized bases (mostly 8oxoG) and single strand breaks [34-39]. Absorption of UVC photons produces RNA singlet and triplet excited states. The primary precursor of CPDs and other damages is the singlet state, while the triplet state only plays a limited role (less than 10%) [40, 41]. These damages could block reverse transcription and lead to both the lower overall cDNA yield and lower percentages of gapped reads (**Fig. 2l**).

Damages to nucleic acids in the form of pyrimidine dimers occur much faster than strand breaks. These pyrimidine dimers and hydrates lead to incomplete cDNA products similar to the consequences of failed proximity ligation (even if the proximity ligation worked). Recent studies showed that a short period of UV 254nm irradiation, even within a few seconds, can already strongly block reverse transcription [34, 42]. Given the broad application of UV in molecular biology, a general strategy for reducing photodamage is particularly important.

2. Earlier attempts to reduce UVC damage of RNA.

To reduce the impact of RNA damage on the percentage of gapped reads, we first evaluated the size selection. The shorter cDNA products due to incomplete reverse transcription can be selectively removed. This approach can increase the proportion of gapped reads from successful proximity ligation and processive reverse transcription. However, the size selection protocol can be difficult to establish given that the RNA and cDNA fragments are a broad smear. In addition, the total cDNA yield will be much lower. Other recent studies have attempted to minimize UV damage while maximizing the reversal efficiency by limiting the 254nm UV irradiation time, but the benefit is limited [21]. Given the fast photodamage (which may occur within a few seconds) and that at least 5-10min is needed for reversal of the crosslinks, reducing the reversal time is far from enough for optimal reversal and damage reduction.

3. PhrB (E. coli photolyase) failed to repair UV damaged RNA

In many organisms (except humans), a special enzyme called photolyase can repair CPDs in DNA (**Supplementary Fig. 9a**). In addition, photolyase possess residual activity towards RNA [43]. We tested two commercially available photolyases from E. coli on UVC damaged cDNA or RNA and then performed quantitative PCR to measure the repair efficiency. Modest repair was observed on 254nm UV damaged DNA, but not on RNA (**Supplementary Fig. 9b**, only one PhrB data was showed here). Prolonged incubation with PhrB even reduced the amount of intact RNA. Therefore, we conclude that the residual activity of PhrB is currently insufficient to repair pyrimidine dimers. Nevertheless, engineered PhrB with enhanced activity towards RNA may be useful for repairing RNA.

4. Stronger stacking did not speed up photoreversal of psoralen crosslinking or reduce UVC damage.

Earlier studies showed that T-Pso-T diadducts were much harder to reverse than crosslinked DNA oligo duplexes, suggesting a role of the structural context in efficient reversal [44-47]. Therefore, we tested whether stronger stacking could speed up UVC reversal and reduce the time needed and therefore reduce the damage. Addition of 1M NaCl to the RNA solution, which should help RNA form stable secondary structures, did not speed up the reversal or reduce the damage (**Supplementary Fig. 9c**). It is likely that the stacking of RNA base pairs was not significantly enhanced by the 1M NaCl. In other experiments, we found that the reversal is quite efficient in the DNA and RNA oligo duplexes, suggesting that speeding up the reversal is not a good strategy (**Supplementary Fig. 10i-l**).

5. Denaturing agents failed to prevent UVC damage of RNA.

The excited states of nucleobases that are the energetic precursors to CPDs are extremely short-lived and the formation of CPDs depend on the proper pre-alignment of the neighboring bases in stacked conformations before absorption of the photons. It has been shown that some solvents with hydrophobic groups to compete for base stacking interactions reduce crosslinking efficiency [48-50]. Therefore, we tested whether denaturing conditions can be used to disrupt stacking and reduce UVC induced CPD damage to RNA. Addition of DMSO or formamide to very high percentage did not prevent the damages (**Supplementary Fig. 9d**). This is probably because the properly stacked conformations still exist for a sufficiently long time to allow the dimers to form, despite the ability of these solvents to reduce stacking.

6. Variable efficiency of reverse transcriptases on UVC damaged RNA.

Several types of reverse transcriptases (RTs), including the TGIRT and MarathonRT, have been shown to be highly processive on structured RNA, even ones with chemical adducts [51-53]. UVC causes more dramatic changes in the RNA structure than adducts from structure probing, therefore, we need to identify enzymes that may be more processive on UV damaged RNA. First, we introduced UVC damage by irradiating pure total RNA with 254nm UV for 30min. Then we performed reverse transcription using six RTs, including AffinityScript, MarathonRT, TGIRT-III enzyme (TGIRT), Superscript (SS) II, III and IV (**Supplementary Fig. 9e**). Analysis of 4 RNAs showed that SSIV consistently outperformed other RTs on UVC damaged RNA. However, the 7-fold improvement is far from enough to bypass all damages since 30min UVC irradiation reduces qRT-PCR efficiency ~100-1000 fold for RNA amplicons in the range of 70-180nt (from *ACTB* and *GAPDH* mRNAs, **Fig. 4c, Supplementary Fig. 10c-d, g**).

7. Quenchers of nucleobase singlet excited states prevent UVC damages on RNA.

The quantum chemical mechanism of pyrimidine dimer formation in DNA has been studied extensively due to its critical role in UV-induced skin cancers. CPD formation in DNA primarily occurs through the singlet excited states and therefore quenching the singlets can be used to inhibit CPD formation [54-58] (see **Fig. 2m** for a diagram). Several types of singlet quenchers have been shown to inhibit CPDs, including DNA binding dyes proflavine, acridine orange (AO), ethidium bromide (EtBr), methyl green, dimeric Zinc(II)-Cyclen complexes, and the organic solvent acetone [59] (**Supplementary Fig. 10a**). These dyes can bind to double strand nucleic acids by intercalating between adjacent base pairs or by exterior ionic bonding. UVC irradiation in the presence of singlet quenchers can even lead to reversal of DNA pyrimidine dimers [55, 60].

However, later studies did not find proflavine or AO effective in protecting RNA against UVC irradiation, suggesting that differences in DNA and RNA structure may affect the protection efficiency [61]. The lack of protection could be due to the lower concentrations used in these studies (5uM for proflavine and 50uM for AO) and the lower affinity of these dyes toward RNA compared to DNA. Nevertheless, Merriam and Gordon found that the singlet quencher methanol partially protects RNA from UVC damage [56-58]. Methanol used at 90% concentration did not lead to complete protection, suggesting that its protective effect is limited, and more efficient quenchers are needed. Furthermore, many of these dyes absorb light in the UVC range (e.g. **Supplementary Fig. 10b**), raising concerns of the usefulness of such quenchers in the reversal of psoralen crosslinks.

Kleopfer and Morrison showed that acetone has no effect on the dimerization of dimethyl thymidines or DMT, in contrast to the strong inhibition of CPD formation in E. coli DNA reported by Sutherland and Sutherland [49, 57]. This can be potentially explained by the different energetic precursor involved in CPD formation for base monomers vs. polymeric DNA [62]. Greenstock and colleagues showed that the triplet quencher oxygen inhibits dimerization of monomers, such as uracil and thymine, but not dimers like UpU and TpT. In other words, the nucleobase monomers form CPDs primarily through the triplet states that can be quenched by oxygen, while polymers form CPDs primarily through the singlet states that can also be quenched by certain singlet quenchers, such as the DNA dyes and acetone. These studies suggest that singlet quenchers, but not triplet quenchers, are the most likely candidates for protecting RNA against UVC damages.

Based on these previous studies, we set out to systematically test singlet quenchers for protecting RNA from UVC-induced damages (**Supplementary Fig. 10c-h**). All the singlet quenchers protected RNA to various extent. EB and AO showed the highest efficiency at 2.5mM concentrations, protecting *ACTB* mRNA (180nt PCR amplicon) by ~1000 fold, and *GAPDH* mRNA (70nt RNA amplicon) by ~50 fold (**Supplementary Fig. 10c-d**). Even RNAs that were treated with PUVA before UVC irradiation was also protected (**Supplementary Fig. 10e-f**). For the PUVA treated samples (*ACTB* AMT and *GAPDH* AMT), the absolute quantitative PCR Ct changes (log2(fold) change) after UVC damage with or without prevention were much lower than the non-PUVA treated samples (**Supplementary Fig. 10,** compare panels **c to d** and **d to f**). This is because PUVA lead to additional damages that blunt the UVC damages. The UVC reversal of psoralen crosslinks in the presence of EB and AO lead to net increase in amplifiable RNA compared to crosslinked but not-UVC treated samples (**Supplementary Fig. 10e-f,** EB and AO bars). The level of protection strongly depends on the quencher concentration (**Supplementary Fig. 10g-h)**. The much higher concentrations needed to protect RNA (2.5mM), vs. for DNA (e.g. 50uM AO) can be explained by the lower affinity of these dyes towards RNA [54-58].

We found that AO had a higher prevention efficiency against UVC damages at a lower concentration (0.25 mM) compared to EB (2.5 mM) (**Supplementary Fig. 10g-h**). In order to better remove these quenchers after crosslink reversal, we chose lower concentration of AO (0.25 mM) to prevent UVC damages (**Fig. 2n**). Using the 30min UVC irradiation to reverse crosslink, the protection of AO for *ACTB* mRNA was 62.6-fold (from 0.005 to 0.313) without PUVA, and 6.46 with PUVA. The protection of *GAPDH* mRNA was 29.18-fold (from 0.016 to 0.467) without PUVA (**Fig. 2n**). For PUVA treated samples, there is also PUVA-induced oxidative damages (see **Supplementary Note 5** for details) and simultaneous reversal of crosslinking, so it is much more difficult to calculate the contribution of the singlet quencher effect.

Higher concentrations of acridine orange are needed to protect RNA given its lower binding affinity compared to DNA. To test whether higher affinity nucleic acid intercalators are more effective, we used a dimer of EB, EthD1 (**Supplementary Fig. 10a**), which has a binding affinity of $2 \times 10^{8} M^{-1}$, compared to $1.5 \times 10^{5} M^{-1}$ for EB [63, 64]. However, we found that the high binding affinity intercalators were difficult to remove from the RNA samples after reversal, and the protection was not higher than EB. The SYBR dyes, SYBR Green I and SYBR Gold did not protect RNA well compared to EB and AO (**Supplementary Fig. 11**). The SYBR dyes and acetone partially protected DNA against UVC damages, while AO almost completely protected DNA. Even for DNA that was first crosslinked with psoralen, there was little reduction in PCR efficiency after the 254nm reversal, suggesting little damage after both PUVA and UVC treatment (**Supplementary Fig. 11**). Together these results showed both similarities and differences in the susceptibility and prevention of UVC damages on DNA and RNA.

8. Calculating damages and prevention using the Poisson distribution.

For these calculations, we focus on the normal RNA (without psoralen crosslinking), because the crosslinking induces other types of damages. We assume that the dimers follow a Poisson distribution [61], $P(k) = \lambda^k e^{-\lambda}/k!$, where the interval is defined as the RNA sequence to be reverse transcribed, e.g. 70nt for the *GAPDH* amplicon; λ is the average number of dimers in the RNA sequence interval; k is the number of dimers in the RNA sequence interval, and takes the values of 0, 1, 2 …. k has an upper limit determined by the sequence length and base composition.

For example, there are 18 potential pyrimidine dimer sites in the *GAPDH* mRNA amplicon, 11 of which are in the actual reverse transcribed region, with 7 at the primer binding site. The effect of the dimers on reverse transcription depends on their locations, for example, different places within the primer binding sites or in the actual reverse transcribed region to be amplified. Each dimer in the actual reverse transcribed region only partially blocks reverse transcription, therefore, the actual effect on RT need to be determined by a separate factor. We do not know the exact bypass percentage but it is very low based on previous studies [65]. For simplicity, we define a complete damage site as one that completely blocks the RT. The following calculations are based on **Supplementary Fig. 10c-d** for *ACTB* and *GAPDH* mRNAs.

mRNA	UVC	AO	delta_Ct	% intact RNA	Complete damage sites	Protection % vs. w/o AO
ACTB	10min	–	-5.64	2.0	3.91	
ACTB	10min	+	-0.92	52.9	0.64	84
ACTB	30min	–	-11.07	0.05	7.67	
ACTB	30min	+	-1.27	41.5	0.88	89
GAPDH	10min	–	-3.39	9.5	2.35	
GAPDH	10min	+	-0.81	57.0	0.56	76
GAPDH	30min	–	-6.62	1.0	4.59	
GAPDH	30min	+	-0.99	50.3	0.69	85

In the presence of AO, ~50% RNA remained intact after 10-30min irradiation. The addition of AO protected both mRNAs by 76%-89%. UVC-induced CPD dimerization is a reversible reaction [54], therefore, the protection will not be 100%, unless all pyrimidines are completely sequestered by quenchers or other types of blockers (see quantitative analysis as follows). In addition, other less frequent types UVC-induced damages, such as strand breaks and oxidative damages, are not reversible, further limiting the extent of protection.

To understand the UVC damage and prevention, using AO as an example, here we treat the process as an equilibrium.

$$M_{quenched} \overset{AO}{\leftrightarrows} M \underset{k_2}{\overset{k_1}{\rightleftarrows}} D, \qquad r_1 = k_1[M], \qquad r_2 = k_2[D]$$

Here M stands for monomer, D stands for dimer. Both k_1 and k_2 are unimolecular reaction constants that depend on UV wavelength. When AO is present, it affects the effective [M] and UV dose at the same time. Increasing AO reduces [M] and effective UV dose, which will require longer time to reach equilibrium. In reality there will always be forward reaction as long as [M] is positive. At equilibrium $r_1 = k_1[M] = r_2 = k_2[D]$, therefore, $[M]/[D] = k_2/k_1$, the ratio of [M]/[D] will not change at a fixed wavelength. This analysis shows that it is impossible to completely prevent UVC induced pyrimidine dimers.

9. Singlet state quenchers do not block photoreversal of psoralen crosslinks

To study whether AO will block the reversal of crosslinking, we designed 8-mer DNA and RNA oligos to perform crosslinking and reverse crosslinking test (**Supplementary Fig. 10i-l**). DNA oligos were crosslinked much more efficiently than RNA oligos (compare panel i vs. k). Most RNA oligos only formed monoadducts. Then, these crosslinked products were reversed by 254nm UV, with or without AO. The reversal was completed within 10min, and the presence of AO did not block reversal (**Supplementary Fig. 10k, l**). These results suggest that the absorption of UV light by singlet quenchers does not affect reversal significantly (**Supplementary Fig. 10b**).

10. Summary and discussion

In this systematic optimization, we have discovered that extensive dimers caused the low efficiency of reverse transcription, reducing the yield of cDNA and percentage of gapped reads. We found that SSIV reverse transcriptase was more efficient on UVC damaged RNA. Several intercalating dyes and solvents previously found to protect DNA from UVC damage by quenching the singlet states of excited DNA can also protect RNA from UVC damage, although much higher concentrations are needed. Importantly, these dyes do not block the reversal of psoralen crosslinks.

In summary we developed the first method for efficiently protecting RNA against UVC damage. This important discovery will prove useful for many problems in RNA biology given the broad application of UV irradiation and the growing interests in RNA structure analysis using photochemical crosslinking. For example, this method can be useful for other RNA experiments that involve UVC irradiation, such as analysis of RNA structures by chemical probing [34, 42], and RNA-protein crosslinking studies [66].

Supplementary Note 5. Bypass of PUVA induced oxidative damage on RNA

The combination of photosensitizers and UVA irradiation causes extensive oxidative damage to nucleic acids, including base oxidations and subsequent strand breaks [38, 39, 67, 68]. RNA is likely subject to more oxidative damages than DNA due to its location in cells and the structural differences [69, 70]. Such damages will block reverse transcription and reduce cDNA yield in library preparation [71]. In early studies, we noticed that crosslinking induced extensive DNA fragmentation, but few strand breaks on RNA. This is probably due to the DNA repair on psoralen adducts that induce double strand breaks. The photosensitized oxidations also damage other cellular components, including proteins and lipids. The oxidative damages, in addition nucleic acid crosslinking, have been suggested as a major factor in the therapeutic, as well as side effects of PUVA therapies. In PARIS, the protection of RNA against UVC damage was insufficient to keep RNA intact. Therefore, we set out to determine the extent of PUVA induced oxidative damages and develop approaches to prevent, repair or bypass these damages.

1. PUVA induces RNA damages

First, we used cDNA yield of RT-qPCR to test PUVA damages on RNA molecules. We found that PUVA, but not UVA alone, blocked reverse transcription (**Supplementary Fig. 13a-b**). Lower concentration of AMT (0.05 mg/ml) induced less damage, but also reduced crosslinking efficiency (**Supplementary Figs. 13c-d**). In order to reduce the PUVA damages, we also test the effects of separating the incubation and crosslinking steps to reduce effective psoralen concentrations during crosslinking. HEK293T cells were incubation with 0.5 mg/ml AMT solution for 15 mins, to make sure AMT penetrate into RNA duplex (**Supplementary Figs. 13e-f**). Then AMT solution was changed to PBS solution to perform crosslinking by 365 nm UV for 30 mins. This design slightly reduced RNA damage, but also greatly decreased crosslinking efficiency due to dissociation of AMT from nucleic acids after washing out AMT.

2. Antioxidants reduce PUVA damage but also block psoralen crosslinking.

Next, we explored the possibility of using antioxidants and reactive oxygen species (ROS) quenchers to reduce PUVA-induced damages (**Supplementary Fig. 14a-b**). Psoralen (AMT) can serve as a photosensitizer for UVA, which involves a direct energy transfer reaction between triplet states of excited AMT and ground state oxygen, producing highly reactive ROS that can oxidize RNA base and generate PUVA damages on RNA (**Supplementary Fig. 14b**). In addition, guanine has the lowest one-electron oxidation potential of the nucleobases. The excited singlet state of intercalated AMT can directly react with DNA/RNA molecules, especially guanine, resulting radical guanine and further predominantly from oxidation of guanine. All these PUVA damages will also block the reverse transcription efficiency.

Earlier studies have shown that certain antioxidants and ROS quenchers can reduce PUVA-induced oxidative damages using both in vitro and in vivo models. These chemicals act at different stages of the oxidation process. For example, O_2•- scavenger: Tiron and MnTBAP [72]; •OH scavenger: Mannitol [73, 74], DMSO and Glycerol; $^1O_2$ scavenger: NaN_3 [67, 75], general radical scavenger: Vitamin C (VC) [76, 77] (**Supplementary Fig. 14a**). The effects of ROS scavengers on PUVA damaged RNA were tested by RT-qPCR and found to be highly variable (**Supplementary Fig. 14c-g**). For example, mannitol and NaN_3 had no effect on PUVA damages, Tiron and MnTBAP partially blocked damages, while VC almost completely blocked damages at 100mM concentration. However, the reduction of damages was accompanied by loss of crosslinking (**Supplementary Fig. 14h-j**), suggesting that these compounds quenched a common mechanism in crosslinking and photooxidative damage.

Recent studies showed that psoralen crosslinking involves an electron transfer from DNA to AMT, similar to the electron-transfer induced guanine oxidation [78, 79]. In particular, intercalated psoralen (AMT) is excited to singlet state, and directly induces electron transfer from DNA, charge recombination and crosslinking for pyrimidines, or oxidation for guanines. Together, studies presented here and in earlier publications suggest that it is impossible to block oxidative damage without blocking crosslinking. Furthermore, our studies further suggest that, during PUVA therapy, the protective effects of antioxidants and ROS scavengers are at least partially based on their abilities to block crosslinking, in addition to blocking the oxidation of cellular components.

3. DNA polymerases cannot bypass PUVA-induced RNA damages.

We noticed that PUVA-treated DNA can be amplified in PCR without obvious reduction of Ct value in PCR, suggesting that DNA polymerases can bypass the oxidative damages on DNA samples. Several earlier studies showed that certain DNA polymerases also possess reverse transcriptase activity [80-84]. A few of them, e.g. Bst, Klenow LF, Klenow exo-, were reported to have comparable levels of RT activity to AMV RT for templates shorter than 125nt [85]. Therefore, we tested the possibility of using DNA polymerases in bypassing PUVA-induced oxidative damages on RNA. However, despite extensive tests of conditions, we were unable to obtain comparable levels of cDNA yield using the DNA polymerases (**Supplementary Fig. 15a**).

4. Optimized RT conditions improve bypass of PUVA-induced oxidative damages

Earlier studies showed reverse transcriptases can bypass oxidative damages pn RNA but the efficiency is very low [86]. Multiple types of oxidized guanines can be bypassed to various degrees. During reverse transcription, the enzyme rackets between active and inactive states [87], and the RNA damages trap the RT enzymes in the inactive state for much longer time. Van Nostrand et al. found that reverse transcriptase read-through RNA with peptide adducts was highly dependent on the identity of the reverse transcriptase enzyme as well as on buffer conditions [88]. Therefore, we systematically screened for reverse transcription conditions to increase bypass efficiency.

Higher ratios of enzyme:substrate increase processivity, especially for MMLV derived reverse transcriptases, such as superscript II, III and IV [89]. Because RNA-DNA hybrids can sequester reverse transcriptases and reduce available enzymes for cDNA synthesis, longer incubation time also helps improve the cDNA products yield [89]. We first tested higher amounts of RT enzyme SSIV in reverse transcription. HEK293T cells were crosslinked by 365 nm UV for 30 mins with 0.5 mg/ml AMT, then reverse crosslinking was performed with the protection of acridine orange. Reverse transcription with higher amounts of SSIV did not result in higher cDNA yield (**Supplementary Fig. 15b**).

Next we tested various RT enzymes on PUVA damaged RNA, using primer extension assays and reverse transcription PCR. For the primer extension assay, we crosslinked a 48-mer RNA template oligo to mimic the PUVA damages (**Supplementary Fig. 15d**). After photo-reversal by 254 nm UV with AO protection, reverse crosslinked RNA oligo was used to primer extension assay. For the PCR assay, we used primers targeting several mRNAs and noncoding RNAs. SSIV outperformed other RT enzymes, including MarathonRT and TGIRT, both of which were previously reported to be highly processive on structured and modified RNA molecules [51-53] (**Supplementary Fig. 15c, f,h**).

Several studies suggested that Mn^{2+} induce more bypass of RT blocks in many different conditions, including chemical adducts in SHAPE experiments, and peptide adducts in CLIP experiments [88, 90]. We tested RT bypass of PUVA damages under different concentrations of Mn^{2+}. Primer extension assays and RT-qPCR data suggested that SSIV with 1.5 mM Mn^{2+} buffer is the most effective on PUVA damaged RNA (**Supplementary Fig. 15g,i**).

The racketing behavior of RT enzymes between active and inactive states suggests that longer incubation may increase the bypass of damaged RNA. Therefore, we tested the effect of different RT incubation time using primer extension and RT-qPCR assays (**Fig. 2o-p, Supplementary Fig. 15j-m**). The longer incubation time dramatically improved cDNA yield for several different RNA targets. Together these optimizations identified best combination of conditions that improve RT efficiency on PUVA damaged RNA.

<u>5. Summary and discussion.</u>

In summary, our extensive studies of PUVA-induced oxidative damages clarified important mechanisms in the process. We found that certain antioxidants and ROS scavengers can reduce oxidative damage, but also block crosslinking at the same time. Our in-depth analysis of the reverse transcriptase conditions suggests new variations that greatly improve reverse transcriptase processivity and cDNA yield. We found that the reverse transcriptase activity on damaged RNA can be enhanced by several treatments, including the SSIV variant of enzyme, cofactor Mn^{2+}, and much longer incubation. The prolonged incubation is especially effective in promoting the bypass.

Supplementary Methods

In these supplementary methods, we provide step by step protocol of PARIS2.

1. Psoralen (e.g. AMT) Crosslinking:

1) Wash 10 cm dish cells with 1X PBS twice;
2) Add 200 µL 2X PBS, 200 µL 1 mg/mL AMT to each dish;
3) Put cells at 37ºC for 15 mins;
4) Place ice trays in the cross-linker and put cell dish on ice. Irradiate cells with 365 nm UV for 30 mins. Swirl the plates every 10 mins and make sure that they are horizontal.
5) Remove cross-linking solution after cross-linking and wash cells twice with 1x PBS.

2. TNA (total nucleic acid) extraction from psoralen crosslinked cells:

6) For each 10 cm dish cells, add 100 µL of 6 M GuSCN, lyse cells with vigorous manual shaking for 1 min. The cells should be lysed into a nearly homogenous solution, which may not be entirely clear. Be careful, as the 6 M GuSCN is highly corrosive.
7) Then to each tube add 12 µL of 500 mM EDTA, 60 µL of 10x PBS, and bring the volume to 600 µL with water. This dilution of the sample will lead to some insoluble material. Then pass the sample through a 25G or 26G needle about 20 times to further break the insoluble material.
8) Add proteinase K to 1 mg/ml (30 µL from the 20 mg/mL stock), mix well and incubate at 37 ºC for 1 hour on a shaker (eg: Thermomixer C), at 600-900 RPM. Manually shake the tubes a few times during the incubation to facilitate mixing.
9) After PK digestion, add 60 µL of 3 M sodium acetate (pH 5.3), 600 µL of water-saturated phenol (pH 6.7), mix well divide into two tubes and then to each tube add 600 µL of pure isopropanol.
10) Spin down the precipitate at 15000 rpm for 20 min at 4 ºC and remove supernantant (dispose of phenol waster properly).
11) Wash the precipitate with 70% ethanol twice to remove residual phenol and other contaminants. In each wash, mix well and shake vigorously before spinning down.
12) Combine the TNA pellets from two tubes and resuspend in 300 µL od nuclease-free water for each 10 cm plate of cells.
13) Determine the concentration and quality of the TNA sample using Nanodrop and Tape station.

3. DNase I Treatment:

14) Transfer 100 µg of TNA samples to a new tube. Add 20 µL of 10X TURBO™ DNase Buffer, 25 µL of TURBO™ DNase (2 Units/µL). Bring each sample to a final reaction volume of 200 µL using H_2O.
15) Incubate samples at 37ºC for 20 min.
16) Add 20 µL of 3 M sodium acetate (pH 5.3), 220 µL of water-saturated phenol (pH 6.7), 450 µL of pure isopropanol, mix well. Spin 20 mins at 12,000 x g at 4 ºC. Wash pellet twice with 70% Ethanol.
17) Resuspend RNA samples in 50 µL of RNase-free water.

4. Shortcut Digestion:

18) Transfer 10 µg of DNase treated RNA sample to a new tube.
19) Add ShortCut mix (tabulated below) to each sample and incubate at 37ºC for 5 mins;

Component	Amount (µL)	Final Concentration
10x ShortCut buffer,	4 µL	1x
50 mM $MnCl_2$	4 µL	5 mM
ShortCut RNase III	10 µL	0.5 U/µL
RNase-free water	Up to 40 µL	

20) Add 4 µL of 3 M sodium acetate (pH 5.3), 3 µL of GlycoBlue, 60 µL of phenol, 360 µL of pure ethanol, mix well. Spin 20 mins at 12,000 x g at 4 ºC. Wash pellet twice with 70% Ethanol.
21) Resuspend RNA in 10 µL of RNase-free water. Determine concentration of the samples by spectrophotometer and analyze size distribution using Tape station.

5. 2D gel purification:

5.1 First dimension gel:

22) Prepare the 8% 1.5 mm thick denatured first dimension gel using the UreaGel system. For 10 mL gel solution, use 3.2 mL of UreaGel concentrate, 5.8 mL of UreaGel diluent, 1 mL of UreaGel buffer, 4 µL of TEMED, and 80 µL of 10% APS. Add TEMED and APS right before pouring the gel.
23) Use 15-well combs so that each lane is narrower and the second dimension has a higher resolution.
24) To each 10 µL sample add 10 µL GBLII loading dye. Load 200 ng dsRNA ladder as molecular weight marker. Run the first dimension gel at 30 W for 7~8 mins in 0.5X TBE.
25) After electrophoresis finishes, stain the gel with 2 µL of SYBR Gold in 20 mL 0.5X TBE, incubate for 5 min. Image the gel using 300 nm transillumination (not the 254 nm epi-illumination, which reverses the psoralen cross-linking).

Excise each lane between 50 nt to topside from the first dimension gel. The second dimension gel can usually accommodate three gel splices.

5.2 Second dimension gel:

26) Prepare the 16% 1.5 mm thick urea denatured second dimension gel using the UreaGel system. For 20 mL gel solution, use 12.8 mL UreaGel concentrate, 5.2 mL UreaGel diluent, 2 mL UreaGel buffer, 8 µL TEMED, and 160 µL 10% APS.
27) To make the second dimension gel, put the square plate horizontally and arrange gel slices in a "head-to-toe" manner with 2–5 mm gap between them. Leave 1 cm space at the top of the notched plate so that the second dimension gel would completely encapsulate the first dimension gel slices.
28) Apply 20–50 µL 0.5X TBE buffer on each gel slice to avoid air bubbles when placing the notched plate on top of the gel slices.
29) Remove the excess TBE buffer after the cassette is assembled, and leave 2 mm space at the bottom of the notched plate to facilitate pouring the second dimension gel.
30) Pour and gel solution from the bottom of the plates, while slightly tilting the plates to one side to avoid air bubbles building up between the plates. If there are air bubbles, use the thin loading tips to draw them out.
31) Use ~60ºC prewarmed 0.5X TBE buffer to fill the electrophoresis chamber to facilitate denaturation of the cross-linked RNA. Run the second dimension at 30 W for 50 min to maintain high temperature and promote denaturation. The voltage starts around 300 V and gradually increases to 500 V, while the current starts around 100 mA and gradually decreases to 60 mA.
32) After electrophoresis, stain the gel with SYBR Gold the same as the first dimension gel.

5.3 Purification:

33) Excise the gel containing the cross-linked RNA from the 2D gel and transfer it to a new 10 cm cell culture dish. Crush the gel by grinding with the cap of a 15 mL tube.
34) Add 300 µL crushing buffer to gel debris. Transfer the gel slurry to a 15 mL tube by shoveling with a cell scraper.
35) Add additional 1.2 mL crushing buffer and rotate at room temperature overnight.
36) Transfer ~0.5 mL gel slurry to Spin-X 0.45 µm column. Spin at room temperature, 3400X g for 1 min. Continue until all gel slurry is filtered.

Component	Quantity (50 mL)	Final Concentration
Tris–HCl (1 M, pH 7.5)	1 mL	20 mM
Sodium acetate (2.5M, prepared without pH adjustment)	5 mL	0.25 M
EDTA (0.5 M, pH 8.0)	0.1 mL	1 mM
SDS (10%, w/v)	1.25 mL	0.25%
RNase-free water	42.65 mL	Up to 50 mL

If the SDS precipitates, warm to 37°C until the precipitate disappears. Store indefinitely at room temperature.

37) Aliquot 500 µL of the filtered RNA sample to an Amicon 10 k 0.5 mL column. Spin at 12,000 X g for 5 min. Repeat until all of the filtered RNA sample flowed through the column.
38) Wash the column with 300 µL water and spin the column at 12,000X g for 5 min.
39) Invert and place the column in a new collection tube, and spin at 6000 X g for 5 min. Recover ~85 µL RNA from each column (~170 µL total from two columns).
40) Precipitate the RNA using the standard ethanol precipitation method, with glycogen as a carrier. Alternatively, the RNA can be purified using the Zymo RNA clean and concentrator-5 columns.
41) Reconstitute RNA in 11 µL water and dilute 1 µL RNA sample for Bioanalyzer analysis. The RNA sample should have a broad size distribution between 40 and 150 nt in the Bioanalyzer trace. The yield is typically 0.1–0.5% from 10 µg input RNA.

6. Proximity Ligation:

42) Add 10 µL of proximity ligation to 10 µL of RNA, mix well and incubate at 65ºC for 20 mins.

Component	Amount (µL)	Final Concentration
10x 5' DNA Adenylation Reaction Buffer	2 µL	1x
Mth RNA Ligase	2 µL	100 pmol
SUPERase In	1 µL	1 U/µL
RNase-free water	5 µL	

43) Inactivate the enzyme by incubation at 85°C for 5 minutes.
44) Add Proteinase K to 1 mg/mL, incubate at 37°C for 30 minutes.
45) Add 2 µL of 3 M sodium acetate (pH 5.3), 2 µL of GlycoBlue, 25 µL of phenol, 60 µL of isopropanol, mix well. Spin 20 mins at 12,000 x g at 4 ºC. Wash pellet twice with 70% ethanol. Resuspend RNA in 8 µL of RNase-free water.

7. Reverse crosslinking:

46) To reverse the AMT cross-linking, put the samples on a clean surface with ice beneath it. Add 2 µL of 25 mM acridine orange and mix well.
47) Irradiate with 254 nm UV for 30 min.
48) Transfer reverse crosslinked sample to a new tube. Add 190 µL of RNase-free water, 20 µL of 3 M sodium acetate (pH 5.3), 3 µL of GlycoBlue, 600 µL of pure ethanol, mix well. Spin 20 mins at 12,000 x g at 4 ºC. Wash pellet twice with 70% ethanol. Resuspend RNA in 6 µL of RNase-free water.

8. Adapter Ligation

49) Heat reverse crosslinked RNA at 80ºC for 90s, then snap cooling on ice.
50) Add 14 µL of adapter ligation mixture to 6 µL RNA and perform the adapter ligation reaction for 3 h at room temperature.

Adapter ligation mixture	Amount (µL)	Final Concentration
10x T4 RNA ligase buffer	2.0 µL	1x
0.1 M DTT	1.0 µL	5 mM
50 v/v % PEG8000	5.0 µL	12.5 % v/v
DMSO	2.0 µL	5%
10 µM ddc RNA adapter	3.0 µL	1.5 µM
High Concentration T4 RNA ligase 1	1.0 µL	1.5 U/µL

51) After adapter ligation add the following reagents to remove free adapters: 3 µL of 10X RecJf buffer (NEBuffer™ 2, B7002S), 2 µL of RecJf, 1 µL of 5' deadenylase, 1 µL of SuperaseIn, and 3 of µL water. Incubate at 37 oC for 1 h.
52) Add 20 µL of water to each sample (total volume of 50 µL) and purify RNA with Zymo RNA clean and Concentrator-5 or ethanol precipitation. Reconstitute RNA in 11 µL of RNase-free water (elute in 6 µL of water, use same 6 µL twice).

9. Reverse Transcription

53) To the purified RNA add 2 µL of custom RT primer (with barcode) and 1 µL of 10mM dNTPs.
54) Heat the samples to 65ºC for 5 min in a PCR block, chill the samples one ice rapidly.
55) Add 7.5 µL of reverse transcriptase mix to the RNA and heat the samples at 25 ºC for 15 min, 42 ºC for 10 hours, 80 ºC for 10 min; hold at 10 ºC.

SuperScript IV RT Master	Amount (µL)	Final Concentration
5x SSIV Mn2+ Buffer	4.0 µL	1x
100 mM DTT	2.0 µL	10 mM
SUPERaseIn	1.0 µL	1 U/µL
SuperScript IV	1.0 µL	5 U/µL

5x SSIV Mn2+ Buffer	Final Concentration
Tris-HCl (PH 8.3)	250 mM
CH_3COOK	375 mM
$MnCl_2$	7.5 mM

56) Add 1 µL RNase H and RNase A/T1 mix and incubate at 37 ºC for 30 min at 1000 rpm in a thermomixer.
57) Purify the cDNA using SPRI DNA beads. Add 2x volume of SPRI DNA beads, equal volume of isopropanol, mix well; Incubate for 5 min at RT. Let the beads settle on the magnet for 5 min. Remove the supernatant and wash the beads once with 80% ethanol (200 µL) at RT. Dry 2min. Elute twice with 8.5 µL water (recover ~16 µL).
58) Or:
59) Using DNA Zymo concentrator-5 columns (add 7x Binding Buffer, then equal volume (8x original) of 100% EtOH to bind, wash normal, elute in 2 x 8.5 µL of water).

10. cDNA Circularization, Library PCR, and Sequencing

60) Add 4 µL circularization reaction mix to the cDNA sample and incubate at 60 ºC for 100 min, followed by 80 ºC for 10 min.

CircµLarization Mix (4 µL)	Amount (µL)	Final Concentration
10x CircLigaseII Buffer	2.0 µL	1x
CircLigase II Enzyme	1.0 µL	5 U/µL
50 mM $MnCl_2$	1.0 µL	2.5 mM

61) Add 21.4 µL of PCR Tall mix and run PCR program until exponential amplification confirmed. Transfer cDNA to optical PCR tubes (each tube should be separate so that individual tubes can be taken out of the qPCR machine when the fluorescence signal reaches a defined point).

2x Phusion HF mix (100 µL)	Amount (µL)
5x HF buffer	40.0 µL
10 mM dNTP	4.0 µL
Phusion	2.0 µL
Water	54.0 µL

PCR Tall Mix (21 µL)	Amount (µL)
P3/P6 Tall (20 µM)	1.0 µL
Phusion HF 2x	20.0 µL
25x SYBR Green I	0.4 µL

62) Set up the following qPCR program. Choose SYBR, initial 98 ºC, 2 mins, 10 cycles of: 98 ºC, 15 s; 65 ºC, 30 s; 72 ºC, 45s, detect fluorescence at extension step (a set of nine cycles). Take sample out once amplification reaches exponential phase.
63) Transfer PCR product to 1.5 mL tube. Purify the DNA using SPRI DNA beads. Add 2x volume of SPRI DNA beads, mix well. Let the beads settle on the magnet for 5 min. Remove the supernatant and wash the beads once with 80% ethanol (200 µL) at RT. Dry 2min. Elute twice with 10.5 µL water (recover ~20 µL).
64) Repeat SPRI DNA beads purification one more time.
65) Pool elute and add 21 µL 2X PCR Solexa mix.

PCR Solexa Mix (21 µL)	Amount (µL)
P3/P6 Solexa (20 µM)	1.0 µL
Phusion HF 2x	20.0 µL

66) Run PCR reaction (98 ºC, 2 mins; 3 cycles of 98 ºC, 15 s; 70 ºC, 30 s; 72 ºC, 45 s; and 4 ºC on hold).
67) Purify reaction by standard Zymo concentrator-5 column protocol. Elute with 2x 8.5 µL of water and add 3 µL of Orange G loading dye.
68) Run a 6% native TBE gel at 200 V for 30 min, until the dye just ran off the gel. Loadind 50 bp ladder (NEB).
69) Stain gel in SYBR Gold for 3 min. Image gel at 0.5, 1, and 2 s exposure times. Cut out the DNA from 175 bp and above (corresponding to > 40 bp insert).
70) Use a syringe needle to punch a hole in the bottom of a 0.65 mL tube.
71) Transfer the gel slice to 0.65 mL tube and insert into a 2 mL collection tube. Spin at room temperature, 16,000X g for 5 min. The gel slice gets sheared into slurry by passing through the hole.
72) Remove the 0.65 mL tube and add 300 µL Gel elute buffer to the slurry. Shake at 55 ºC, 1000 rpm overnight in a thermomixer.
73) Pass the gel slurry through a Spin-X 0.45 µm column to recover the DNA library.
74) Add 5x volume of Zymo DNA binding buffer and flow-through Zymo concentrator-5 column. Wash with 200 µL Washing buffer once and elute twice with 8 µL water (recover ~15 µL library). Quantify library by a high sensitivity Bioanalyzer assay.
75) Barcoded libraries can be pooled together for sequencing if necessary.
76) Sequence the libraries on an Illumina sequencer using standard conditions and the P6_Custom_seqPrimer. Usually, a 70 nt single end sequencing reaction is enough for PARIS. The multiplexing and random barcodes are sequenced together with the insert.

Supplementary References

1. Chomczynski, P. & Sacchi, N. Single-step method of RNA isolation by acid guanidinium thiocyanate-phenol-chloroform extraction. *Anal Biochem* **162**, 156-159 (1987).
2. Chomczynski, P. (Google Patents, 1989).
3. Kim, Y.K., Yeo, J., Kim, B., Ha, M. & Kim, V.N. Short structured RNAs with low GC content are selectively lost during extraction from a small number of cells. *Molecular cell* **46**, 893-895 (2012).
4. Lu, Z., Gong, J. & Zhang, Q.C. PARIS: Psoralen Analysis of RNA Interactions and Structures with High Throughput and Resolution. *Methods in molecular biology* **1649**, 59-84 (2018).
5. Lu, Z. et al. RNA Duplex Map in Living Cells Reveals Higher-Order Transcriptome Structure. *Cell* **165**, 1267-1279 (2016).
6. Sastry, S.S., Ross, B.M. & P'Arraga, A. Cross-linking of DNA-binding proteins to DNA with psoralen and psoralen furan-side monoadducts. Comparison of action spectra with DNA-DNA cross-linking. *The Journal of biological chemistry* **272**, 3715-3723 (1997).
7. Sastry, S.S. et al. Laser-induced protein-DNA cross-links via psoralen furanside monoadducts. *Biochemistry* **32**, 5526-5538 (1993).
8. Zechel, K. & Weber, K. Degradation of nucleic acids in cell lysates by S1 nuclease in the presence of 9 M urea and sodium dodecylsulfate. *European journal of biochemistry / FEBS* **77**, 133-139 (1977).
9. Lapanje, S. Denaturation of globular proteins by guanidine thiocyanate. I. Optical rotation in aqueous guanidine thiocyanate solutions. *Biochimica et biophysica acta* **243**, 349-356 (1971).
10. Nwokeoji, A.O., Kilby, P.M., Portwood, D.E. & Dickman, M.J. Accurate Quantification of Nucleic Acids Using Hypochromicity Measurements in Conjunction with UV Spectrophotometry. *Anal Chem* **89**, 13567-13574 (2017).
11. Yoon, J.H. & Lee, C.S. Sequence specificity for DNA interstrand cross-linking induced by anticancer drug chlorambucil. *Arch Pharm Res* **20**, 550-554 (1997).
12. Tan, S.C. & Yiap, B.C. DNA, RNA, and protein extraction: the past and the present. *J Biomed Biotechnol* **2009**, 574398 (2009).
13. Ziv, O. et al. COMRADES determines in vivo RNA structures and interactions. *Nat Methods* **15**, 785-788 (2018).
14. Urdaneta, E.C. et al. Purification of cross-linked RNA-protein complexes by phenol-toluol extraction. *Nat Commun* **10**, 990 (2019).
15. Queiroz, R.M.L. et al. Comprehensive identification of RNA-protein interactions in any organism using orthogonal organic phase separation (OOPS). *Nat Biotechnol* **37**, 169-178 (2019).
16. Trendel, J. et al. The Human RNA-Binding Proteome and Its Dynamics during Translational Arrest. *Cell* **176**, 391-403.e319 (2019).
17. Shchepachev, V. et al. Defining the RNA interactome by total RNA-associated protein purification. *Mol Syst Biol* **15**, e8689 (2019).
18. Lu, Z. & Chang, H.Y. The RNA Base-Pairing Problem and Base-Pairing Solutions. *Cold Spring Harb Perspect Biol* **10** (2018).
19. Lu, Z., Carter, A.C. & Chang, H.Y. Mechanistic insights in X-chromosome inactivation. *Philos Trans R Soc Lond B Biol Sci* **372** (2017).
20. Sharma, E., Sterne-Weiler, T., O'Hanlon, D. & Blencowe, B.J. Global Mapping of Human RNA-RNA Interactions. *Mol Cell* **62**, 618-626 (2016).
21. Aw, J.G. et al. In Vivo Mapping of Eukaryotic RNA Interactomes Reveals Principles of Higher-Order Organization and Regulation. *Mol Cell* **62**, 603-617 (2016).
22. Wassarman, D.A. Psoralen crosslinking of small RNAs in vitro. *Mol Biol Rep* **17**, 143-151 (1993).

23. Xiao, J., Feehery, C.E., Tzertzinis, G. & Maina, C.V. E. coli RNase III(E38A) generates discrete-sized products from long dsRNA. *Rna* **15**, 984-991 (2009).
24. Dunn, J.J. RNase III cleavage of single-stranded RNA. Effect of ionic strength on the fideltiy of cleavage. *The Journal of biological chemistry* **251**, 3807-3814 (1976).
25. Gasparro, F.P., Saffran, W.A., Cantor, C.R. & Edelson, R.L. Wavelength dependence for AMT crosslinking of pBR322 DNA. *Photochem Photobiol* **40**, 215-219 (1984).
26. Spitale, R.C. et al. Structural imprints in vivo decode RNA regulatory mechanisms. *Nature* **519**, 486-490 (2015).
27. Sugimoto, Y. et al. hiCLIP reveals the in vivo atlas of mRNA secondary structures recognized by Staufen 1. *Nature* **519**, 491-494 (2015).
28. Vigne, R. & Jordan, B.R. Conformational analysis of RNA molecules by partial RNAse digestion and two dimensional acrylamide gel electrophoresis. Application to E. coli 5S RNA. *Biochimie* **53**, 981-986 (1971).
29. Zwieb, C. & Brimacombe, R. Localisation of a series of intra-RNA cross-links in 16S RNA, induced by ultraviolet irradiation of Escherichia coli 30S ribosomal subunits. *Nucleic acids research* **8**, 2397-2411 (1980).
30. Thompson, J.F. & Hearst, J.E. Structure of E. coli 16S RNA elucidated by psoralen crosslinking. *Cell* **32**, 1355-1365 (1983).
31. Lu, Z. et al. Metazoan tRNA introns generate stable circular RNAs in vivo. *Rna* **21**, 1554-1565 (2015).
32. Ruskin, B., Krainer, A.R., Maniatis, T. & Green, M.R. Excision of an intact intron as a novel lariat structure during pre-mRNA splicing in vitro. *Cell* **38**, 317-331 (1984).
33. Wang, X., Li, C., Gao, X., Wang, J. & Liang, X. Preparation of Small RNAs Using Rolling Circle Transcription and Site-Specific RNA Disconnection. *Mol Ther Nucleic Acids* **4**, e215 (2015).
34. Kladwang, W., Hum, J. & Das, R. Ultraviolet shadowing of RNA can cause significant chemical damage in seconds. *Scientific reports* **2**, 517 (2012).
35. Cadet, J. & Wagner, J.R. DNA base damage by reactive oxygen species, oxidizing agents, and UV radiation. *Cold Spring Harbor perspectives in biology* **5** (2013).
36. Cadet, J. & Douki, T. Formation of UV-induced DNA damage contributing to skin cancer development. *Photochem Photobiol Sci* **17**, 1816-1841 (2018).
37. Remsen, J.F., Miller, N. & Cerutti, P.A. Photohydration of uridine in the RNA of coliphage R17. II. The relationship between ultraviolet inactivation and uridine photohydration. *Proc Natl Acad Sci U S A* **65**, 460-466 (1970).
38. Pathak, M.A. Mechanisms of psoralen photosensitization reactions. *Natl Cancer Inst Monogr* **66**, 41-46 (1984).
39. Pathak, M.A. & Fitzpatrick, T.B. The evolution of photochemotherapy with psoralens and UVA (PUVA): 2000 BC to 1992 AD. *J Photochem Photobiol B* **14**, 3-22 (1992).
40. Banyasz, A. et al. Electronic excited states responsible for dimer formation upon UV absorption directly by thymine strands: joint experimental and theoretical study. *J Am Chem Soc* **134**, 14834-14845 (2012).
41. Liu, L., Pilles, B.M., Gontcharov, J., Bucher, D.B. & Zinth, W. Quantum Yield of Cyclobutane Pyrimidine Dimer Formation Via the Triplet Channel Determined by Photosensitization. *J Phys Chem B* **120**, 292-298 (2016).
42. Greenfeld, M., Solomatin, S.V. & Herschlag, D. Removal of covalent heterogeneity reveals simple folding behavior for P4-P6 RNA. *The Journal of biological chemistry* **286**, 19872-19879 (2011).
43. Selby, C.P. & Sancar, A. A cryptochrome/photolyase class of enzymes with single-stranded DNA-specific photolyase activity. *Proceedings of the National Academy of Sciences of the United States of America* **103**, 17696-17700 (2006).
44. Cimino, G.D., Shi, Y.B. & Hearst, J.E. Wavelength dependence for the photoreversal of a psoralen-DNA cross-link. *Biochemistry* **25**, 3013-3020 (1986).
45. Shi, Y.B. & Hearst, J.E. Wavelength dependence for the photoreactions of DNA-psoralen monoadducts. 1. Photoreversal of monoadducts. *Biochemistry* **26**, 3786-3792 (1987).
46. Shi, Y.B. & Hearst, J.E. Wavelength dependence for the photoreactions of DNA-psoralen monoadducts. 2. Photo-cross-linking of monoadducts. *Biochemistry* **26**, 3792-3798 (1987).
47. Thompson, J.F., Bachellerie, J.P., Hall, K. & Hearst, J.E. Dependence of 4'-(hydroxymethyl)-4,5',8-trimethylpsoralen photoaddition on the conformation of ribonucleic acid. *Biochemistry* **21**, 1363-1368 (1982).
48. Olmon, E. Solvent effects on photodegradation of the 18-mer of thymidylic acid. *OSU Knowledge Base* (2005).
49. Kleopfer, R. & Morrison, H. Organic photochemistry. XVII. Solution-phase photodimerization of dimethylthymine. *Journal of the American Chemical Society* **94**, 255-264 (1972).
50. Piskur, J. & Rupprecht, A. Aggregated DNA in ethanol solution. *FEBS Lett* **375**, 174-178 (1995).
51. Zubradt, M. et al. DMS-MaPseq for genome-wide or targeted RNA structure probing in vivo. *Nature methods* **14**, 75-82 (2017).
52. Zhao, C., Liu, F. & Pyle, A.M. An ultraprocessive, accurate reverse transcriptase encoded by a metazoan group II intron. *Rna* **24**, 183-195 (2018).
53. Mohr, S. et al. Thermostable group II intron reverse transcriptase fusion proteins and their use in cDNA synthesis and next-generation RNA sequencing. *Rna* **19**, 958-970 (2013).
54. Beukers, R. The effect of proflavine on U.V.-induced dimerization of thymine in DNA. *Photochem Photobiol* **4**, 935-937 (1965).
55. Setlow, R.B. & Carrier, W.L. Formation and destruction of pyrimidine dimers in polynucleotides by ultra-violet irradiation in the presence of proflavine. *Nature* **213**, 906-907 (1967).
56. Sutherland, B.M. & Sutherland, J.C. Inhibition of pyrimidine dimer formation in DNA by cationic molecules: role of energy transfer. *Biophys J* **9**, 1045-1055 (1969).
57. Sutherland, B.M. & Sutherland, J.C. Mechanisms of inhibition of pyrimidine dimer formation in deoxyribonucleic acid by acridine dyes. *Biophys J* **9**, 292-302 (1969).
58. Sutherland, J.C. & Sutherland, B.M. Ethidium bromide-DNA complex: wavelength dependence of pyrimidine dimer inhibition and sensitized fluorescence as probes of excited states. *Biopolymers* **9**, 639-653 (1970).

59. Aoki, S., Sugimura, C. & Kimura, E. Efficient Inhibition of Photo[2 + 2]cycloaddition of Thymidilyl(3‘−5‘)thymidine and Promotion of Photosplitting of the cis-syn-Cyclobutane Thymine Dimer by Dimeric Zinc(II)−Cyclen Complexes Containing m- and p-Xylyl Spacers. *Journal of the American Chemical Society* **120**, 10094-10102 (1998).
60. Setlow, J.K. & Setlow, R.B. Contribution of dimers containing cytosine to ultra-violet inactivation of transforming DNA. *Nature* **213**, 907-909 (1967).
61. Merriam, V. & Gordon, M.P. Pyrimidine dimer formation in ultraviolet irradiated TMV-RNA. *Photochem Photobiol* **6**, 309-319 (1967).
62. Greenstock, C.L., Brown, I.H., Hunt, J.W. & Johns, H.E. Photodimerization of pyrimidine nucleic acid derivatives in aqueous solution and the effect of oxygen. *Biochem Biophys Res Commun* **27**, 431-436 (1967).
63. Glazer, A.N. & Rye, H.S. Stable dye-DNA intercalation complexes as reagents for high-sensitivity fluorescence detection. *Nature* **359**, 859-861 (1992).
64. Gaugain, B., Barbet, J., Capelle, N., Roques, B.P. & Le Pecq, J.B. DNA Bifunctional intercalators. 2. Fluorescence properties and DNA binding interaction of an ethidium homodimer and an acridine ethidium heterodimer. *Biochemistry* **17**, 5078-5088 (1978).
65. Smith, C.A., Baeten, J. & Taylor, J.S. The ability of a variety of polymerases to synthesize past site-specific cis-syn, trans-syn-II, (6-4), and Dewar photoproducts of thymidylyl-(3'-->5')-thymidine. *J Biol Chem* **273**, 21933-21940 (1998).
66. Wheeler, E.C., Van Nostrand, E.L. & Yeo, G.W. Advances and challenges in the detection of transcriptome-wide protein-RNA interactions. *Wiley Interdiscip Rev RNA* **9** (2018).
67. Zhang, X., Rosenstein, B.S., Wang, Y., Lebwohl, M. & Wei, H. Identification of possible reactive oxygen species involved in ultraviolet radiation-induced oxidative DNA damage. *Free Radic Biol Med* **23**, 980-985 (1997).
68. Zhang, X. et al. Induction of 8-oxo-7,8-dihydro-2'-deoxyguanosine by ultraviolet radiation in calf thymus DNA and HeLa cells. *Photochem Photobiol* **65**, 119-124 (1997).
69. Nunomura, A. et al. Oxidative damage to RNA in neurodegenerative diseases. *J Biomed Biotechnol* **2006**, 82323 (2006).
70. Wamer, W.G. & Wei, R.R. In vitro photooxidation of nucleic acids by ultraviolet A radiation. *Photochem Photobiol* **65**, 560-563 (1997).
71. Rhee, Y., Valentine, M.R. & Termini, J. Oxidative base damage in RNA detected by reverse transcriptase. *Nucleic Acids Res* **23**, 3275-3282 (1995).
72. Bleeke, T., Zhang, H., Madamanchi, N., Patterson, C. & Faber, J.E. Catecholamine-induced vascular wall growth is dependent on generation of reactive oxygen species. *Circ Res* **94**, 37-45 (2004).
73. Goldstein, S. & Czapski, G. Mannitol as an OH. scavenger in aqueous solutions and in biological systems. *Int J Radiat Biol Relat Stud Phys Chem Med* **46**, 725-729 (1984).
74. Pelle, E. et al. Ultraviolet-B-induced oxidative DNA base damage in primary normal human epidermal keratinocytes and inhibition by a hydroxyl radical scavenger. *J Invest Dermatol* **121**, 177-183 (2003).
75. Silva-Júnior, A.C., Asad, L.M., Felzenszwalb, I. & Asad, N.R. Mutagenicity induced by UVC in Escherichia coli cells: reactive oxygen species involvement. *Redox Rep* **16**, 187-192 (2011).
76. Besaratinia, A., Kim, S.I., Bates, S.E. & Pfeifer, G.P. Riboflavin activated by ultraviolet A1 irradiation induces oxidative DNA damage-mediated mutations inhibited by vitamin C. *Proc Natl Acad Sci U S A* **104**, 5953-5958 (2007).
77. Darr, D., Combs, S., Dunston, S., Manning, T. & Pinnell, S. Topical vitamin C protects porcine skin from ultraviolet radiation-induced damage. *Br J Dermatol* **127**, 247-253 (1992).
78. Fröbel, S., Reiffers, A., Torres Ziegenbein, C. & Gilch, P. DNA Intercalated Psoralen Undergoes Efficient Photoinduced Electron Transfer. *J Phys Chem Lett* **6**, 1260-1264 (2015).
79. Fröbel, S., Levi, L., Ulamec, S.M. & Gilch, P. Photoinduced Electron Transfer between Psoralens and DNA: Influence of DNA Sequence and Substitution. *Chemphyschem* **17**, 1377-1386 (2016).
80. Jones, M.D. & Foulkes, N.S. Reverse transcription of mRNA by Thermus aquaticus DNA polymerase. *Nucleic Acids Res* **17**, 8387-8388 (1989).
81. Grabko, V.I., Chistyakova, L.G., Lyapustin, V.N., Korobko, V.G. & Miroshnikov, A.I. Reverse transcription, amplification and sequencing of poliovirus RNA by Taq DNA polymerase. *FEBS Lett* **387**, 189-192 (1996).
82. Myers, T.W. & Gelfand, D.H. Reverse transcription and DNA amplification by a Thermus thermophilus DNA polymerase. *Biochemistry* **30**, 7661-7666 (1991).
83. Bustin, S.A. Absolute quantification of mRNA using real-time reverse transcription polymerase chain reaction assays. *J Mol Endocrinol* **25**, 169-193 (2000).
84. Sellner, L.N. & Turbett, G.R. Comparison of three RT-PCR methods. *Biotechniques* **25**, 230-234 (1998).
85. Shi, C., Shen, X., Niu, S. & Ma, C. Innate Reverse Transcriptase Activity of DNA Polymerase for Isothermal RNA Direct Detection. *J Am Chem Soc* **137**, 13804-13806 (2015).
86. Alenko, A., Fleming, A.M. & Burrows, C.J. Reverse Transcription Past Products of Guanine Oxidation in RNA Leads to Insertion of A and C opposite 8-Oxo-7,8-dihydroguanine and A and G opposite 5-Guanidinohydantoin and Spiroiminodihydantoin Diastereomers. *Biochemistry* **56**, 5053-5064 (2017).
87. Furge, L.L. & Guengerich, F.P. Analysis of nucleotide insertion and extension at 8-oxo-7,8-dihydroguanine by replicative T7 polymerase exo- and human immunodeficiency virus-1 reverse transcriptase using steady-state and pre-steady-state kinetics. *Biochemistry* **36**, 6475-6487 (1997).
88. Van Nostrand, E.L., Shishkin, A.A., Pratt, G.A., Nguyen, T.B. & Yeo, G.W. Variation in single-nucleotide sensitivity of eCLIP derived from reverse transcription conditions. *Methods* **126**, 29-37 (2017).
89. Gerard, G.F. et al. The role of template-primer in protection of reverse transcriptase from thermal inactivation. *Nucleic Acids Res* **30**, 3118-3129 (2002).
90. Siegfried, N.A., Busan, S., Rice, G.M., Nelson, J.A. & Weeks, K.M. RNA motif discovery by SHAPE and mutational profiling (SHAPE-MaP). *Nat Methods* **11**, 959-965 (2014).
