# Supplementary material for: Optimized photochemistry enables efficient analysis of dynamic RNA structuromes and interactomes in genetic and infectious diseases
Source: Nat Commun. 2021 Apr 20;12:2344. doi: 10.1038/s41467-021-22552-y (PMC8058046; doi:10.1038/s41467-021-22552-y)
Supplement: Supplementary file 3 — Reporting Summary [file 41467_2021_22552_MOESM3_ESM.pdf]

nature research

Corresponding author(s): Zhipeng Lu

Last updated by author(s): Mar 3, 2021

Reporting Summary

Nature Research wishes to improve the reproducibility of the work that we publish. This form provides structure for consistency and transparency in reporting. For further information on Nature Research policies, see our Editorial Policies and the Editorial Policy Checklist.

Statistics

For all statistical analyses, confirm that the following items are present in the figure legend, table legend, main text, or Methods section.

n/a	Confirmed	
☐	☒	The exact sample size (*n*) for each experimental group/condition, given as a discrete number and unit of measurement
☐	☒	A statement on whether measurements were taken from distinct samples or whether the same sample was measured repeatedly
☐	☒	The statistical test(s) used AND whether they are one- or two-sided<br>*Only common tests should be described solely by name; describe more complex techniques in the Methods section.*
☒	☐	A description of all covariates tested
☐	☒	A description of any assumptions or corrections, such as tests of normality and adjustment for multiple comparisons
☐	☒	A full description of the statistical parameters including central tendency (e.g. means) or other basic estimates (e.g. regression coefficient) AND variation (e.g. standard deviation) or associated estimates of uncertainty (e.g. confidence intervals)
☐	☒	For null hypothesis testing, the test statistic (e.g. *F*, *t*, *r*) with confidence intervals, effect sizes, degrees of freedom and *P* value noted<br>*Give P values as exact values whenever suitable.*
☒	☐	For Bayesian analysis, information on the choice of priors and Markov chain Monte Carlo settings
☒	☐	For hierarchical and complex designs, identification of the appropriate level for tests and full reporting of outcomes
☐	☒	Estimates of effect sizes (e.g. Cohen's *d*, Pearson's *r*), indicating how they were calculated

Our web collection on statistics for biologists contains articles on many of the points above.

Software and code

Policy information about availability of computer code

Data collection	1. Nucleotides gels were acquired and analyzed by Bio-Rad Image System (Image Lab software, v6.0.1) and iBright FL1500 Image System (iBright Analysis Software, v3.1.2).<br>2. RNA profiles was acquired by 4200 TapeStation System (Agilent TapeStation Software v3.2).<br>3. qPCR data was acquired by ABI 7300 System (Software v1.3.1).<br>4. Primers and antisense oligos were designed by Primer 3 (https://bioinfo.ut.ee/primer3-0.4.0/).<br>5. PARIS2 reads sequences were acquired using Illumina instrumentation and software (Miseq and NovaSeq 6000 System, bcl2fastq2 Conversion Software v2.20.0).
Data analysis	All software and code used in this study has been described in published literature (Trimmomatic v0.36, STAR v2.7.0f, SAMtools v1.8, IGV v2.8.13, DSSR v1.7.7, GATK v4.1.9.0, Awk v4.2.0, bedtools v2.29.2, MSCULE v3.8.31, RNAz v2.0, R-scape v1.5.16, R-chie (https://www.e-rna.org/r-chie/), RNA2DMut (https://rna2dmut.bb.iastate.edu), ABI 7300 Software v1.3.1, ZEISS ZEN Imaging Software v3.2) or are custom scripts available on GitHub (https://github.com/zhipenglu/CRSSANT and https://github.com/minjiezhang-usc/PARIS2).

For manuscripts utilizing custom algorithms or software that are central to the research but not yet described in published literature, software must be made available to editors and reviewers. We strongly encourage code deposition in a community repository (e.g. GitHub). See the Nature Research guidelines for submitting code & software for further information.

Data

Policy information about availability of data

All manuscripts must include a data availability statement. This statement should provide the following information, where applicable:

- Accession codes, unique identifiers, or web links for publicly available datasets
- A list of figures that have associated raw data
- A description of any restrictions on data availability

The raw and processed PARIS2 sequencing data was deposited to Gene Expression Omnibus (GEO) with accession number GSE149493. Any other relevant data are available from the authors upon reasonable request. Source Data are provided with this paper.

Field-specific reporting

Please select the one below that is the best fit for your research. If you are not sure, read the appropriate sections before making your selection.

☒ Life sciences ☐ Behavioural & social sciences ☐ Ecological, evolutionary & environmental sciences

For a reference copy of the document with all sections, see nature.com/documents/nr-reporting-summary-flat.pdf

Life sciences study design

All studies must disclose on these points even when the disclosure is negative.

Sample size	No sample size calculations were performed. Sample size was determined to be adequate based on the magnitude and consistency of measurable differences between groups.
Data exclusions	No data was excluded from analysis.
Replication	Each experiments was performed independently at least two times. All experiments were highly reproducible.
Randomization	The cells were randomly assigned to treated or no-treated for this study.
Blinding	The investigators were not blinded during data collection since they were in vivo studies in which the treat groups needed to be clear when performing the experiments. However, PARIS2-seq samples were processed by separate scientists and each data set was analyzed by separate bio-informaticians.

Reporting for specific materials, systems and methods

We require information from authors about some types of materials, experimental systems and methods used in many studies. Here, indicate whether each material, system or method listed is relevant to your study. If you are not sure if a list item applies to your research, read the appropriate section before selecting a response.

Materials & experimental systems

n/a	Involved in the study	
☐	☒	Antibodies
☐	☒	Eukaryotic cell lines
☒	☐	Palaeontology and archaeology
☐	☒	Animals and other organisms
☒	☐	Human research participants
☒	☐	Clinical data
☒	☐	Dual use research of concern

Methods

n/a	Involved in the study	
☒	☐	ChIP-seq
☒	☐	Flow cytometry
☒	☐	MRI-based neuroimaging

Antibodies

Antibodies used	1, Anti-Enterovirus D68 VP1 antibody (Polyclonal antibody, GeneTex, GTX132313)<br>2. Goat anti-Rabbit IgG (H+L) Cross-Adsorbed Secondary Antibody (Polyclonal antibody, Thermo Fisher, R-6394)
Validation	1. Xu N et al. J Virol 2020; 94 (9) The Pyrimidine Analog FNC Potently Inhibits the Replication of Multiple Enteroviruses.<br>2. Leow SM et al. Oncotarget 2017; 8(10). Sub-lethal oxidative stress induces lysosome biogenesis via a lysosomal membrane permeabilization-cathepsin-caspase 3-transcription factor EB-dependent pathway.

Eukaryotic cell lines

Policy information about cell lines

Cell line source(s)	HEK293T (CRL-3216), HeLa (CCL-2) and SH-SY5Y (CRL-2266) are purchased from ATCC.
Authentication	Cell lines were not authenticated but verified by morphological characteristics.
Mycoplasma contamination	No mycoplasma contamination was detected in these cells.
Commonly misidentified lines (See ICLAC register)	No commonly misidentified cell line was used.

Animals and other organisms

Policy information about studies involving animals; ARRIVE guidelines recommended for reporting animal research

Laboratory animals	Wild-type C57BL/6J mice were bred and maintained under specific pathogen-free conditions, fed standard laboratory chow, and kept on a 12-h light/dark cycle and temperature and humidity were kept at 22±1°C, 55%±5%. C57BL/6J female or male mice aged 4-6 weeks old were used for all experiments.
Wild animals	No wild animals were used in this study.
Field-collected samples	No field collected samples were used in this study.
Ethics oversight	All animals were used according to animal use protocols granted by the Institutional Animal Care and Use Committee at the University of Southern California.

Note that full information on the approval of the study protocol must also be provided in the manuscript.
